# Physics-Informed Deep Learning Approach for Reintroducing Atomic Detail in Coarse-Grained Configurations of Multiple Poly(lactic acid) Stereoisomers

**DOI:** 10.1021/acs.jcim.3c01870

**Published:** 2024-03-01

**Authors:** Eleftherios Christofi, Petra Bačová, Vagelis A. Harmandaris

**Affiliations:** † Computation-based Science and Technology Research Center, The Cyprus Institute, Nicosia 2121, Cyprus; ‡ Departamento de Ciencia de los Materiales e Ingeniería Metalúrgica y Química Inorgánica, Facultad de Ciencias, IMEYMAT, Campus Universitario Río San Pedro s/n., Puerto Real, Cádiz 11510, Spain; § Department of Mathematics and Applied Mathematics, University of Crete, Heraklion GR-71110, Greece; ∥ Institute of Applied and Computational Mathematics, Foundation for Research and Technology - Hellas, Heraklion GR-71110, Crete, Greece

## Abstract

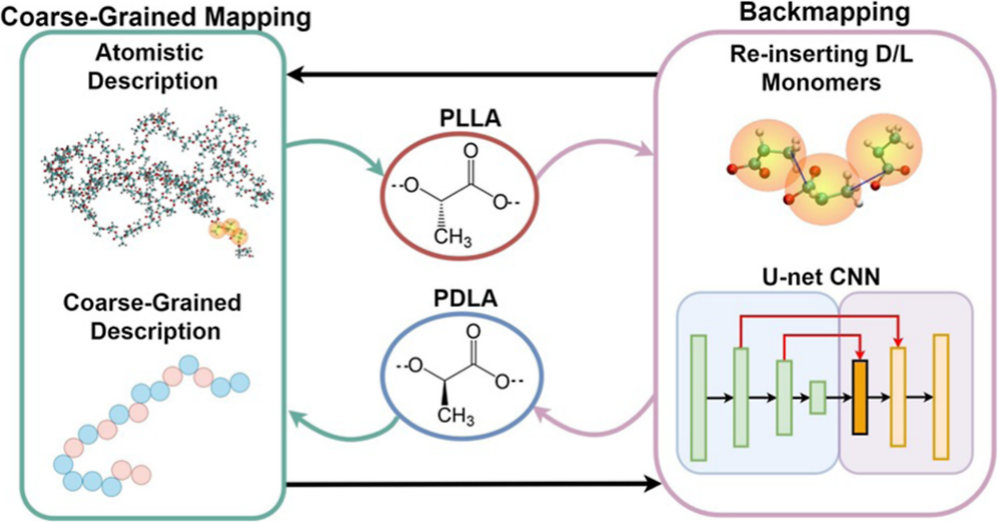

Multiscale modeling of complex molecular systems, such as macromolecules, encompasses methods that combine information from fine and coarse representations of molecules to capture material properties over a wide range of spatiotemporal scales. Being able to exchange information between different levels of resolution is essential for the effective transfer of this information. The inverse problem of reintroducing atomistic degrees of freedom in coarse-grained (CG) molecular configurations is particularly challenging as, from a mathematical point of view, it is an ill-posed problem; the forward mapping from the atomistic to the CG description is typically defined via a deterministic operator (“one-to-one” problem), whereas the reversed mapping from the CG to the atomistic model refers to creating one representative configuration out of many possible ones (“one-to-many” problem). Most of the backmapping methods proposed so far balance accuracy, efficiency, and general applicability. This is particularly important for macromolecular systems with different types of isomers, i.e., molecules that have the same molecular formula and sequence of bonded atoms (constitution) but differ in the three-dimensional configurations of their atoms in space. Here, we introduce a versatile deep learning approach for backmapping multicomponent CG macromolecules with chiral centers, trained to learn structural correlations between polymer configurations at the atomistic level and their corresponding CG descriptions. This method is intended to be simple and flexible while presenting a generic solution for resolution transformation. In addition, the method is aimed to respect the structural features of the molecule, such as local packing, capturing therefore the physical properties of the material. As an illustrative example, we apply the model on linear poly(lactic acid) (PLA) in melt, which is one of the most popular biodegradable polymers. The framework is tested on a number of model systems starting from homopolymer stereoisomers of PLA to copolymers with randomly placed chiral centers. The results demonstrate the efficiency and efficacy of the new approach.

## 1 Introduction

Atomistic molecular dynamics (MD) simulations provide information about the dynamic evolution of a molecular system and condensed matter at the atomic level. Despite the modern advances in the available computational resources, the length and time scales accessible by atomistic models are still limited. This is particularly important for complex polymer-based materials, which are characterized by an enormous range of characteristic spatiotemporal scales. For example, phenomena in the atomic scale occur within a few angstroms (about the size of a monomer), whereas nanostructured domains involve several nanometers or even millimeters for macroscopic structures. At the same time, associated temporal scales range from the level of a few picoseconds, or even femtoseconds, relevant for the fast dynamics at the atomic/segmental level up to seconds or even days characterizing long-time phenomena and collective dynamics.^1,2^ To expand the range of accessible scales via atomistic simulations coarse-grained (CG) models that reduce the dimensionality of the physical system under study have been developed.^2−7^ In systematic, bottom-up CG models, groups of atoms are lumped together into particles that are typically denoted as “superatoms” or beads.^6,8,9^ Currently, such approaches are applied to a multitude of molecular systems, such as proteins and synthetic polymers; by simultaneously considering models at different scales, one targets developing an approach that shares the computational efficiency of the coarser models, as well as the accuracy of the microscopic (finer) ones.^7,10,11^

The CG models can provide direct information about the behavior of the physical system for length scales on the order of the size of the CG beads (e.g., around the size of one monomer for a macromolecule) and above. However, to get information about properties that depend on the microscopic (e.g., monomeric) structure, atomic-level resolution is necessary. Therefore, accurate and computationally efficient backmapping schemes, introducing atomic detail in the CG structures, are essential for closing the loop, which starts from a given atomistic model, proceeds with deriving a systematic bottom-up CG model, and then goes back to the detailed atomistic description by reinserting the atoms into the CG particles.

A number of studies have been conducted for synthetic polymers as well as biobased materials in order to address the aforementioned challenging problem using computational approaches based on random mapping, geometrical and mechanical considerations, and position-restrained MD or Monte Carlo simulations.^12−27^ These models mostly rely on maintaining libraries of molecular structures or force fields that are system-specific while often balancing efficiency and accuracy. More recently, a number of approaches based on advanced machine learning (ML) methods (e.g., generative adversarial networks,^28,29^ autoencoders,^30^ transformers,^31^ Gaussian process regression, and random forests^32^) have also been explored; though, in general, so far they are usually demonstrated on very specific classes of problems and/or molecules of limited size, typically concerning hydrocarbon polymers or proteins.

Synthetic biodegradable polymers share some similarities with both common synthetic polymers and proteins. Like proteins, their structure is usually complex, involving critical intermolecular interactions between molecules, which makes their study computationally challenging. Unlike proteins, there is no openly available database of a wide range of polymer structures that could be used for data-driven approaches. On the other hand, synthetic biodegradable polymers are produced by conventional polymerization methods, as in the case of well-known industrial polymers, which allows for better control over produced structures.^33^ Poly(lactic acid) (PLA) represents one of the most popular biodegradable polymers due to its high potential in the packaging industry^34^ and wide usage in additive manufacturing.^35^ PLA consists of monomers with chiral centers, and therefore, it exists in three stereoisomeric forms: poly(l-lactide) acid (PLLA), poly(d-lactide) acid (PDLA) and poly(dl-lactide) acid (PDLLA). The popularity of PLA is also reflected in the high number of computational methods which have been used so far to describe the polymer structure at multiple length and time scales (see, e.g., the recent review by Vasilevskaya and co-workers^36^). Regarding CG models of PLA, two types of mapping have been reported: “A-*graft*-B” mapping corresponding to two beads per monomer^37,38^ and 1:1 mapping with one of the oxygens acting as the center of the CG bead.^39^ Notably, none of the above CG models have accounted for stereochemistry, as they have been exclusively focused on the PLLA. The authors of the “A-*graft*-B” model also presented a backmapping algorithm in which the inserted monomeric units were connected through an overlapping backbone carbon. The monomers were inserted sequentially and were rotated during the procedure to align with the corresponding axis of the CG configuration.^40^ Note that the above CG studies,^37,38^ as well as the backmapping procedure,^40^ dealt with the oligomeric PLA. Guseva et al. have also reported data from atomistic simulations for PLLA and PDLLA; however, the study was limited to one molecular weight of PDLLA copolymer.^41^

Here, we provide an accurate and efficient computational backmapping methodology for obtaining all-atom PLA-based macromolecular systems that is applicable to other chiral copolymers as well. We use an all-atom (AA) description for the polymer systems instead of a united-atom (UA) one to fully consider the atomic detail. Our method is based on a recent backmapping machine learning (ML) algorithm developed for UA models that learns the conditional distribution function of united-atom configurations, given the CG ones, using U-net convolutional neural networks (CNN).^42^ As an example of a chiral polymer of great technological interest, we apply the developed approach in order to reinsert the atomic detail into the CG configurations of PLLA, PDLA, and PDLLA. The objective of this study is 2-fold: first, to develop an effective and versatile algorithm for backmapping of chiral molecules at the all-atom scale and second, to go beyond the currently available computational studies of PLA by designing a tool capable of producing atomistic configurations of PLA with multiple molecular weights and compositions. In order to achieve these goals, first we created a training set by performing extensive atomistic MD simulations. Then, we tested the chemical transferability of the ML-based model on different types of PDLLA copolymers and the transferability across molecular weight on unentangled stereoisomers of PLA. The efficiency of this ML approach is examined, and the prediction quality is evaluated by applying the trained model to data outside of the training sets.

In the next section, we present in a thorough and comprehensive manner the proposed methodology, while in the section Atomistic and Coarse-Grained Poly(lactic acid) Models and Simulations, an overview of the atomistic and CG models is given. In the section Physics-Informed Deep Learning Model, we describe in detail the architecture of the CNN utilized for the implementation of the method. Finally, in the Results section, we provide an in depth evaluation of the predicted atomistic configurations of the developed models.

## 2 Data Learning Methodology Across Scales

In this section, we provide a comprehensive discussion about the atomistic and CG descriptions, as well as the development of the U-net-based CNNs that have the ability of reinserting atomistic detail into CG macromolecular models. To apply the method, we use proper chemical descriptors derived from the atomic structure of the underlying physical system, i.e., atom coordinates and the chemical bonds among connected pairs of atoms.

**Figure 1:**
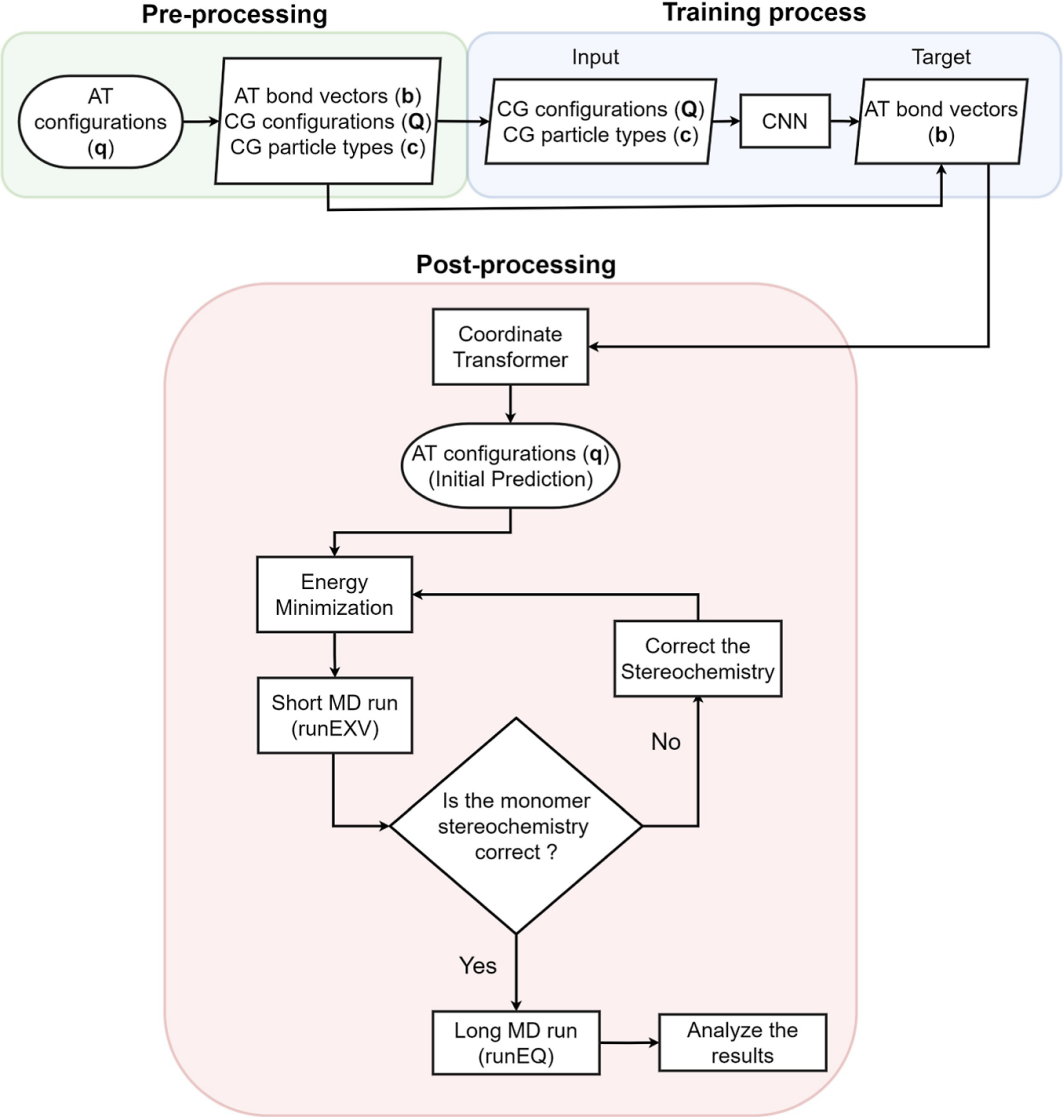
Schematic representation of the backmapping method. The preprocessing and training process are described in section Data Learning Methodology Across Scales, and the postprocessing part is explained in section PLA Configurations Derived from the Deep Learning Backmapping Algorithm.

A graphical representation of the entire methodology is shown in Figure 1, which comprises the preprocessing, training, and postprocessing stages. In the preprocessing part, we gather data about the probability distribution functions of the atomistic descriptors, i.e., atomistic (AT) bond vectors (**b**), as target quantities, conditioned on the coordinates of the CG particles (**Q**) and their corresponding types (**c**), which serve as input data. Moving to the training phase, we train the model to produce samples from the given probability distribution, *P*(**b**|**Q**, **c**), which gives us the ability to penalize critical geometrical properties of the system under study such as bond lengths, bond angles, and dihedral angles. Moreover, in the postprocessing stage, we further process the generated samples to obtain the atomistic configurations in the Cartesian space, i.e., to get *P*(**q**|**Q**, **c**), where **q** represents the atomistic coordinates. Finally, the initial prediction was processed to acquire the desired stereochemistry for the given configuration.

### 2.1 Atomistic and Coarse-Grained Description

Before discussing the learning process, we provide an outline of the systematic methodology for deriving CG models using data obtained from detailed atomistic ones. Suppose a prototypical multicomponent macromolecular system comprising *N* microscopic particles confined within a box of volume *V* at temperature *T*. The set of coordinates for the *N* atoms with potential energy *U*(**q**) is represented by **q** = . The probability of a state **q** at temperature *T* is given via the Gibbs canonical measure
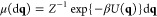
1where  is the partition function, , and *k*_B_ is the Boltzmann constant.

From a mathematical point of view, coarse-grained modeling is considered a form of dimensionality reduction by applying a mapping (CG mapping) 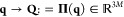
2on the microscopic state space, determining the *M*(<*N*) CG particles as a function of the atomic configuration **q**. We denote by  any point in the CG space. We refer to the elements of the microscopic space with positions  as atomistic particles and the elements of the CG space with positions , *i* = 1, ..., *M* as “CG particles”.

The most commonly used mappings in coarse graining of molecular systems are linear ones represented by a set of non-negative real constants for each CG particle *i*, of the form ζ_*ij*_, (*i* = 1, ..., *M*, *j* = 1, ..., *N*),^3,43^ for which
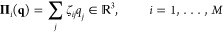
3

### 2.2 Statistical Descriptors and Learning Model

Backmapping algorithms target generating atomistic coordinates from a given CG configuration. To achieve high-fidelity all-atom data, the statistical relationships between the CG and atomistic configurations need to be captured. For example, the arrangement of atom positions in a polymer chain should follow the correlations among successive CG beads along the chain contour, as expressed by bond distances, angles, dihedrals, etc. In the current work, we use a standard linear CG mapping, Π_*i*_(**q**), in which the center of mass of each monomer represents the position of a CG particle (1:1 mapping), i.e., the CG coordinates are obtained by
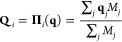
4where *M*_*j*_ is the mass of the *j*th particle of the *i*th monomer and the sum is over all atoms within a monomer. Moreover, with the integer vector , we denote the type of each CG particle, where *c*_*i*_ ∈ {1, 2, ..., *k*} and *k* is the total number of CG particle types in the CG configurations. Therefore, we can express the back-mapping procedure as sampling from the conditional probability *P*(**q**|**Q**, **c**).

**Figure 2:**
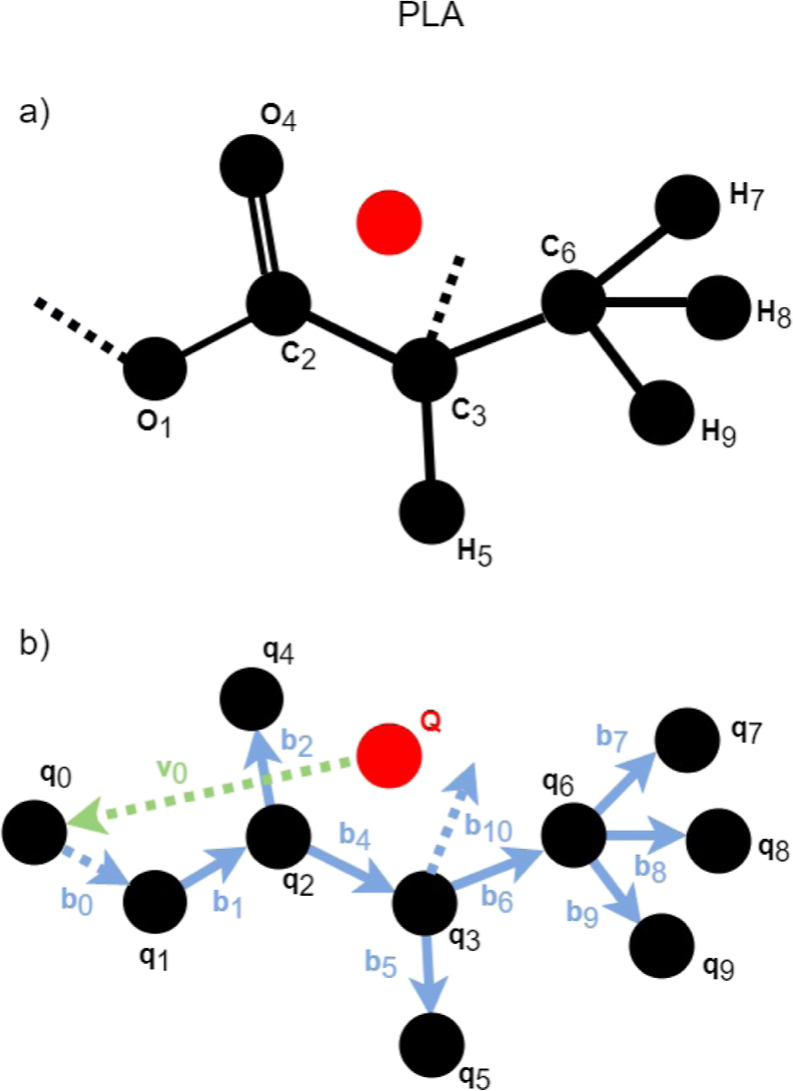
(a) Graphical representation of a PLA monomer, where the dashed lines show the bonds between the neighboring monomers and the red isolated dot indicates the position of the CG particle. (b) Monomer-based representation scheme for a PLA monomer, where the blue solid vectors denote the bond vectors (**b**_*i*_), the green dashed vector (**v**_*i*_) indicates the relative coordinates of the atoms with respect to the center of mass of the monomer, and **q**_0_ denotes the coordinates of the last atom of the previous monomer.

At the same time, the proposed deep learning (DL)-based backmapping method should respect the symmetries imposed by the physical law; e.g., it should be invariant with respect to translation and rotation in Cartesian space. Thus, particles were referenced by vectors that depict relative positions within the individual CG bead. As can be seen in Figure 2b, we utilize bond vectors among connected pairs of atoms as the data representation scheme. Hence, we formulate the problem as a training over the conditional probability *P*(**b**|**Q**, **c**), with , where *K* is the total number of chemical bonds in the system, which is of the order of the number of all atoms in the system, *N*.

### 2.3 Introducing Physical Prior in Learning

One of the most crucial features of the proposed backmapping method is the ability to introduce physical prior in the loss function. Targeting directly the **b** vectors allows us to improve the prediction of the initial backmapped configuration through penalizing geometrical properties related to the system. First, in the cost function a term that penalizes the bond vectors among the connected pair of atoms is used, as
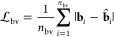
5where **b**_*i*_ is the output of the neural network (reconstructed vector),  is the target output, and *n*_bv_ is the total number of **b**_*i*_ vectors.

In addition, we can expand the cost function, thus improving the training of the deep learning model, by adding additional physics-based chemical characteristics such as information about the distributions of bond lengths, bond angles, and dihedral angles, which can be expressed as functions of atom coordinates (or coordinates of the chemical bonds). This is rather straightforward since we target bond vectors among connected pairs of atoms instead of the absolute Cartesian coordinates of the atoms. Hence, the following terms are integrated in the cost function
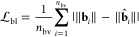
6
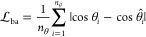
7
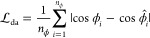
8

In the above relations, with ∥**b**_*i*_∥ we denote the magnitude of **b**_*i*_ vectors, whereas θ (*i* = 1, 2, ..., *n*_θ_) and ϕ (*i* = 1, 2, ..., *n*_ϕ_) denote the set of atomistic bending angles and dihedral (torsional) angles, respectively. *n*_θ_ and *n*_ϕ_ refer to the number of all bending and dihedral angles defined from the chemical topology (monomeric structure) of the model system under study.

Last, we also include a penalty term for the **v**_0_ vectors (see Figure 2b) via
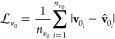
9where  is the number of **v**_0_ vectors.

Overall, we defined the loss function of the network as a linear combination of the above metrics
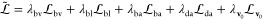
10where λ are the corresponding weights for each term; here, we treat the λ values as tunable parameters (hyperparameters).

## 3 Atomistic and Coarse-Grained Poly(lactic acid) Models and Simulations

**Figure 3:**
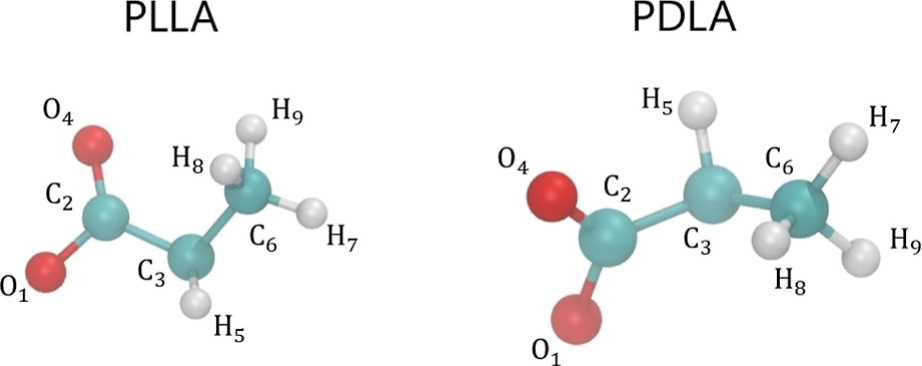
Snapshots of PLLA and PDLA monomers with the corresponding notation of the atoms. Each monomer is connected with the neighboring monomers via atoms O_1_ and C_3_.

As an illustrative example of chiral biodegradable polymers, we apply the developed algorithm to amorphous PLA polymers, which may contain two types of stereoisomers, PDLA and PLLA; Figure 3 depicts the 2 different monomer types.

The development and implementation of our deep learning backmapping algorithm are based on an extensive, synthetic data set consisting of PLA configurations derived from atomistic MD simulations. Details about the different atomistic systems can be seen in Table 1. The first column of the table denotes the different uses of each system: with A we indicate the 100-mer PLA systems used for the training of the backmapping models, while B refers to the 30-mer systems utilized to probe the transferability across molecular length of the corresponding trained models. All copolymers are random, i.e., the chosen percentage of the monomers with the given stereochemistry is distributed randomly along the chain. Each chain of the 100-mer copolymer system has an identical sequence of chiral monomers, i.e., the distribution of the chiral monomers along the chain is random, but their placement is identical for all 70 chains in the box. The 30-mer copolymer consists of chains whose sequences are different, but the content of the d-stereoisomer is kept fixed per chain. As we mentioned in the previous section, the mapping from the atomistic  to the CG  representation converts each monomer to one CG particle, where *N* and *M* represent the total number of atomistic and CG particles in the system, respectively. Specifically, we place a CG bead at the center of mass of each monomer (Figure 2a).

**Table 1:** Details of the Model (Atomistic and CG) Systems^a^

group	label	chains	atoms	CG particles	monomers per chain	microstructure	
						PLLA (%)	PDLA (%)
A	PLLA100	70	63,210	7000	100	100	0
A	PDLA100	70	63,210	7000	100	0	100
A	Copo100	70	63,210	7000	100	45	55
B	PLLA30	70	19,110	2700	30	100	0
B	PDLA30	70	19,110	2700	30	0	100
B	Copo30	70	19,110	2700	30	84	16

a The groups in the first column represent different data sets. The group A was used for the training of the ML models, while the group B was utilized to probe the transferability of the model across different molecular lengths. Each chain in the 100-mer copolymer system has an identical sequence of chiral monomers. The 30-mer copolymer consists of random copolymers with a different sequence but with a fixed content of d monomer per chain.

The atomistic simulations were performed by the GROMACS package.^44^ We chose an all-atom representation using the PLAFF3 force field^45^ to model the PLA chains under study. The systems were prepared as follows: the starting point was a partially equilibrated configuration of 70 chains of a 500-mer PLLA in melt, which was prepared from a published configuration of three 500-mer chains,^45^ following a procedure consisting of various short *NPT* simulations similar to that in ref (46). The length of the chains in this initial system was adjusted to get a molecular weight of 100-mer PLLA, and then the so-obtained configuration was equilibrated. The 30-mer PLLA was also prepared by shortening the chains, but the starting configuration was an equilibrated configuration of 100-mer PLLA. In order to get the d-stereoisomer and the copolymer with the given sequence of d-component, we changed the stereochemistry of a randomly selected PLLA chain by switching the positions of the H_5_ atom and the methyl group (see Figure 3). Then we randomly placed 70 chains in a box and proceeded with the equilibration.

We should also note here that the equilibration process in polymer systems is among the most time-consuming simulation steps due to the high molecular weights of polymers. A system is fully equilibrated when its time-averaged properties do not depend on its initial state.^47^ In order to achieve that the molecule must displace significantly in the box (achieving intermixing) and/or its structure must be decorrelated. This is particularly challenging for macromolecular systems for which the relaxation (decorrelation) time scales exponentially with the molecular weight, with the exponent 2 for low and 3.4 for high molecular weights.^1^

**Figure 4:**
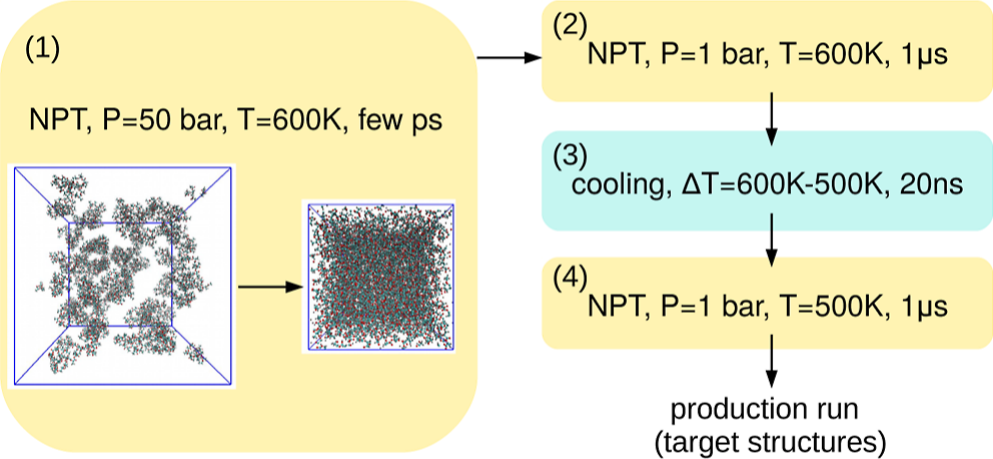
Equilibration procedure of the atomistic PLA-based systems. During the step 1 the box is squeezed to eliminate the voids created by the random placement of the molecules. The notation denotes the type of the run, the simulation conditions and the length of each run. *T* stands for temperature and *P* for pressure.

To address the above challenge, we follow the systematic multistage equilibration methodology of PLA systems illustrated in Figure 4. First, we eliminate heterogeneities in density (step 1), then we equilibrate the system at a higher temperature (step 2) to speed up the chain intermixing, and then we cool the system to the desired temperature (step 3), followed last by additional simulations under constant temperature and pressure (step 4).

In all steps of the equilibration procedure, the Berendsen barostat and v-rescale thermostat were used, the LINCS algorithm was applied to constrain the bonds, electrostatic interactions were approximated by the cutoff scheme with a cutoff distance of 1 nm, and the time step was 2 fs. In the case of PLLA systems, which were prepared from partially (or fully) equilibrated configurations of longer chains, step 1 was not necessary and steps 2 and 4 were shorter (hundreds of nanoseconds, depending on the chain length, see above). During steps 2 and 4, we monitored the fluctuations of the radius of gyration to ensure proper equilibration. After the equilibration, we run a preproduction run of 1 μs with the same settings as the production run. More specifically, the Nose–Hoover thermostat kept the temperature at 500 K, the Parrinello–Rahman barostat maintained the pressure at 1 atm, and the PME method was used for the electrostatic interactions. The production runs were 1 μs long, and the configurations were saved every 200 ps, thus collecting 5000 representative snapshots for the training set.

## 4 Physics-Informed Deep Learning Model

We continue with details about the implementation of the deep learning backmapping algorithm. For computational efficiency, the DL model makes predictions for a specific (integer) number of CG particles (here, PLA monomers) at a time, s. As can be seen in Table 1, the systems used for the training of the neural network (NN) consist of 100-mer chains, i.e., 100 CG particles per chain. In this implementation for the backmapping from the CG to the atomistic description, we choose to give as input to the CNN information about a single chain (903 atoms). Therefore, for simplicity we choose *s* = 100, but a different *s* value can be chosen. Moreover, a system with shorter chains can be treated by extra zero-padding in the input, while longer chains can be processed by consecutive fragments of size *s* or a different one if necessary. Taking into consideration that the input of the CNN should be a power of 2, the input shape is (1024,*k* + 3) and the output shape is (1024,3), where 903 spots contain information about the chain and the rest are filled with zero-padding. With *k* we denote the number of different monomer types we have in the system, which we represent as one-hot vectors. We note that we treat the first and the last monomers of the chain as additional monomer types due to their different structures compared to the other monomers. Therefore, for a training set that consists of a homopolymer system we have *k* = 3, for a copolymer system *k* = 4 due to the fact that we have the same monomer types for the first and last monomers of every chain, while for a model trained with both homopolymer and copolymer systems *k* = 6.

**Figure 5:**
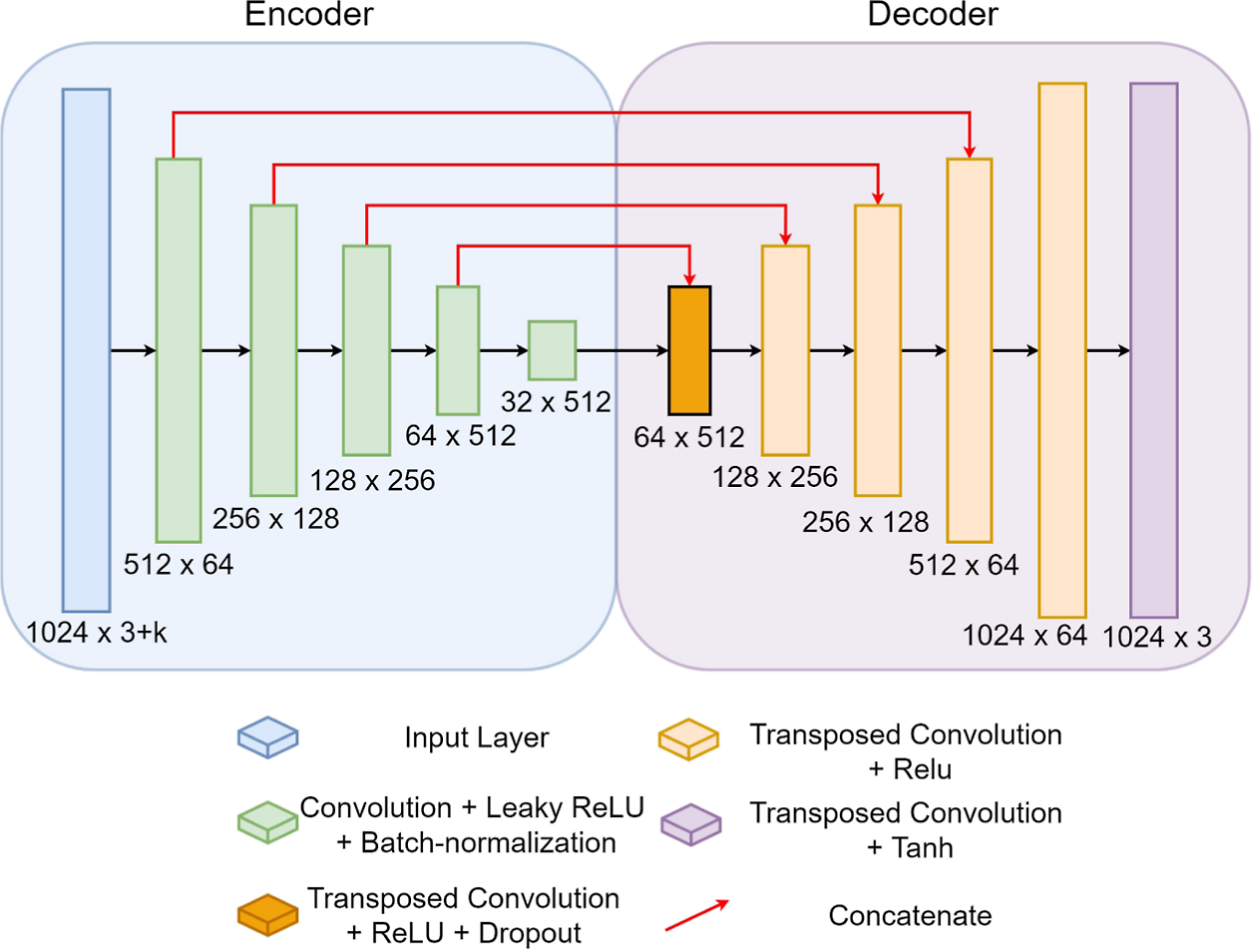
Schematic representation of the CNN used for the implementation of the method.

We utilize a U-net CNN based model, shown in Figure 5, which consists of an encoder and a decoder network with skip connections among them. For the encoder, we stack five down-sample blocks, which consist of a convolution layer with stride 2, a leaky ReLU activation function, and a batch-normalization layer. We note that we start with 64 filters and end up with 512. Then we pass the output of the encoder to the decoder network, where we have five up-sample blocks, which consist of a transposed convolution layer with stride 2 and a ReLU activation function. For the first up-sample block, we have a dropout layer with a rate of 0.5. We note that for the last layer of the network we have a transposed convolution layer with stride 1 and a tanh activation function because we rescale the target output values in the interval [−1,1].

Furthermore, for the training process, we utilize mini-batch gradient descent with batches of size 64 and Adam optimization algorithm with an initial learning rate of 0.001, which was decreased down to 0.000001 by a factor of 8 once learning stagnated. The CNN was implemented in the open-source TensorFlow 2 platform.^48^ The computational time needed to train the model on a single NVIDIA Tesla V100-SXM2 GPU for 1000 epochs was around 24 h.

As mentioned in the section Atomistic and Coarse-Grained Poly(lactic acid) Models and Simulations, we have three different data sets utilized for the training and testing of the models. Two of them consist of homopolymer systems (PDLA and PLLA), while the other one is a copolymer PLA system with a stereochemistry of 45% PLLA and 55% PDLA. Therefore, we obtain three different models trained with each of the aforementioned data sets. Having a total of 5000 frames for each system, we split our data into 80% for the training set, 10% for the validation set, and 10% for the test set. In addition to these three models, we trained another one by utilizing all of the data we have at our disposal and then using it to probe the chemical transferability of the algorithm.

In the section Introducing Physical Prior in Learning, we described a number of different metrics that we want to minimize during the training of the network. Based on these, we defined the loss function of the network as a linear combination of those metrics (see eq 10), where λ are the corresponding weights for each term; here, we treat the λ values as hyperparameters. After performing a number of test runs with different sets of λ values, where we examined the quality of the predicted configurations based on a number of different distributions, such as bond lengths, bond angles, dihedral angles, radial distribution function, and internal distances, while also taking into account the number of “wrong” monomers (see the section Results), we concluded that, overall, the best model is the one that only penalizes bond vectors and bond lengths. Thus, we set λ_bv_ = λ_bl_ = 1 and  = λ_ba_ = λ_da_ = 0 for future runs. A comparison between the model used for future runs and a model where we only penalize the bond vectors (λ_bv_ = 1 and  = λ_bl_ = λ_ba_ = λ_da_ = 0) is given in the section Bond Vectors Model in Supporting Information. We note that after the model was trained, the computational time needed to reconstruct an atomistic configuration on an Intel Core i7-10750H CPU, was around 7 s.

**Figure 6:**
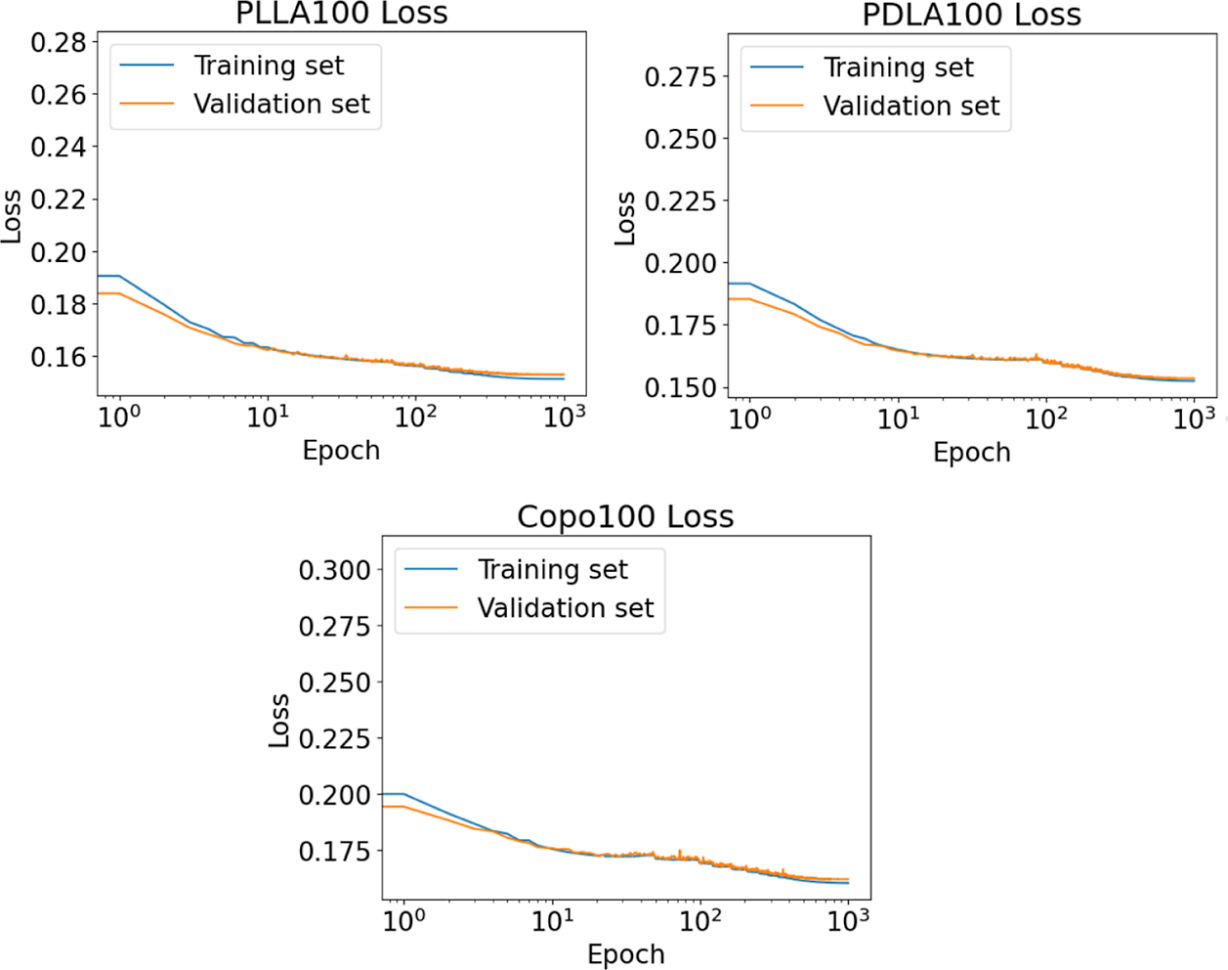
Value of loss functions as a function of epochs for the training and validation set of PLLA100, PDLA100, and Copo100.

In addition, Figure 6 depicts the loss functions for the training and validation sets of the three aforementioned systems (PLLA100, PDLA100, and Copo100), where it is clear that all of the loss functions converged to their corresponding (local) minimum almost at the same number of epochs. Moreover, the values of the loss functions for both the training and the validation sets are very close, which indicates that overfitting is avoided.

## 5 Results

In this section, we discuss the performance of the trained DL models by investigating their accuracy concerning the predicted atomistic structure of the PLA systems shown in Table 1. Next, we also examine in detail the transferability of the DL models across the chemical composition and the molecular weight of the PLA copolymers.

### 5.1 PLA Configurations Derived from the Deep Learning Backmapping Algorithm

First, we provide details about the backmapping procedure, which includes a short sequence of atomistic simulations and customized codes examining the stereochemistry of the derived all-atom configurations, as illustrated in Figure 1. The various steps were carefully developed and tested to obtain the desired atomistic structure from the predicted configurations by the trained models; below, we briefly summarize the technical details. After obtaining the backmapped all-atom PLA structure, we perform energy minimization via a steepest descent algorithm, which in our case takes negligible computational time (around 7 s) on an Intel Xeon Gold 6248 CPU. Such an energy minimization is a rather standard approach for all backmapping numerical methods due to the nonunique solution of the ill-posed reverse problem.^12,13,15−19,22,49^ After the energy minimization, we perform a very short simulation (labeled as runEXV in Figure 1) of 0.1 ps with a temperature of *T* = 300 K maintained by the algorithm using velocity rescaling with a stochastic term and a pressure of *P* = 1 atm regulated by the Berendsen barostat. The time step of 0.01 fs was set to slowly introduce the excluded volume according to the selected force field. The computational time needed to perform the runEXV simulation is around 166 s, using 32 processors on an Intel Xeon Gold 6248 CPU, which can be further reduced by increasing the number of processors.

Due to the complexity of the backmapping problem, the trained model might misplace the H_5_ in a direction parallel to the main backbone (see Figure 3). Therefore, after applying the force field, due to the excluded volume, the H_5_ is shifted to the nearest empty place, which results in some cases in a wrong stereochemistry. In Tables 2 and 3 the error percentage of the monomers with the wrong stereochemistry after applying for the first time the force field in runEXV is reported for all studied systems. We would like to point out that the trained model mimics in a certain way a real situation during PLA synthesis, during which full control of the stereochemistry is very challenging.^50^ Therefore, this model would be a valuable tool for the production of a variety of PLA polymers, whose composition would resemble that achieved under experimental conditions. However, since our objective is to obtain all-atom configurations with the exact stereochemistry in order to be able to quantitatively estimate the deviations from the target structures, we checked if the stereochemistry of each monomer corresponds to the desired sequence. If the stereochemistry of the monomer does not correspond to the one listed in the requested sequence, then the positions of the H_5_ atom and the methyl group are switched by a reflection matrix. Our open-access codes for both “checking” and “correcting” the stereochemistry are publicly accessible.^51^ The above procedure might create a few overlaps between atoms; thus, we perform an additional energy minimization step followed by another short run, as illustrated in Figure 1. Finally, the data for the analysis were collected from short MD simulations of a few (here 10) ns, labeled as runEQ, in which all monomers had the correct stereochemistry. In runEQ, the temperature was 500 K, and the LINCS algorithm was employed to constrain the bonds.^52^ Note that the same sequence of short runs, corrections, and energy minimization procedures was performed for all systems. Also, it is important to stress that the proposed sequence leads to a successful backmapping procedure, i.e., procedure which results in a desired atomistic structure, because the percentage of the “wrong” monomers in the predicted configuration is in most cases rather low (see Tables 2 and 3) and hence, the overlaps created by the application of the reflection matrix can be easily eliminated by the energy minimization procedure.

**Table 2:** Number and Percentage of Monomers with a Wrong Stereochemistry after Applying the Excluded Volume to the Predicted Structures of Systems of Groups A and B

group	A			B		
system	PLLA100	PDLA100	Copo100	PLLA30	PDLA30	Copo30
number/%	12/0.17	0/0	17/0.24	231/11	208/9.1	163/7.8

**Table 3:** Number and Percentage of Monomers with a Wrong Stereochemistry after Applying the Excluded Volume to the Predicted Structures of Copolymers Described in the section Chemical Transferability

system	Copo100RAND	Copo100HOM
number/%	13/0.18	1679/23.9

### 5.2 Validation of the Predicted Structures for the PLA Data Set A

To validate the predictive power of the backmapping algorithm, we perform an analysis that aims to detect the intramolecular (intra- and intermonomeric) and intermolecular deviations of the predicted structure from the target one. We consider as the target systems the systems obtained by the extensive atomistic MD simulations listed in Table 1.

**Figure 7:**
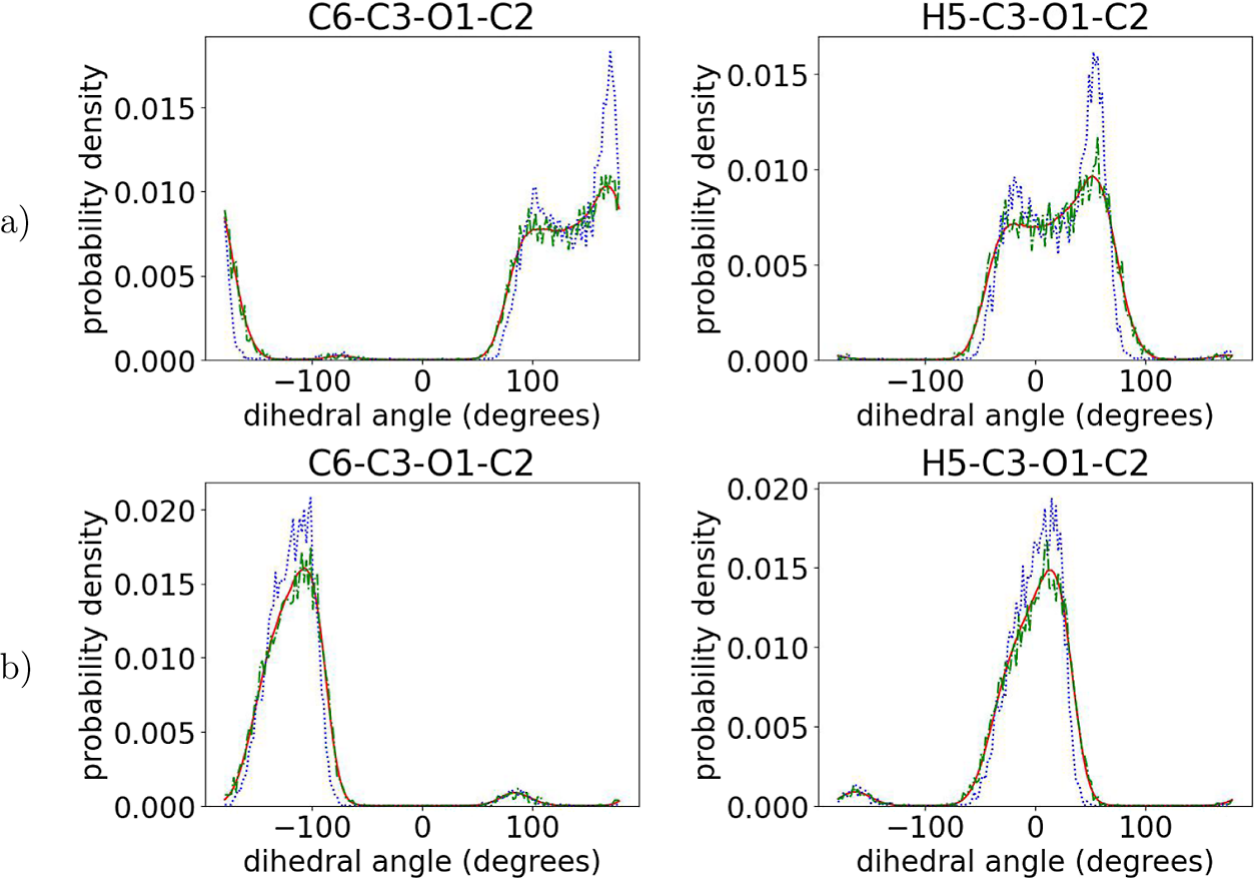
Comparison of the dihedral angles connecting two consequent monomers among target (red solid line) and initial predictions (blue dotted line) and the output of runEQ (green dash-dotted line) for 100-mer (a) PLLA and (b) PDLA configurations. For atom notation, see Figure 3.

**Figure 8:**
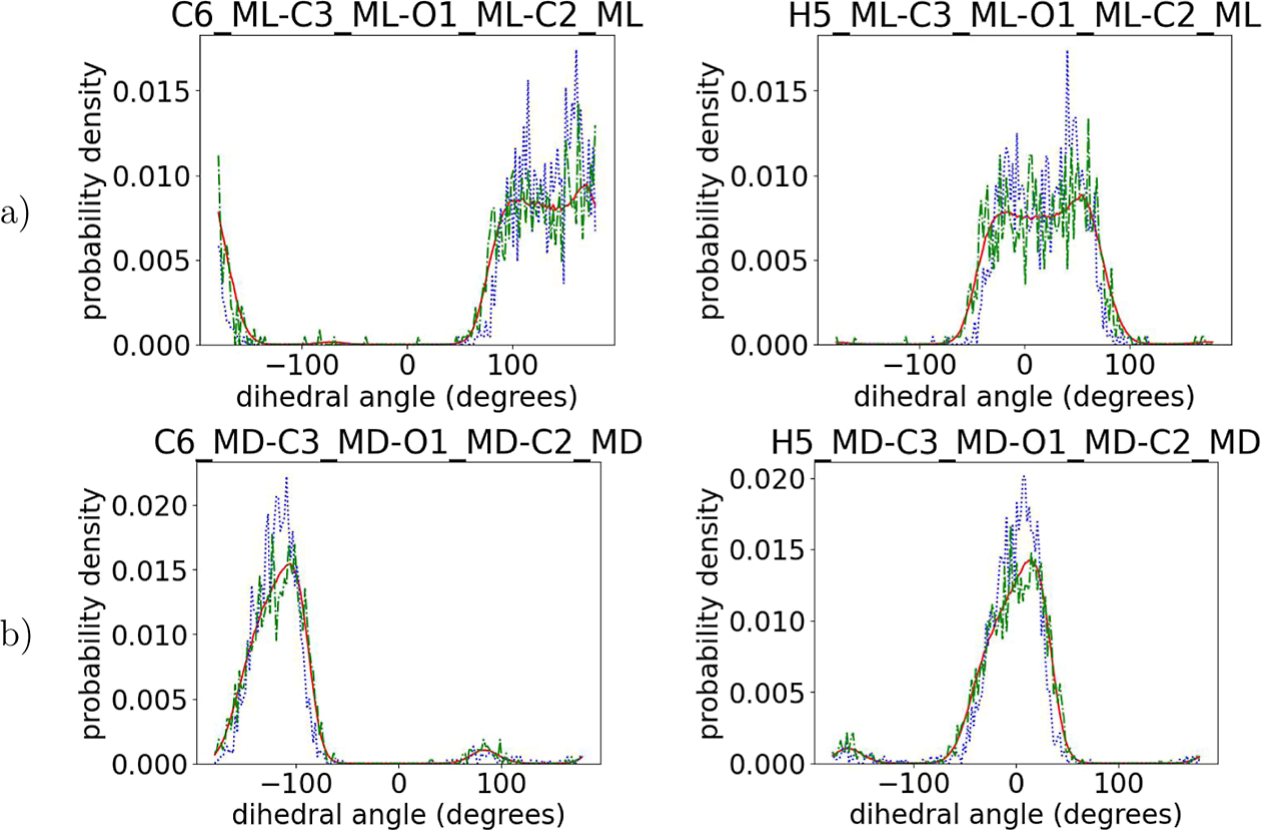
Comparison of intermonomeric dihedral angles among target (red solid line) and initial predictions (blue dotted line) and the output of runEQ (green dash-dotted line) for a 100-mer PLA copolymer configuration for (a) l monomers and (b) d monomers.

**Figure 9:**
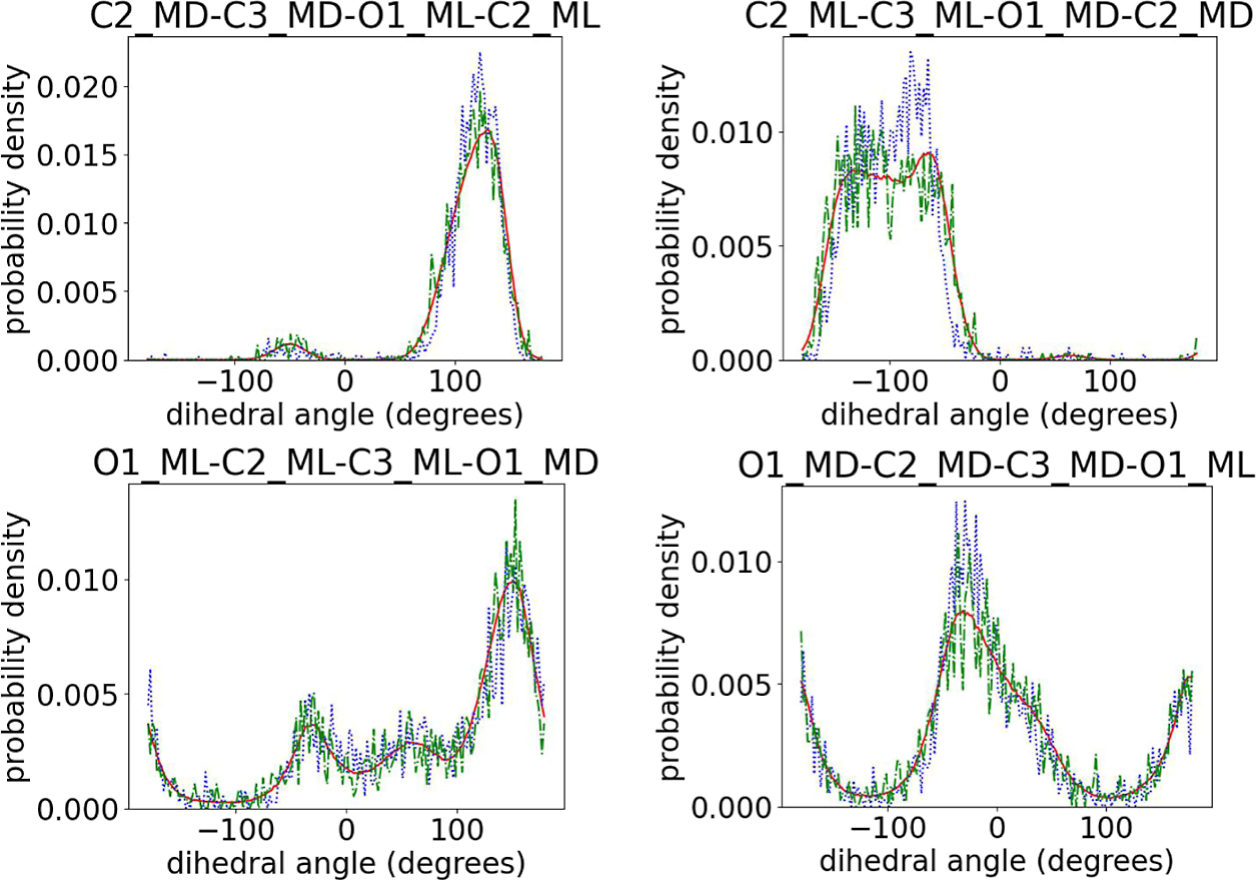
Comparison of intermonomeric dihedral angles among target (red solid line) and initial predictions (blue dotted line) and the output of runEQ (green dash-dotted line) for a 100-mer PLA copolymer configuration for the connection of l and d monomers. The atoms belonging to l monomers are labeled with “ML” and those belonging to d monomers are labeled with “MD”.

To uncover local intramolecular deviations between the back-mapped atomistic configurations and the target ones obtained from the production runs, we calculated the bonds, angles, and dihedral angle distributions along all PLA chains. Distributions of bonds and angles of the backmapped atomistic structures are in excellent agreement with the target ones, so in the discussion below we focus on the dihedral angles, which also strongly affect the local conformations of the PLA chains. In Figures S2–S5 in the Supporting Information, we show a typical comparison of intramonomeric dihedral angle distributions between target, initial prediction before applying the force field, and the output of runEQ for 100-mer PDLA, PLLA, and PDLLA copolymer configurations (denoted as Group A in Table 1). The results show only minor deviations of the initial predicted structures, compared to the target ones, which fully disappear after the short 10 ns MD runs (runEQ). Note that, in contrast to some Monte Carlo-based methods used in the past which mostly focus on capturing well the intramonomeric structure,^16,18^ the presented ML algorithm captures perfectly the intermonomeric dihedrals as well, as shown in Figures 7–9 for the dihedrals containing the chiral center and its ligands and in Figures S6–S9 in the Supporting Information for the remaining backbone atoms. More specifically, from the data shown in Figures 7–9, we observe that the positions of the peaks in the distributions obtained from the initial prediction match perfectly the target ones, indicating an accurate sampling of the desired arrangement of the chiral atoms with respect to the backbone. A minor deviation in the intensity of the peaks may imply a small difference between the relative proportions of these dihedrals in the predicted structures and those in the target atomistic data.

**Figure 10:**
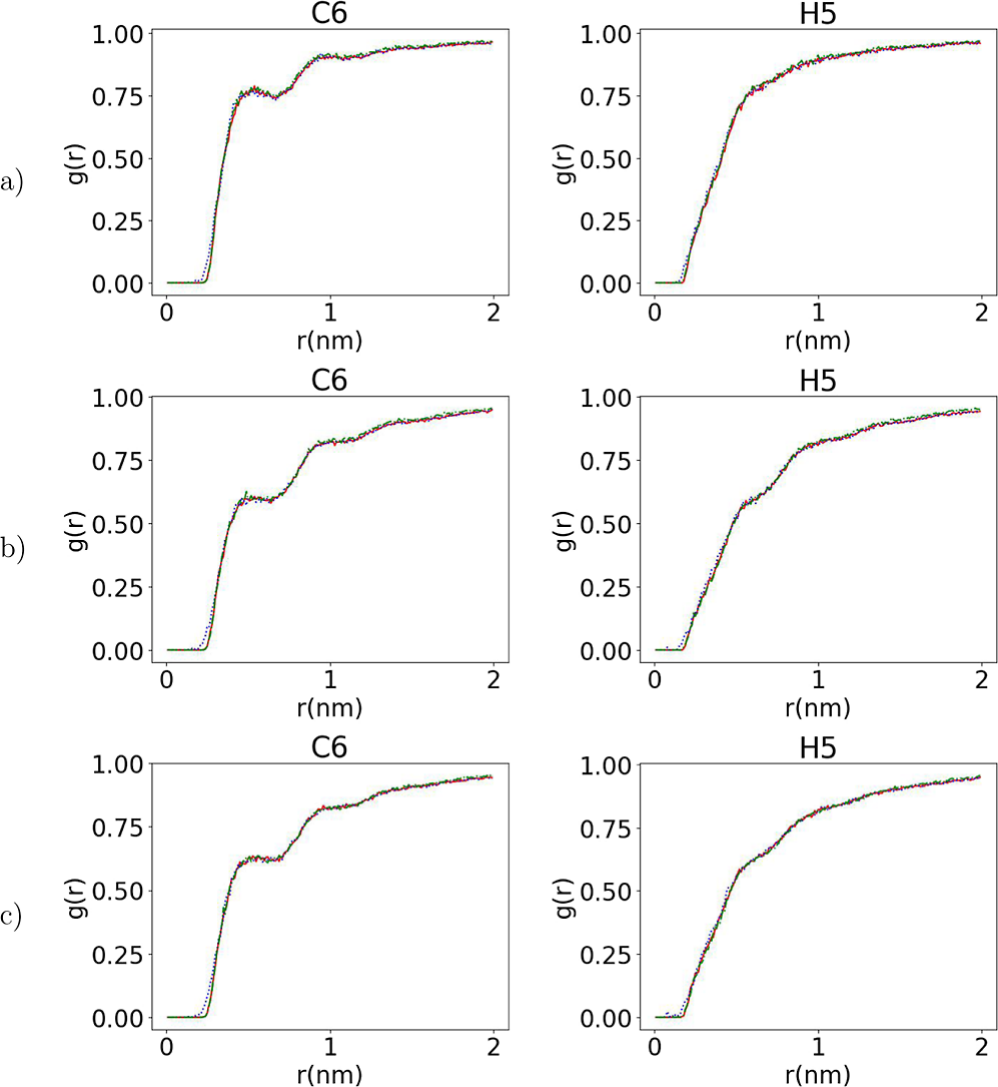
Comparison of intermolecular radial distribution functions around the given atoms among target (red solid line) and initial predictions (blue dotted line) and the output of runEQ (green dash-dotted line) for a 100-mer (a) PLLA, (b) PDLA, and (c) copolymer configuration.

The packing of the atoms in the system can be further examined in detail by calculating the radial distribution functions, *g*(*r*), for specific atoms. The intramolecular *g*(*r*)s, shown in Figures S10–S12 in the Supporting Information, in all three predicted cases match very well the target distributions of the systems from the Group A. We should note that due to the low percentage of monomers with the “wrong” stereochemistry in the predicted structures (see Table 2) and a very good prediction of the dihedral angles, this agreement is not surprising. Similar to the case of the dihedral distributions, the agreement gets even better after runEQ. On the other side, we recall that the neural network is based on a single-chain prediction, and therefore, it is not to be expected to predict exactly the intermolecular packing. Despite this limitation, it manages to capture extremely well the intermolecular *g*(*r*) distributions and thus the local packing around the atoms directly connected to the chiral center, as shown in Figure 10 but also the correlations between other atoms, which are presented in Figures S13–S15 in the Supporting Information. As a consequence of a well-reproduced packing, the densities ρ calculated from the runEQ match the target densities, namely ρ(PLLA100,target) = 1114 ± 3 kg/m^3^, ρ(PLLA100,runEQ) = 1114 ± 3 kg/m^3^ and ρ(PDLA100,target) = 1125 ± 3 kg/m^3^, ρ(PDLA100,runEQ) = 1125 ± 3 kg/m^3^, and ρ(Copo100,target) = 1120 ± 3 kg/m^3^, ρ(Copo100,runEQ) = 1119 ± 2 kg/m^3^.

Another consequence related to the well-predicted local packing would be a good recreation of the mutual placement of the donor and acceptor groups forming hydrogen bonds. However, in contrast to proteins, PLA polymers contain only two donor groups at the chain extremes in the form of a hydroxyl group. Therefore, we do not report this quantity, as due to the relatively high temperature and consequent high mobility of the terminal monomers, a one-to-one match of the geometrical alignment of the atoms forming a hydrogen bond is beyond the scope of our backmapping model. Nevertheless, the training model preserves the average number of hydrogen bonds per chain found in the target systems (data not shown here).

In highly dense systems, e.g., in the melt studied here, wrongly predicted initial configurations may cause numerical instabilities and/or lead to unphysical deformations, which lead to unfeasibly long times needed for equilibration. Therefore, the quality of the predicted configurations can be judged by the amount of time required to equilibrate the so-obtained system. It has been shown that the distribution of the internal distances is a good indicator of the polymer deformations at various length scales, represented by the monomeric separation distance, *n*, between two atoms in the backbone.^53^ In general, the longer the distance at which the deviation manifests, the longer the relaxation time that is needed for the equilibration of the predicted structure (see also the discussion in the section Atomistic and Coarse-Grained Poly(lactic acid) Models and Simulations). Note that for *n* equal to the number of atoms in the chain backbone, ⟨*R*_*n*_^2^⟩ = ⟨*R*_e_^2^⟩, where *R*_e_ is the end-to-end distance of the chain. The end-to-end vector relaxation at temperatures around 500 K for PLA chains occurs at time scales higher than 1 μs (data not shown here; see, e.g., ref (54) for a similar observation), and therefore, it is of utmost importance to predict structures free of significant deformations at length scales of the order of the end-to-end distance.

**Figure 11:**
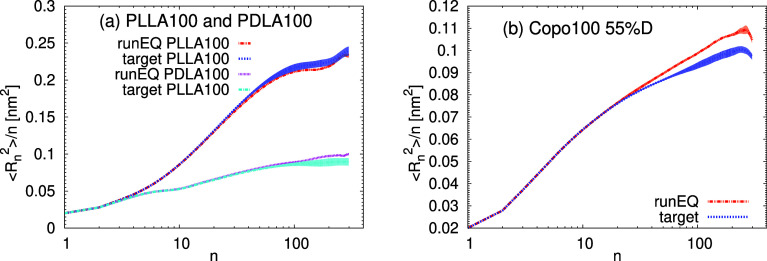
Internal distances ⟨*R*_*n*_^2^⟩/*n* as a function of the separation between the backbone atoms, *n*, for (a) PLLA100, PDLA100, and (b) Copo100 systems. The data for the predicted structures were averaged over the runEQ (10 ns). The shaded area represents the confidence interval.

In Figure 11 we show the distribution of the internal distances calculated from the runEQ run. The confidence interval represents the standard deviation of the calculated quantity, estimated from several (here four) blocks by the block average method, which is widely used in molecular dynamics simulations.^55^ At small *n*, the functions obtained for the predicted structures show a perfect match with the target one. At high values of *n*, the agreement for the PLLA100 and PDLA100 systems is very good within the obtained accuracy. The agreement between the predicted and the target data is also acceptable for the copolymer system, which shows some minor deviations at intermediate length scales. As the backmapping procedure is applied to the CG configuration obtained by coarse-graining of a target atomistic structure, the predicted system should also have the same distribution of the end-to-end distances as the target system. This means that the slight stretching of the predicted chains observed at longer *n* is a result of the excluded volume interactions applied during runEXV and runEQ. However, since a very small time step was used during the runEXV backmapping runs (i.e., a factor of 10 smaller than the commonly used time step), the excluded volume interactions were implemented gradually, and therefore, we do not observe any critical deformations.

Note that the time evolution of the end-to-end distance as well as of the radius of gyration have been monitored in the past as one of the indicators of a well-equilibrated PLA structure.^54^ Even though the full analysis of the structural and dynamical properties of PLA systems is beyond the scope of this paper, we stress that the radii of gyration of the predicted structures of PLLA100 and PDLA100 obtained from the runEQ are identical, within the error bars, to those obtained from the production runs (i.e., to the target systems). The radius of gyration of the predicted Copo100 is 3.6% higher than that of the target system. This small deviation is in line with our observations made with respect to Figure 11.

In summary, we showed that the predicted atomistic configurations of homopolymer and copolymer PLA chains exhibit minor structural differences in comparison to the target systems, which fully vanish after very short equilibration MD runs of only 10 ns. For the 100-mer PLA systems, this 10 ns simulation lasted 5 h on 100 processors on an IBM NeXtScale nx360 M4 system with Ivy Bridge—Intel Xeon E5-2680v2 processors.

### 5.3 Chemical Transferability

In this section, we investigate the transferability of the DL-based models for PLA 100-mer copolymers, which have the same chemical composition as the target copolymer (i.e., 55% of the d content) but were prepared by a different backmapping strategy. Namely, we examined 2 cases, labeled as Copo100RAND and Copo100HOM in Table 3. In both cases, the target copolymer system from group A was used to produce the initial CG configuration necessary for the backmapping procedure. In this way, we make sure that the resultant distribution of the end-to-end distances resembles the one for the target system.

Copo100HOM has the same sequence of stereoisomers per chain as the target system, but the neural network used for the backmapping procedure was trained solely on the homopolymer data from group A. Note that the local, intramonomeric dihedrals in copolymer and homopolymer are identical (compare the distributions in Figures S2 and S3 with S4 and S5 in the Supporting Information); therefore, the ML-based algorithm is expected to reproduce well the corresponding distributions. On the other side, as the training set does not contain the information about the connection between the l- and d-monomers (i.e., about the mixed dihedrals), the prediction of these dihedrals is expected to be poor.

Copo100RAND represents a different synthetic path for the production of a random copolymer with a 55% d content. Namely, each chain in the system has the same d-stereoisomer content, but the sequences of the l and d monomers among chains differ. Since the initial CG configuration used for the backmapping procedure contains the same sequence per chain (Copo100 system), during the backmapping procedure of Copo100RAND the atoms of d monomers may be inserted in the CG bead corresponding originally to the l monomer and the other way around. The different sequence of l and d monomers along the chain may lead to some local deviations in packing with respect to the target copolymer system but having in mind that we probe average properties and the number of monomers per system is relatively high, we assume that the Copo100 from Group A (i.e., target) and the newly created Copo100RAND will eventually exhibit the same structural properties. In the case of the Copo100RAND prediction, the atomistic data for all systems from Group A were used for the training set; therefore, a better prediction for the mixed dihedrals is expected in comparison to the prediction of Copo100HOM.

**Figure 12:**
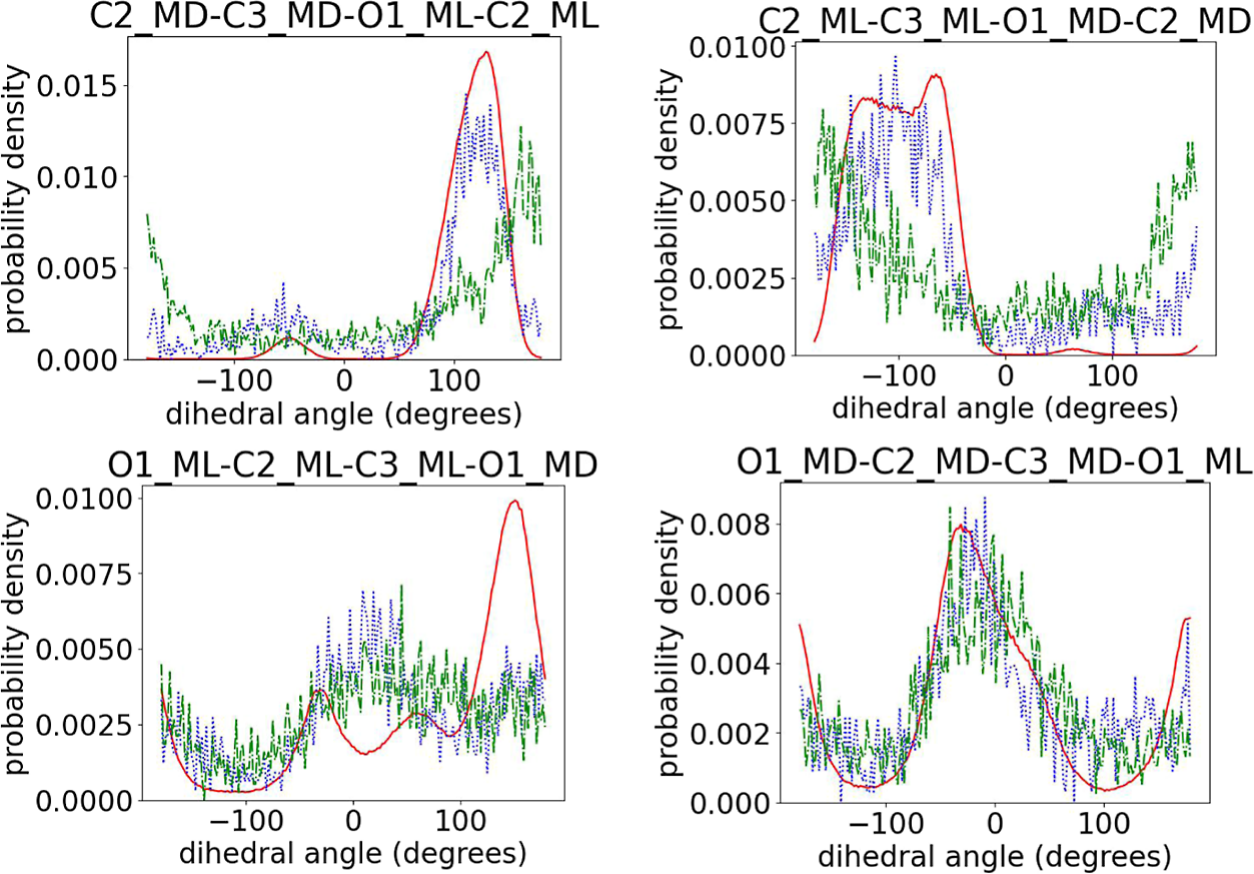
Comparison of intermonomeric dihedral angles among target (red solid line) and initial predictions for Copo100RAND (blue dotted line) and Copo100HOM (green dash-dotted line). The atoms belonging to l monomers are labeled with “ML”, and those belonging to d monomers are labeled with “MD”.

The predictions of the intramonomeric dihedral distribution functions (Figures S16 and S17 in the Supporting Information) and of the intermonomeric distributions for the same type of monomer (Figures S18 and S19 in the Supporting Information) are reasonable for both types of copolymers. In general, the prediction is better for Copo100RAND and for monomers containing d chiral centers. Note that this observation is related to the fact that in Copo100HOM 62% of the “wrongly” predicted monomers are monomers, which would correspond to l monomers in the desired sequence. In other words, the presented algorithm struggles more to properly place the H_5_ atom in the l chiral centers of Copo100HOM than in the d ones. Concerning the mixed dihedrals plotted in Figure 12, we observed the expected behavior. More specifically, the ML-based algorithm performs better in the case of Copo100RAND.

Similar to Group A, after the runEQ all dihedral distributions in Copo100HOM and Copo100RAND match perfectly with the target distributions (data not shown here). The higher percentages of the wrong monomers found for Copo100HOM (reported in Table 3) in comparison to the predictions of systems from Group A are reflected in the intramolecular *g*(*r*) distribution for the backbone atoms plotted in Figure S20 in the Supporting Information. Namely, the misplacement of the H_5_ atom in the monomers with the wrong stereochemistry causes alignment of the C_3_–H_5_ bond with the main backbone, which leads to overlaps with the backbone atoms, visible as nonzero values of the intramolecular *g*(*r*) at very small *r* in Figure S20 in the Supporting Information.

Consequently, as the error in the H_5_ insertion occurs in the vicinity of the main backbone and thus close to the center of mass of the CG unit, the intermolecular *g*(*r*)s for the predicted structures remain unaffected and are in very good agreement with the target distributions (see Figure S21 in the Supporting Information). All intra- and intermolecular *g*(*r*)s converge to the target ones after a 10 ns runEQ (data not shown here). Consequently, the densities calculated from the runEQ are in very good agreement with the target densities, namely, ρ(Copo100HOM,runEQ) = 1121 ± 3 kg/m^3^ and ρ(Copo100RAND,runEQ) = 1119 ± 3 kg/m^3^.

**Figure 13:**
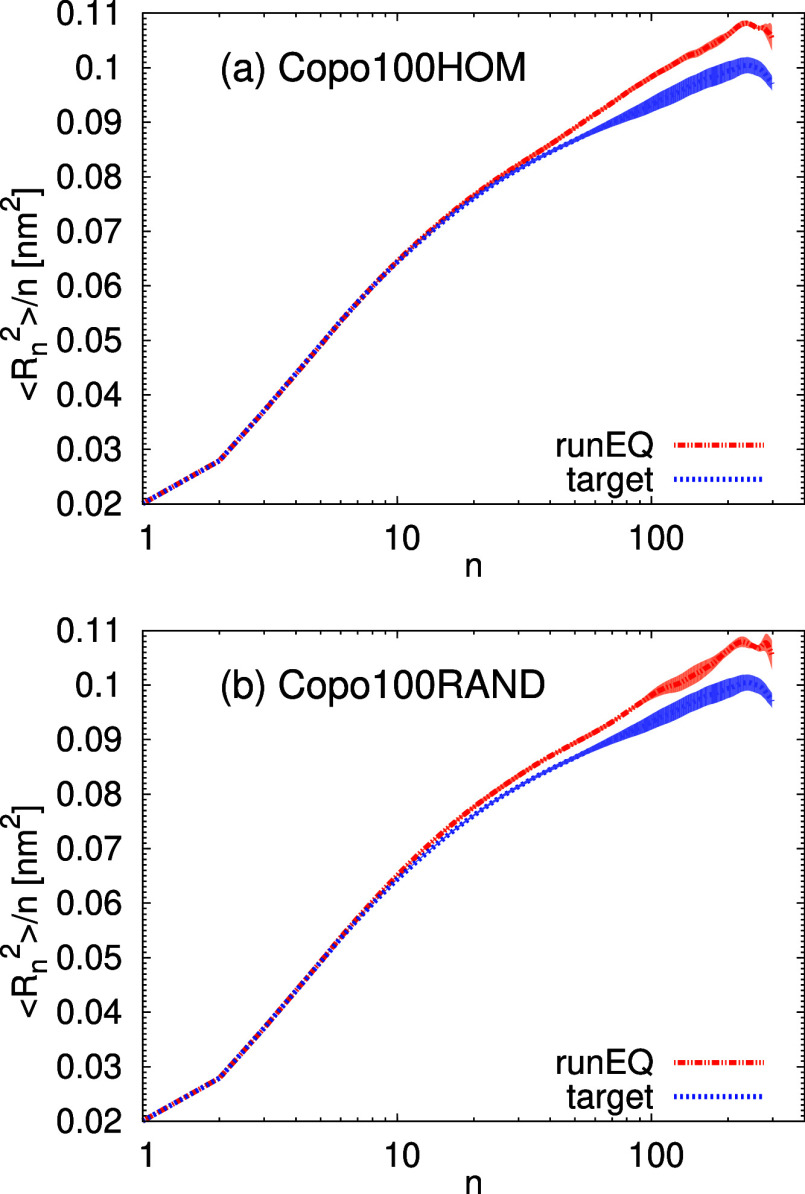
Internal distances ⟨*R*_*n*_^2^⟩/*n* as a function of the separation between the backbone atoms, *n*. The data for the predicted structures were averaged over the runEQ (10 ns). The shaded area represents the confidence interval. The system labeled as Copo100RAND contains chains with a different random sequence of chiral monomers than the copolymer in Group A, and the system labeled as Copo100HOM has the composition identical to Copo100 from Group A, but its training set consisted only of the homopolymer data.

The distributions of the internal distances shown in Figure 13 resemble the case of Copo100 from Group A. More specifically, a minor stretching at longer distances is observed, with a slightly better agreement with the target function at intermediate distances than that in Figure 11b. This observation is also in line with the average value of the radius of gyration obtained from the runEQ, which is 2.7% higher than the target one. Overall, the validation of the chemical transferability of the presented algorithm led to very satisfactory results.

In the case of the Copo100HOM system, the algorithm could be used for producing copolymer configurations without having the target atomistic copolymer as the training set. However, it must be stressed that an initial CG configuration with the end-to-end distribution corresponding to the desired d content is a must because the d content is closely related to the stiffness of the chains.^56^ Note that an identical condition was applied in the preparation method of the entangled coarse-grained models with varying stiffness.^53^

In the case of the Copo100RAND, we showed that the algorithm is capable of producing copolymers made by different synthetic routes. This feature is very useful for studies imitating the experimental conditions, allowing for the recreation of multiple realizations of the same experiment. In addition, the results indicate that the atoms of the d monomer can be reinserted into CG units of l monomers and vice versa without significant deformations in the system. This fact opens the door for the backmapping of generic CG models with only one type of CG unit.

### 5.4 Transferability Across Molecular Length

As a last part of our analysis, we investigate the accuracy of the derived models concerning their transferability across different molecular weights (chain lengths). We recall that the models were trained using 100-mer PLA systems. As an illustrative example, we apply the trained models to PLA homopolymer and copolymer systems consisting of 30 monomeric units (see Table 1). For the 30-mer homopolymer systems, we make the predictions using the models developed with their corresponding 100-mer homopolymers, while for the 30-mer copolymer system, due to the different d content compared to the 100-mer copolymer, we utilize a model trained with all three 100-mer systems.

The dihedral angle distributions for the homopolymers match perfectly the target functions (see Figures S22 and S23 in the Supporting Information). As a consequence of the high number of predicted monomers with the wrong stereochemistry (see Table 2), some overlaps are present in the systems, visible in the intramolecular *g*(*r*) in Figures S27 and S28 in the Supporting Information. This might be caused by the different structure (number of atoms per sample) of the samples given as input to the neural network compared to the ones of the 100-mer systems utilized for the training process. Nevertheless, all distributions converged to the target ones after the runEQ (see also Figures S29 and S30 in the Supporting Information).

**Figure 14:**
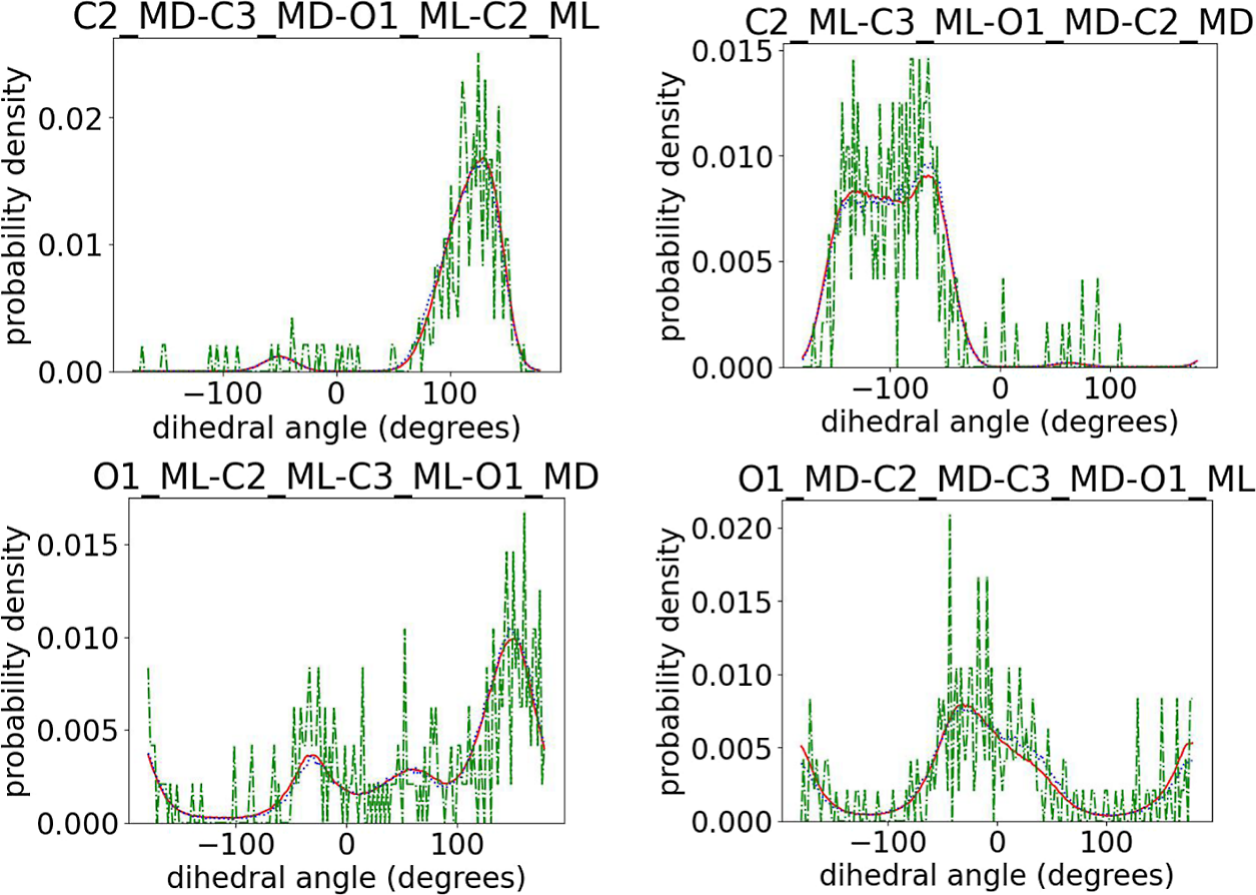
Comparison of intermonomeric dihedral angles among 30-mer (blue dotted line) and 100-mer (red solid line) target systems and the initial prediction for the Copo30 system (green dash-dotted line). The atoms belonging to l monomers are labeled with “ML”, and those belonging to d monomers are labeled with “MD”.

Concerning the copolymer case, as the intermonomeric dihedral distributions for the target 100-mer and the 30-mer copolymer are indistinguishable within the given accuracy despite having different d contents, the algorithm performs very well in predicting those distributions (see Figure 14). The remaining dihedral distributions as well as *g*(*r*)s plotted in the Supporting Information (Figures S24–S26, S31 and S32) agree very well with the target data. In addition, the radii of gyration of all 3 systems as well as their densities ρ calculated from runEQ fall in the confidence interval of the target values, i.e., ρ(PLLA30,target) = 1112 ± 5 kg/m^3^, ρ(PLLA30,runEQ) = 1117 ± 4 kg/m^3^ and ρ(PDLA30,target) = 1122 ± 5 kg/m^3^, ρ(PDLA30,runEQ) = 1123 ± 4 kg/m^3^ and ρ(Copo30,target) = 1112 ± 5 kg/m^3^, ρ(Copo30,runEQ) = 1115 ± 4 kg/m^3^.

**Figure 15:**
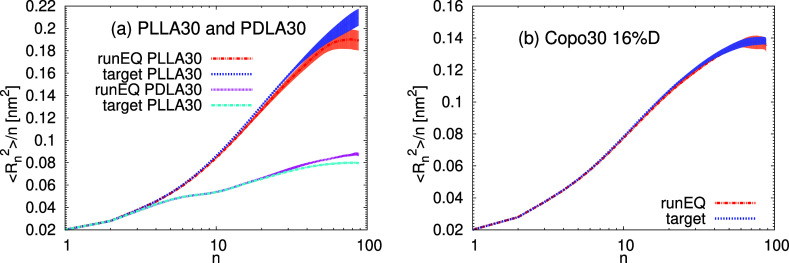
Internal distances ⟨*R*_*n*_^2^⟩/*n* for (a) PLLA30, PDLA30, and (b) Copo30 systems. The data for the predicted structure were averaged over the runEQ (10 ns). The shaded area represents the confidence interval.

Furthermore, in Figure 15 we show a comparison for the inner distance distribution. Overall, for most of the distributions, we have similar behavior between the initial predictions of the 30-mer and the 100-mer systems, which demonstrates the robustness of the trained models across different molecular lengths. This feature of the presented algorithm may serve for the preparation of polymer chains with industrially relevant systems of high molecular weights as well as of polydisperse systems, which consist of chains with varying molecular weights.

## 6 Conclusions

We presented a new computational methodology for reinserting atomic detail in coarse-grained configurations of biodegradable macromolecules with chiral centers, based on physics-informed U-net CNN models. The proposed backmapping procedure, which combines the ML algorithm with short MD runs and an algorithm for validating the stereochemistry, is versatile and quick and was able to successfully reconstruct all-atom configurations of multiple stereoisomers of poly(lactic acid). The current approach requires only local information; therefore, the trained model can be applied to molecular systems of arbitrary chain length. Furthermore, the proposed method avoids tedious and labor-intensive bookkeeping of molecular details during the reconstruction by separating configurational information from molecular topology and force-field.

Therefore, we believe that the presented approach can be extended to any type of polymer with chiral centers and a broad range of molecular weights. In addition, as the methodology is not limited to chiral molecules and proved to be very efficient in reinserting atoms with connectivity up to 4, it can be generally applied for the backmapping of any all-atom as well as united-atom representation of polymer-based materials.

The approach can be particularly useful for a quickly growing community of scientists dealing with biobased and/or biodegradable polymers, as for these complex systems it is essential to start the simulations with an initial configuration, which resembles the equilibrated state, as the equilibration times exceed, in many cases, those feasible by the current computational resources. Due to its very precise prediction of atomistic structures, which closely resemble fully equilibrated structures, the current methodology may significantly reduce computational demands.

Concerning the future challenges, it is certainly of high interest to investigate the versatility of the algorithm for CG models that are developed via a top-down approach, i.e., an approach where the CG structures are not produced by a systematic coarse-graining of the atomistic structures but more generic bead–spring-like models. Another topic concerns the application of the proposed methodology to industrially relevant, high-molecular-weight PLA systems or other biodegradable polymers.

## Data Availability

The code for the development of the Deep Learning model and the computation of the dihedral angle distributions reported in the manuscript is uploaded in GitHub (see ref (57)). Moreover, the codes developed and used for both “checking” and “correcting” the stereochemistry are publicly accessible in GitHub, together with the scripts for the calculation of the inner distances and radial distribution functions (see ref (51)). We note that due to the magnitude of the trajectory files, only a small sample of the Copo100 system (100 frames) is available in GitHub (see ref (57)); nevertheless, the rest of the data that support the findings of this study are available from the corresponding author upon request.

## Supporting Information Available

Additional information on all PLA systems considered in this work (i.e., PLLA100, PDLA100, Copo100, Copo100RAND, Copo100HOM, PLLA30, PDLA30, and Copo30) related to intra- and intermonomeric dihedral angle distributions and intra- and intermolecular radial distribution functions specific for a number of different particles; and comparison of the model used for future runs and a model where we only penalize the bond vectors (PDF)

## References

[ref1] Doi M.; Edwards S. F. The Theory of Polymer Dynamics; Oxford University Press: USA, 1986.

[ref2] Theodorou D.. In Computer Simulations in Condensed Matter Systems: From Materials to Chemical Biology; Ferrario M., Ciccotti G., Binder K., Eds.; Springer Berlin Heidelberg: Berlin, Heidelberg, 2006; Vol. 2, pp 419–448.

[ref3] Noid W. G. Perspective: Coarse-Grained Models for Biomolecular Systems. J. Chem. Phys. 2013, 139, 090901 10.1063/1.4818908. 24028092

[ref4] Marrink S. J.; Risselada H. J.; Yefimov S.; Tieleman D. P.; de Vries A. H. The MARTINI Force Field: Coarse Grained Model for Biomolecular Simulations. J. Phys. Chem. B 2007, 111, 7812–7824. 10.1021/jp071097f. 17569554

[ref5] Marrink S. J.; Monticelli L.; Melo M. N.; Alessandri R.; Tieleman D. P.; Souza P. C. T. Two Decades of MARTINI: Better Beads, Broader Scope. Wiley Interdiscip. Rev.: Comput. Mol. Sci. 2023, 13, e1620 10.1002/wcms.1620.

[ref6] Harmandaris V. A.; Kremer K. Predicting Polymer Dynamics at Multiple Length and Time Scales. Soft Matter 2009, 5, 3920–3926. 10.1039/b905361a.

[ref7] Kremer K.; Müller-Plathe F. Multiscale simulation in polymer science. Mol. Simul. 2002, 28, 729–750. 10.1080/0892702021000002458.

[ref8] Harmandaris V. A.; Adhikari N. P.; van der Vegt N. F. A.; Kremer K. Hierarchical Modeling of Polystyrene: From Atomistic to Coarse-Grained Simulations. Macromolecules 2006, 39, 6708–6719. 10.1021/ma0606399.

[ref9] Qian H.-J.; Carbone P.; Chen X.; Karimi-Varzaneh H. A.; Liew C. C.; Müller-Plathe F. Temperature-Transferable Coarse-Grained Potentials for Ethylbenzene, Polystyrene, and Their Mixtures. Macromolecules 2008, 41, 9919–9929. 10.1021/ma801910r.

[ref10] Horstemeyer M. F. Practical Aspects of Computational Chemistry: Methods, Concepts and Applications; Springer Netherlands: Dordrecht, 2010; pp 87–135.

[ref11] Peter C.; Kremer K. Multiscale Simulation of Soft Matter Systems – from the Atomistic to the Coarse-Grained Level and back. Soft Matter 2009, 5, 4357–4366. 10.1039/b912027k.

[ref12] Santangelo G.; Di Matteo A.; Müller-Plathe F.; Milano G. From Mesoscale Back to Atomistic Models: A Fast Reverse-Mapping Procedure for Vinyl Polymer Chains. J. Phys. Chem. B 2007, 111, 2765–2773. 10.1021/jp066212l. 17319712

[ref13] Rzepiela A. J.; Schäfer L. V.; Goga N.; Risselada H. J.; de Vries A. H.; Marrink S. J. Reconstruction of Atomistic Details from Coarse-Grained Structures. J. Comput. Chem. 2010, 31, 1333–1343. 10.1002/jcc.21415. 20087907

[ref14] Krajniak J.; Pandiyan S.; Nies E.; Samaey G. Generic Adaptive Resolution Method for Reverse Mapping of Polymers from Coarse-Grained to Atomistic Descriptions. J. Chem. Theory Comput. 2016, 12, 5549–5562. 10.1021/acs.jctc.6b00595. 27685340

[ref15] Zhang G.; Chazirakis A.; Harmandaris V. A.; Stuehn T.; Daoulas K. C.; Kremer K. Hierarchical Modelling of Polystyrene Melts: From Soft Blobs to Atomistic Resolution. Soft Matter 2019, 15, 289–302. 10.1039/C8SM01830H. 30543257

[ref16] Pandey Y. N.; Brayton A.; Burkhart C.; Papakonstantopoulos G. J.; Doxastakis M. Multiscale Modeling of Polyisoprene on Graphite. J. Chem. Phys. 2014, 140, 054908 10.1063/1.4863918. 24511980

[ref17] Spyriouni T.; Tzoumanekas C.; Theodorou D.; Müller-Plathe F.; Milano G. Coarse-Grained and Reverse-Mapped United-Atom Simulations of Long-Chain Atactic Polystyrene Melts: Structure, Thermodynamic Properties, Chain Conformation, and Entanglements. Macromolecules 2007, 40, 3876–3885. 10.1021/ma0700983.

[ref18] Ghanbari A.; Böhm M. C.; Müller-Plathe F. A Simple Reverse Mapping Procedure for Coarse-Grained Polymer Models with Rigid Side Groups. Macromolecules 2011, 44, 5520–5526. 10.1021/ma2005958.

[ref19] Wassenaar T. A.; Pluhackova K.; Böckmann R. A.; Marrink S. J.; Tieleman D. P. Going Backward: A Flexible Geometric Approach to Reverse Transformation from Coarse Grained to Atomistic Models. J. Chem. Theory Comput. 2014, 10, 676–690. 10.1021/ct400617g. 26580045

[ref20] Krajniak J.; Zhang Z.; Pandiyan S.; Nies E.; Samaey G. Reverse Mapping Method for Complex Polymer Systems. J. Comput. Chem. 2018, 39, 648–664. 10.1002/jcc.25129. 29214661

[ref21] Peng J.; Yuan C.; Ma R.; Zhang Z. Backmapping from Multiresolution Coarse-Grained Models to Atomic Structures of Large Biomolecules by Restrained Molecular Dynamics Simulations Using Bayesian Inference. J. Chem. Theory Comput. 2019, 15, 3344–3353. 10.1021/acs.jctc.9b00062. 30908042

[ref22] Lombardi L. E.; Martí M. A.; Capece L. CG2AA: Backmapping Protein Coarse-Grained Structures. Bioinformatics 2016, 32, 1235–1237. 10.1093/bioinformatics/btv740. 26677962

[ref23] Machado M. R.; Pantano S. SIRAH Tools: Mapping, Backmapping and Visualization of Coarse-Grained Models. Bioinformatics 2016, 32, 1568–1570. 10.1093/bioinformatics/btw020. 26773132

[ref24] Badaczewska-Dawid A. E.; Kolinski A.; Kmiecik S. Computational Reconstruction of Atomistic Protein Structures from Coarse-Grained Models. Comput. Struct. Biotechnol. J. 2020, 18, 162–176. 10.1016/j.csbj.2019.12.007. 31969975 PMC6961067

[ref25] Vickery O. N.; Stansfeld P. J. CG2AT2: An Enhanced Fragment-Based Approach for Serial Multi-Scale Molecular Dynamics Simulations. J. Chem. Theory Comput. 2021, 17, 6472–6482. 10.1021/acs.jctc.1c00295. 34492188 PMC8515810

[ref26] Ghanbari A.; Bohm M. C.; Muller-Plathe F. A Simple Reverse Mapping Procedure for Coarse-Grained Polymer Models with Rigid Side Groups. Macromolecules 2011, 44, 5520–5526. 10.1021/ma2005958.

[ref27] Kuo A.-T.; Miyazaki Y.; Jang C.; Miyajima T.; Urata S.; Nielsen S. O.; Okazaki S.; Shinoda W. Large-Scale Molecular Dynamics Simulation of Perfluorosulfonic Acid Membranes: Remapping Coarse-Grained to All-Atomistic Simulations. Polymer 2019, 181, 121766 10.1016/j.polymer.2019.121766.

[ref28] Li W.; Burkhart C.; Polińska P.; Harmandaris V.; Doxastakis M. Backmapping Coarse-Grained Macromolecules: An Efficient and Versatile Machine Learning Approach. J. Chem. Phys. 2020, 153, 041101 10.1063/5.0012320. 32752654

[ref29] Stieffenhofer M.; Wand M.; Bereau T. Adversarial Reverse Mapping of Equilibrated Condensed-Phase Molecular Structures. Mach. Learn.: Sci. Technol. 2020, 1, 045014 10.1088/2632-2153/abb6d4.

[ref30] Wang W.; Gómez-Bombarelli R. Coarse-Graining Auto-Encoders for Molecular Dynamics. npj Comput. Mater. 2019, 5, 125 10.1038/s41524-019-0261-5.

[ref31] Heo L.; Feig M. One Bead per Residue can Describe All-Atom Protein Structures. Structure 2024, 32, 97–111.e6. 10.1016/j.str.2023.10.013. 38000367 PMC10872525

[ref32] An Y.; Deshmukh S. A. Machine Learning Approach for Accurate Backmapping of Coarse-Grained Models to All-Atom Models. Chem. Commun. 2020, 56, 9312–9315. 10.1039/D0CC02651D. 32667366

[ref33] Samir A.; Ashour F.; Hakim A.; Bassyouni M. Recent Advances in Biodegradable Polymers for Sustainable Applications. npj Mater. Degrad. 2022, 6, 68 10.1038/s41529-022-00277-7.

[ref34] Jamshidian M.; Tehrany E. A.; Imran M.; Jacquot M.; Desobry S. Poly-Lactic Acid: Production, Applications, Nanocomposites, and Release Studies. Compr. Rev. Food Sci. Food Saf. 2010, 9, 552–571. 10.1111/j.1541-4337.2010.00126.x. 33467829

[ref35] Naser A. Z.; Deiab I.; Defersha F.; Yang S. Expanding Poly(lactic acid) (PLA) and Polyhydroxyalkanoates (PHAs) Applications: A Review on Modifications and Effects. Polymers 2021, 13, 4271 10.3390/polym13234271. 34883773 PMC8659978

[ref36] Guseva D. V.; Glagolev M. K.; Lazutin A. A.; Vasilevskaya V. V. Revealing Structural and Physical Properties of Polylactide: What Simulation Can Do Beyond the Experimental Methods. Polym. Rev. 2023, 64, 80–118. 10.1080/15583724.2023.2174136.

[ref37] Glagolev M. K.; Vasilevskaya V. V. Coarse-grained Simulation of Molecular Ordering in Polylactic Blends under Uniaxial Strain. Polymer 2020, 190, 122232 10.1016/j.polymer.2020.122232.

[ref38] Glagolev M.; Glova A.; Mezhenskaia D.; Falkovich S.; Larin S.; Vasilevskaya V.; Lyulin S. Coarse-Grained A-graft-B Model of Poly(lactic acid) for Molecular Dynamics Simulations. J. Polym. Sci., Part B: Polym. Phys. 2018, 56, 604–612. 10.1002/polb.24567.

[ref39] Prasitnok K. A Coarse-Grained Model for Polylactide: Glass Transition Temperature and Conformational Properties. J. Polym. Res. 2016, 23, 139 10.1007/s10965-016-1037-y.

[ref40] Glagolev M. K.; Vasilevskaya V. V. Reverse Mapping Algorithm for Multi-scale Numerical Simulation of Polylactic Acid. Supercomput. Front. Innov. 2018, 5, 103–106. 10.14529/jsfi180319.

[ref41] Guseva D. V.; Lazutin A. A.; Vasilevskaya V. V. Atomistic Simulation of Poly (lactic acid) of Different Regioregularity. Polymer 2021, 221, 123577 10.1016/j.polymer.2021.123577.

[ref42] Christofi E.; Chazirakis A.; Chrysostomou C.; Nicolaou M.; Li W.; Doxastakis M.; Harmandaris V. Deep Convolutional Neural Networks for Generating Atomistic Configurations of Multi-Component Macromolecules from Coarse-Grained Models. J. Chem. Phys. 2022, 157, 184903 10.1063/5.0110322. 36379782

[ref43] Kalligiannaki E.; Harmandaris V. A.; Katsoulakis M. A.; Plecháč P. The Geometry of Generalized Force Matching and Related Information Metrics in Coarse-Graining of Molecular Systems. J. Chem. Phys. 2015, 143, 084105 10.1063/1.4928857. 26328816

[ref44] Abraham M.; Murtola T.; Schulz R.; Páll S.; Smith J.; Hess B.; Lindahl E. GROMACS: High Performance Molecular Simulations through Multi-Level Parallelism from Laptops to Supercomputers. SoftwareX 2015, 1–2, 19–25. 10.1016/j.softx.2015.06.001.

[ref45] McAliley J. H.; Bruce D. A. Development of Force Field Parameters for Molecular Simulation of Polylactide. J. Chem. Theory Comput. 2011, 7, 3756–3767. 10.1021/ct200251x. 22180734 PMC3237685

[ref46] Glova A. D.; Falkovich S. G.; Larin S. V.; Mezhenskaia D. A.; Lukasheva N. V.; Nazarychev V. M.; Tolmachev D. A.; Mercurieva A. A.; Kenny J. M.; Lyulin S. V. Poly(lactic acid)-based Nanocomposites Filled with Cellulose Nanocrystals with Modified Surface: All-Atom Molecular Dynamics Simulations. Polym. Int. 2016, 65, 892–898. 10.1002/pi.5102.

[ref47] Frenkel D.; Smit B. Understanding Molecular Simulation, From Algorithms to Applications (Computational Science), 2nd ed.; Academic Press, 2001.

[ref48] Abadi M.; TensorFlow: Large-Scale Machine Learning on Heterogeneous Systems, 2015. https://www.tensorflow.org/.

[ref49] Chen L. J.; Qian H. J.; Lu Z. Y.; Li Z. S.; Sun C. C. An Automatic Coarse-Graining and Fine-Graining Simulation Method: Application on Polyethylene. J. Phys. Chem. B 2006, 110, 24093–24100. 10.1021/jp0644558. 17125381

[ref50] Michell R. M.; Ladelta V.; Da Silva E.; Müller A. J.; Hadjichristidis N. Poly(lactic Acid) Stereocomplexes Based Molecular Architectures: Synthesis and Crystallization. Prog. Polym. Sci. 2023, 146, 101742 10.1016/j.progpolymsci.2023.101742.

[ref51] Bačová P. PLA Analysis Tools, 2023. https://github.com/pbacova/PLA_analysis_tools.git.

[ref52] Hess B.; Bekker H.; Berendsen H. J. C.; Fraaije J. G. E. M. LINCS: A Linear Constraint Solver for Molecular Simulations. J. Comput. Chem. 1997, 18, 1463–1472. 10.1002/(SICI)1096-987X(199709)18:12<1463::AID-JCC4>3.0.CO;2-H.

[ref53] Auhl R.; Everaers R.; Grest G. S.; Kremer K.; Plimpton S. J. Equilibration of Long Chain Polymer Melts in Computer Simulations. J. Chem. Phys. 2003, 119, 12718–12728. 10.1063/1.1628670.

[ref54] Glova A. D.; Falkovich S. G.; Dmitrienko D. I.; Lyulin A. V.; Larin S. V.; Nazarychev V. M.; Karttunen M.; Lyulin S. V. Scale-Dependent Miscibility of Polylactide and Polyhydroxybutyrate: Molecular Dynamics Simulations. Macromolecules 2018, 51, 552–563. 10.1021/acs.macromol.7b01640.

[ref55] Rapaport D. C. The Art of Molecular Dynamics Simulation, 2nd ed.; Cambridge University Press, 2004.

[ref56] Sasanuma Y.; Touge D. Configurational Statistics of Poly(L-lactide) and Poly(DL-lactide) Chains. Polymer 2014, 55, 1901–1911. 10.1016/j.polymer.2014.01.059.

[ref57] Christofi E. PLA Backmapping, 2023 . https://github.com/SimEA-ERA/PLA-BackMap-CG.

