# Correction to “Physics-Informed Deep Learning Approach for Reintroducing Atomic Detail in Coarse-Grained Configurations of Multiple Poly(lactic acid) Stereoisomers”

**DOI:** 10.1021/acs.jcim.4c01407

**Published:** 2024-08-19

**Authors:** Eleftherios Christofi, Petra Bačová, Vagelis A. Harmandaris

Regarding our original paper, concerning the actual flexibility of the PDLA and consequently the values of the internal distances presented in Figures 11, 13, and 15, it is important to note that the same force field parameters have been used for both l- and d-monomers; i.e., these two structures differ only in the spatial position of the hydrogen in the monomer. More specifically, according to the force field published in ref 45 of the original manuscript the l- and d-forms of poly(lactic acid) should differ in the parameters for CMAP dihedrals and the tabulated potentials for the backbone dihedrals. We did not implement those differences, as it would require the usage of two external tabulated potentials for the copolymer, which is not feasible in Gromacs simulation package. Thus, due to simplicity, consistency, and feasibility, we implemented the same set of force field parameters for our model systems, and we only changed the chemical structure to obtain multiple stereoisomers.

Note also that the full implementation of the force field as reported in ref 45 of the original manuscript would be therefore possible only in the case of the pure PDLA and would lead to a reduced flexibility and thus to a higher terminal plateau in Figures 11(a) and 15(a) for PDLA100 and PDLA30, respectively. The full implementation, with the corresponding verification and the modification of the force field for the copolymers, is a topic of our current work.

Nevertheless, concerning the methodology and the algorithm for backmapping presented here, we would like to stress again one of our conclusions, namely, that the presented computational tools are versatile and do not rely on the actual force field parameters used for the polymer chains.

