# Supplementary material for: Physics-Informed Deep Learning Approach for Reintroducing Atomic Detail in Coarse-Grained Configurations of Multiple Poly(lactic acid) Stereoisomers
Source: J Chem Inf Model. 2024 Mar 1;64(6):1853–67. doi: 10.1021/acs.jcim.3c01870 (PMC10966642; doi:10.1021/acs.jcim.3c01870)
Supplement: Supplementary file 1 — ci3c01870_si_001.pdf [file ci3c01870_si_001.pdf]

Supporting Information

Physics-Informed Deep Learning Approach for Reintroducing Atomic Detail in Coarse-Grained Configurations of Multiple Poly(lactic acid) Stereoisomers

Eleftherios Christofi,[*,†] Petra Bačová,[*,‡] and Vagelis A. Harmandaris[*,†,¶,§]

†*Computation-based Science and Technology Research Center, The Cyprus Institute, 2121, Cyprus*

‡*Departamento de Ciencia de los Materiales e Ingeniería Metalúrgica y Química Inorgánica, Facultad de Ciencias, IMEYMAT, Campus Universitario Río San Pedro s/n., Puerto Real, Cádiz 11510, Spain*

¶*Department of Mathematics and Applied Mathematics, University of Crete, Heraklion, GR-71110, Greece*

§*Institute of Applied and Computational Mathematics, Foundation for Research and Technology - Hellas, GR-71110 Heraklion, Crete, Greece*

E-mail: e.christofi@cyi.ac.cy; petra.bacova@uca.es; v.harmandaris@cyi.ac.cy

1 Bond Vectors Model

In this Section we illustrate a comparison between the Reference model ($\lambda_{bv} = \lambda_{bl} = 1$ and $\lambda_{\mathbf{v}_0} = \lambda_{ba} = \lambda_{da} = 0$) and a model where we penalize only the bond vectors ($\lambda_{bv} = 1$ and

$\lambda_{\mathbf{v}_0} = \lambda_{bl} = \lambda_{ba} = \lambda_{da} = 0$). As can be seen in S1, the Reference model matches perfectly the target distributions, illustrating a significant deviation in comparison to the model trained with a loss function that penalizes only the bond vectors. Furthermore, the two models show similar behaviour on the prediction of bond angles and dihedral angles distributions (data not shown here).

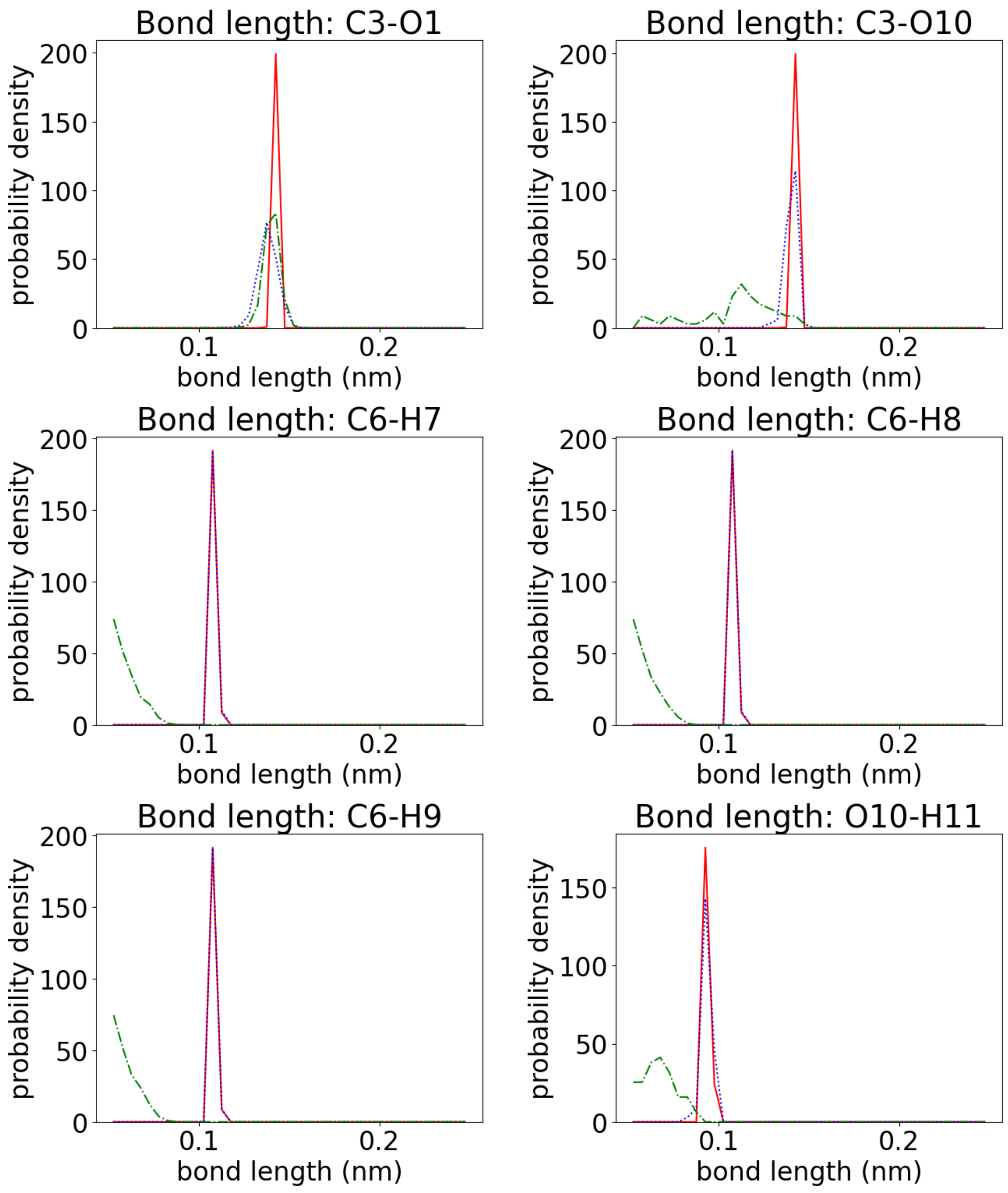

Figure S1: Comparison of bond lengths among target (red solid line), initial prediction for the reference model (blue dotted line), and the initial prediction for the model that penalize only the bond vectors (green dash-dotted line) for a 100-mer PLLA configuration.

2 100-mer

In this Section we present additional results concerning the atomistic structure of the 100-mer PLA systems predicted by the trained Machine Learning models. Specifically, we evaluate the performance of the trained models on three 100-mer systems (PLLA100, PDLA100, and Copo100). A comparison among the target, initial prediction, and the output of runEQ is provided for (a) intra- and intermonomeric dihedral angles; (b) intra- and intermolecular radial distribution functions specific for a number of different particles.

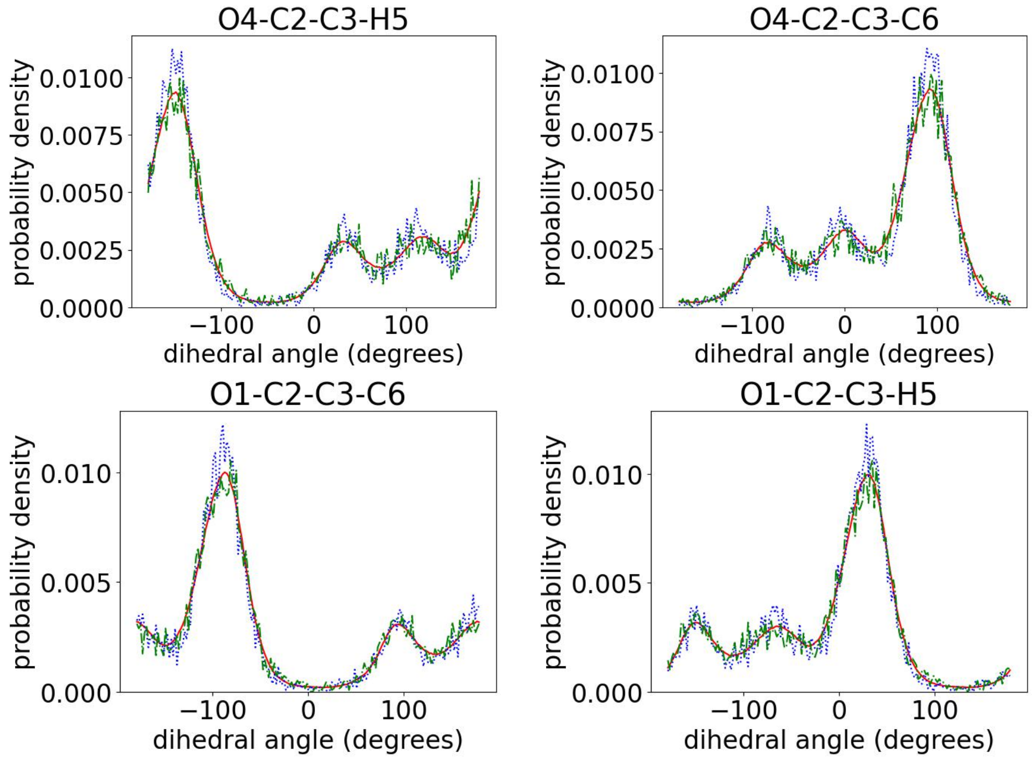

Figure S2: Comparison of intramonomeric dihedral angles among target (red solid line), initial prediction (blue dotted line), and the output of runEQ (green dash-dotted line) for a 100-mer PLLA configuration.

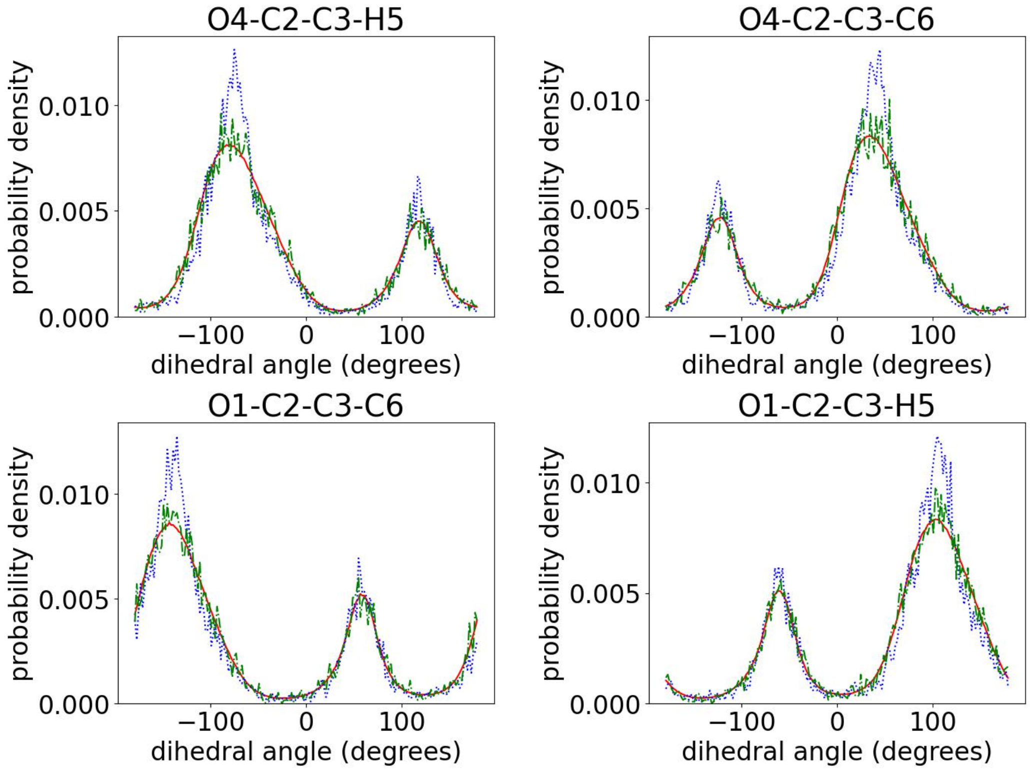

Figure S3: Comparison of intramonomeric dihedral angles among target (red solid line), initial prediction (blue dotted line), and the output of runEQ (green dash-dotted line) for a 100-mer PDLA configuration.

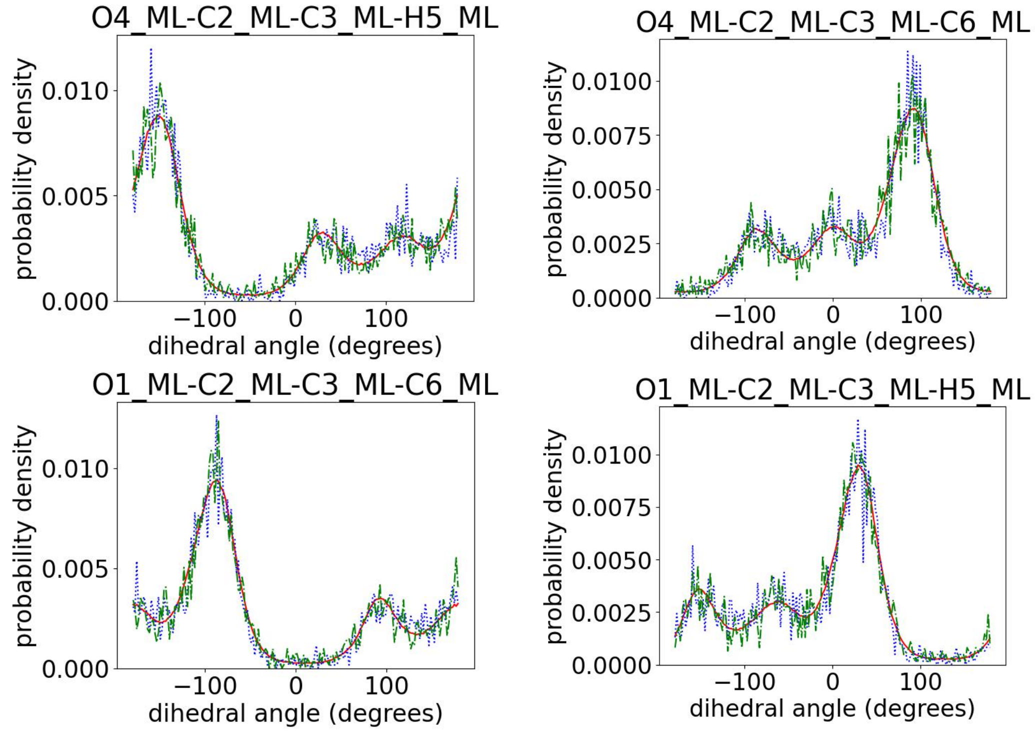

Figure S4: Comparison of intramonomeric dihedral angles among target (red solid line), initial prediction (blue dotted line), and the output of runEQ (green dash-dotted line) for a 100-mer PLA copolymer configuration, only for the L monomers.

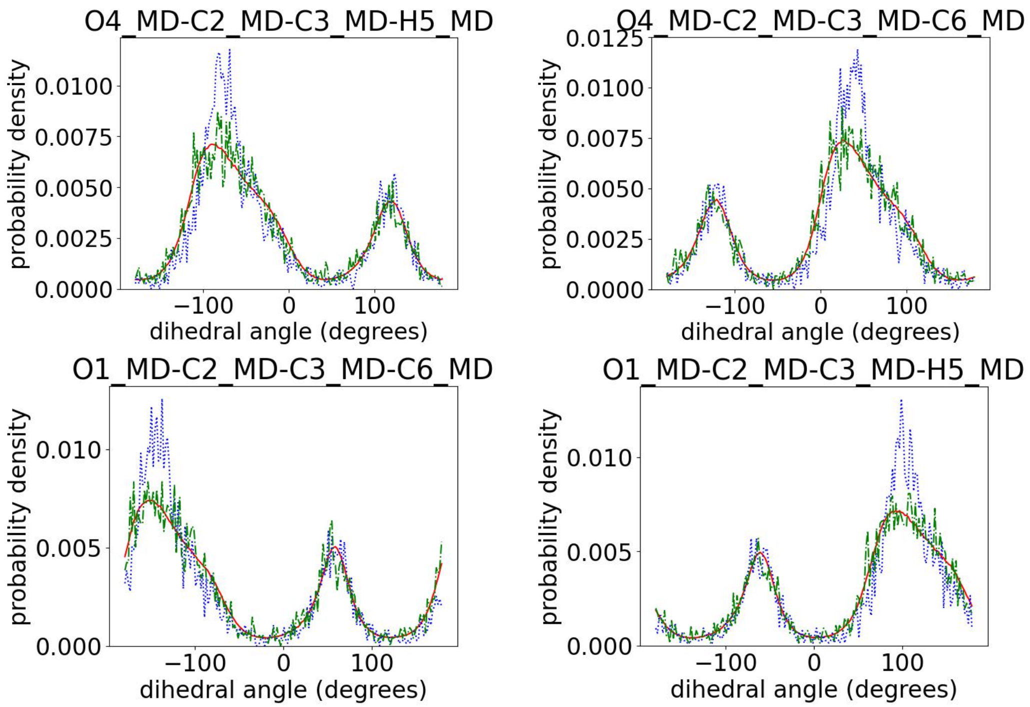

Figure S5: Comparison of intramonomeric dihedral angles among target (red solid line), initial prediction (blue dotted line), and the output of runEQ (green dash-dotted line) for a 100-mer PLA copolymer configuration, only for the D monomers.

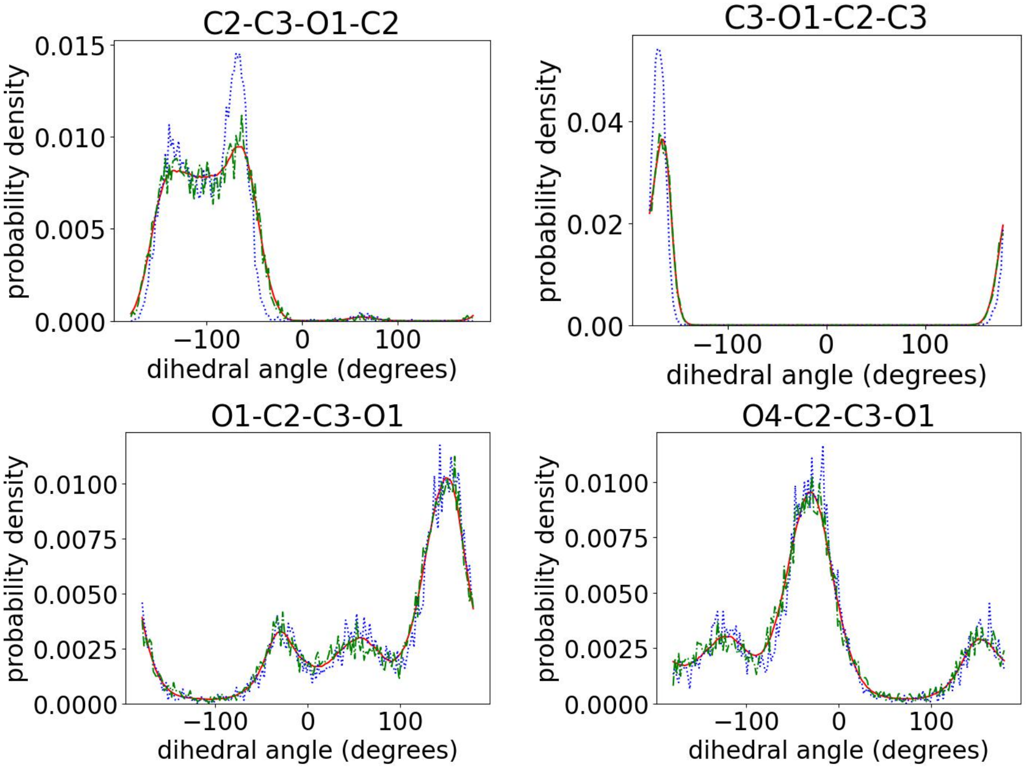

Figure S6: Comparison of the intermonomeric dihedral angles among target (red solid line), initial prediction (blue dotted line), and the output of runEQ (green dash-dotted line) for a 100-mer PLLA configuration.

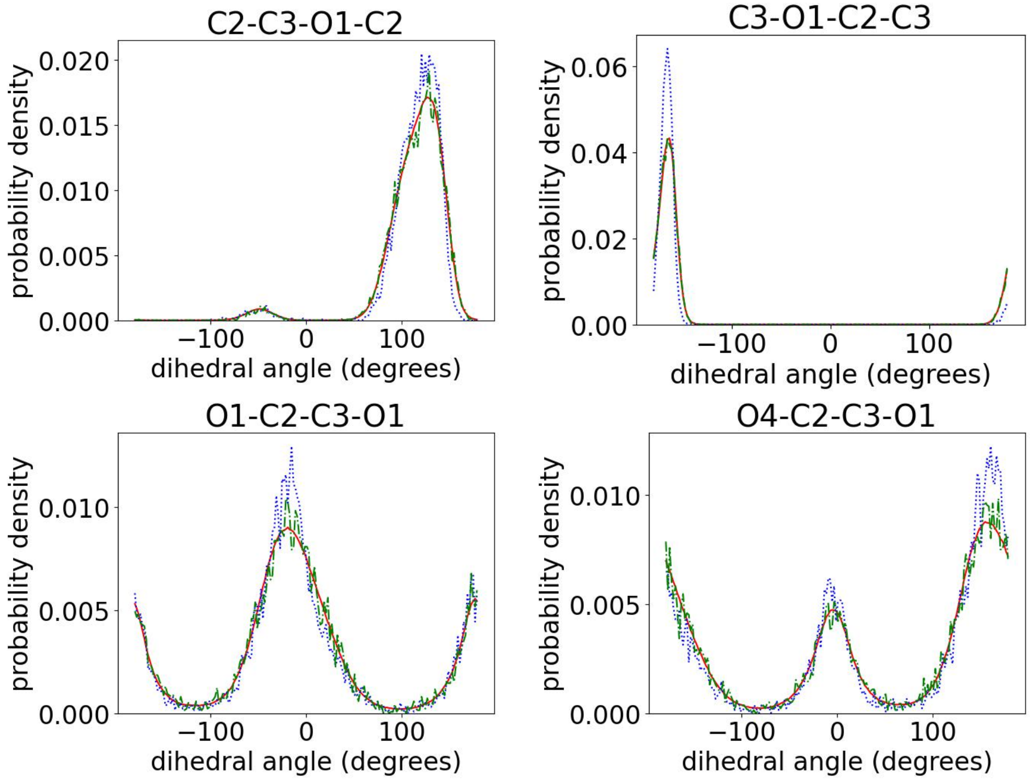

Figure S7: Comparison of the intermonomeric dihedral angles among target (red solid line), initial prediction (blue dotted line), and the output of runEQ (green dash-dotted line) for a 100-mer PDLA configuration.

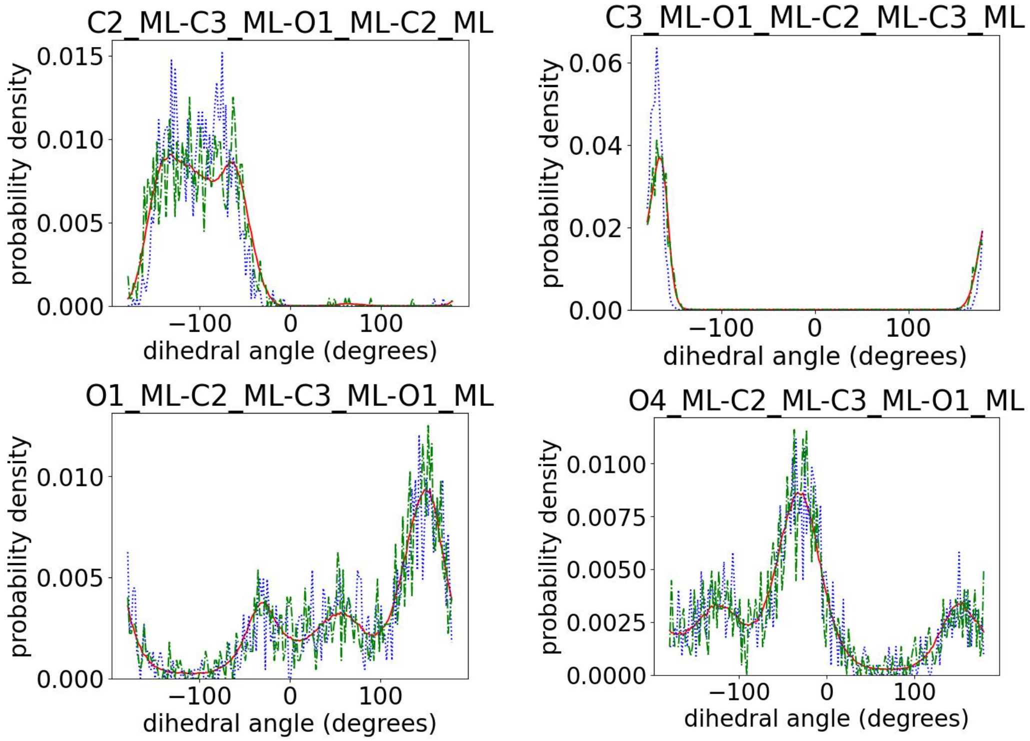

Figure S8: Comparison of intermonomeric dihedral angles among target (red solid line), initial prediction (blue dotted line), and the output of runEQ (green dash-dotted line) for a 100-mer PLA copolymer configuration, only for the L monomers.

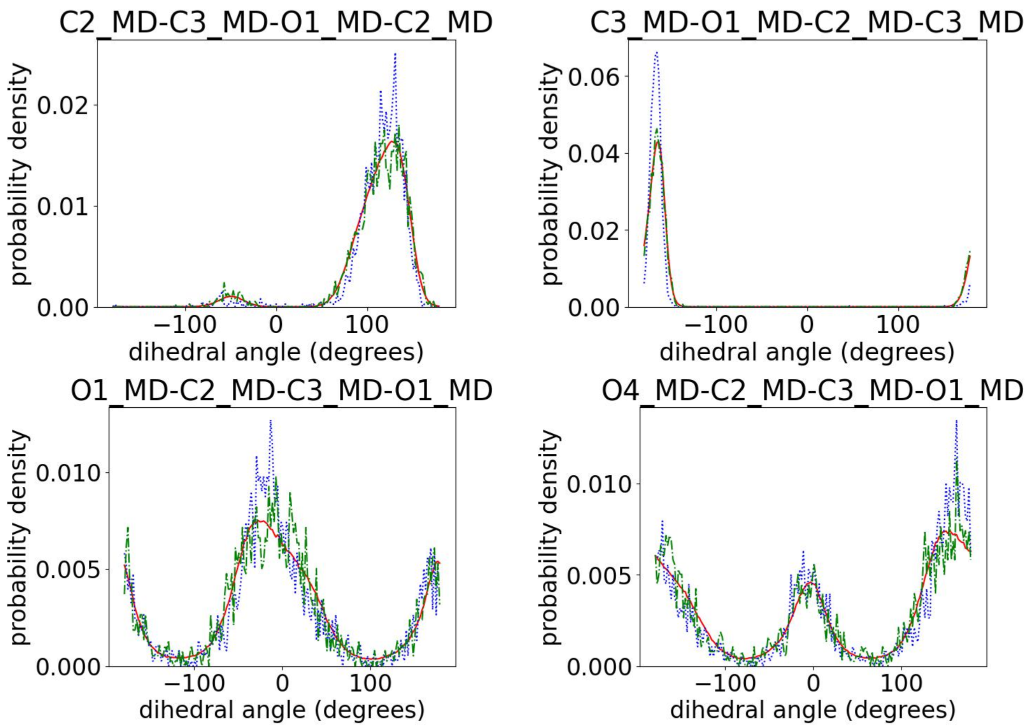

Figure S9: Comparison of intermonomeric dihedral angles among target (red solid line), initial prediction (blue dotted line), and the output of runEQ (green dash-dotted line) for a 100-mer PLA copolymer configuration, only for the D monomers.

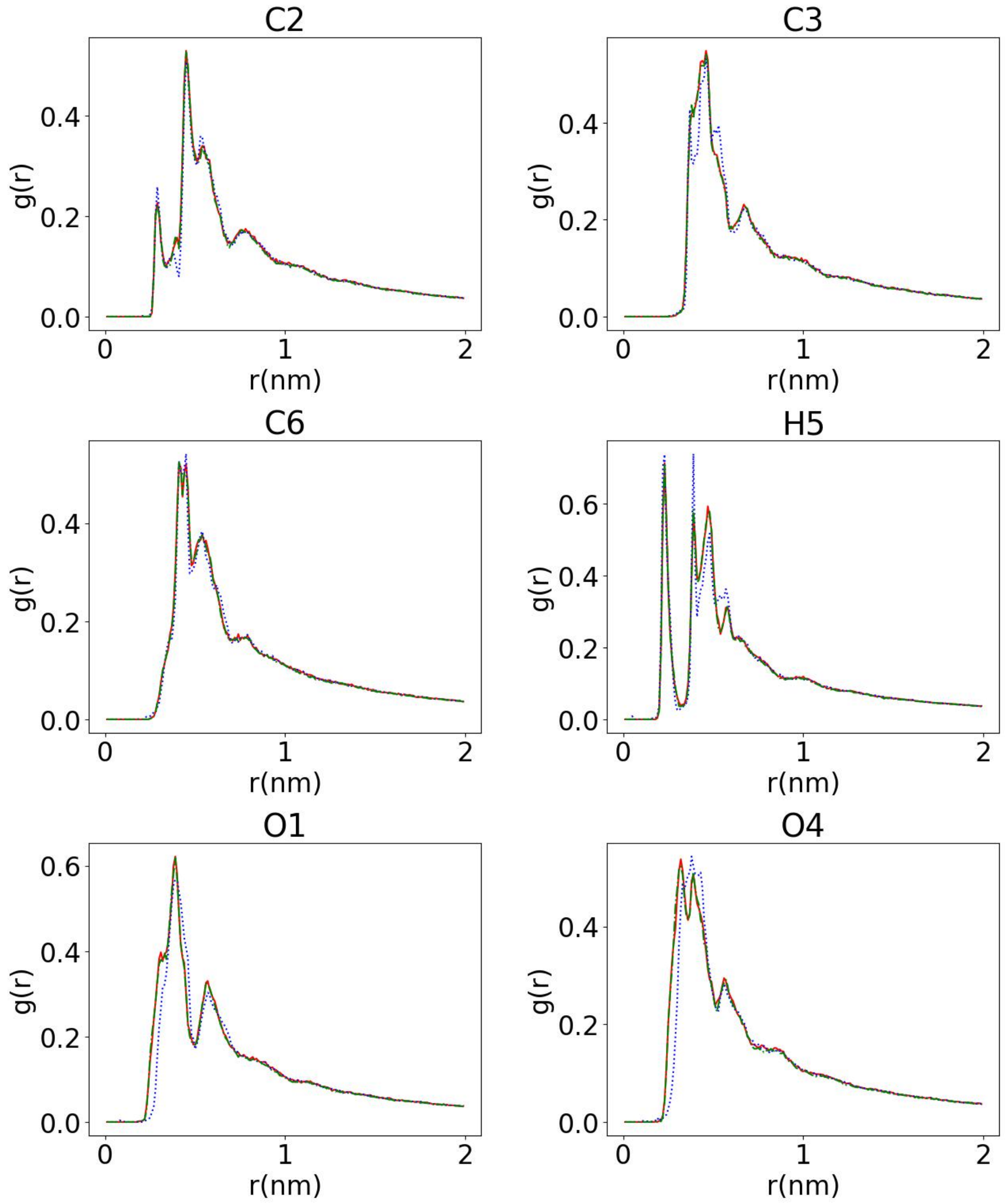

Figure S10: Comparison of intramolecular radial distributions around the given atoms among target (red solid line), initial prediction (blue dotted line), and the output of runEQ (green dash-dotted line) for a 100-mer PLLA configuration.

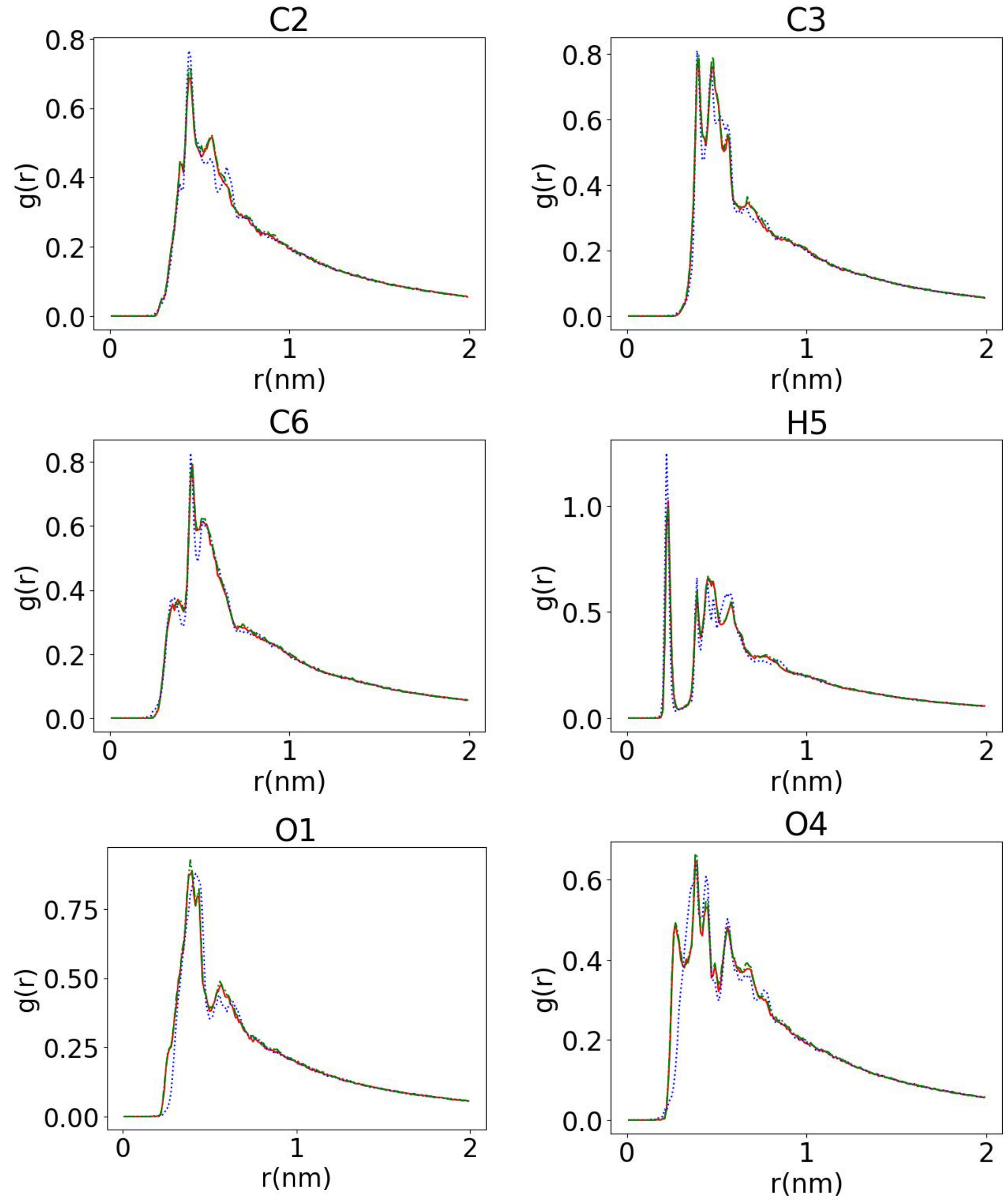

Figure S11: Comparison of intramolecular radial distributions around the given atoms among target (red solid line), initial prediction (blue dotted line), and the output of runEQ (green dash-dotted line) for a 100-mer PDLA configuration.

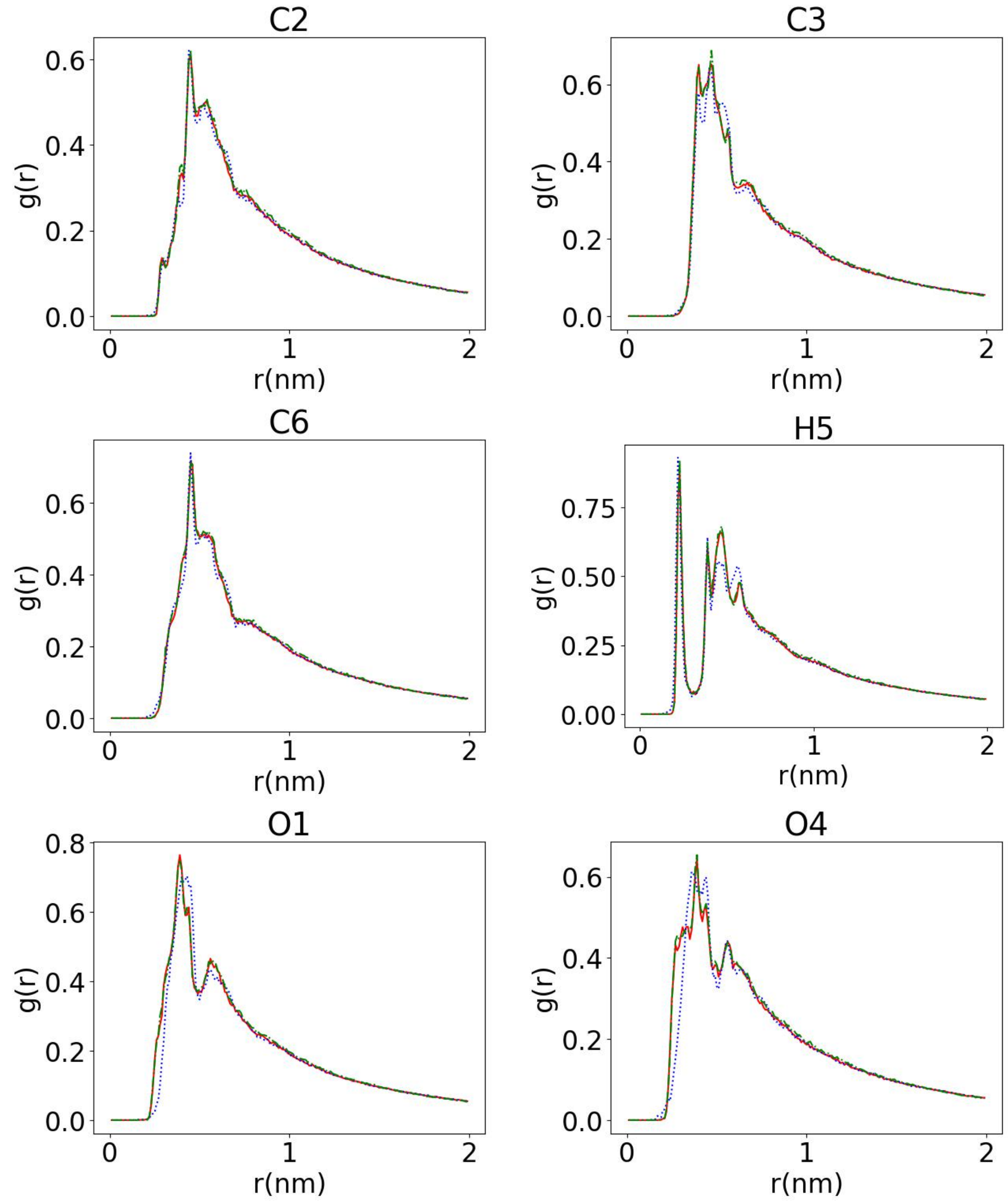

Figure S12: Comparison of intramolecular radial distributions around the given atoms among target (red solid line), initial prediction (blue dotted line), and the output of runEQ (green dash-dotted line) for a 100-mer PLA copolymer configuration.

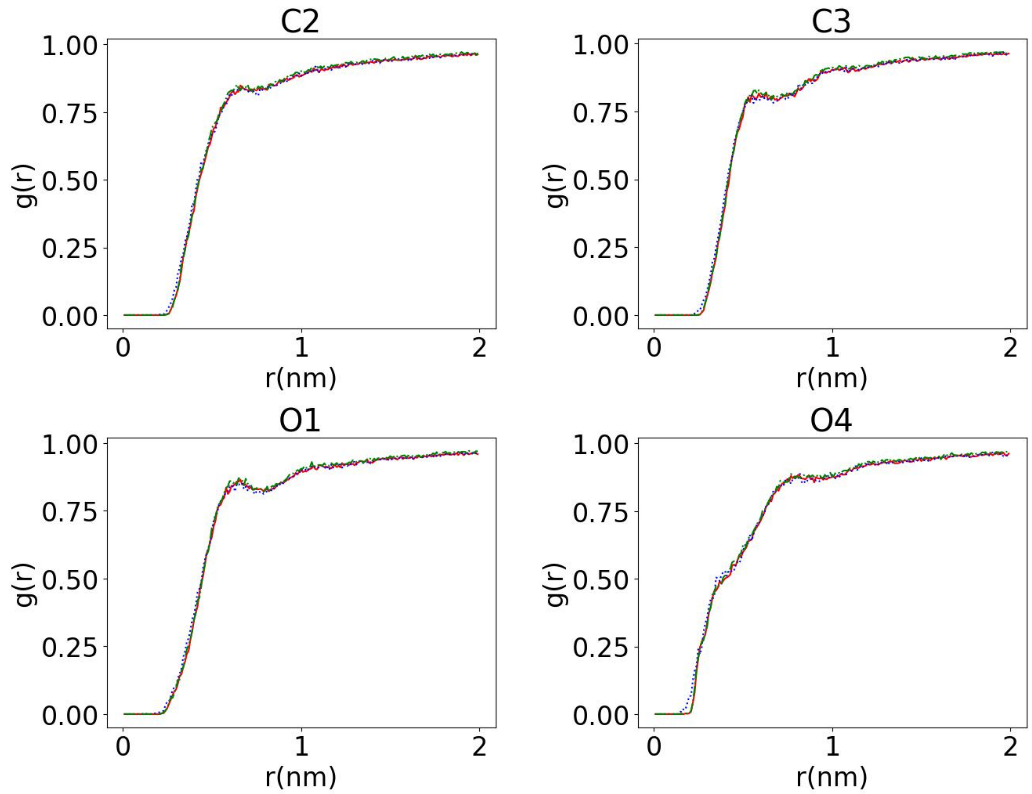

Figure S13: Comparison of intermolecular radial distributions around the given atoms among target (red solid line), initial prediction (blue dotted line), and the output of runEQ (green dash-dotted line) for a 100-mer PLLA configuration.

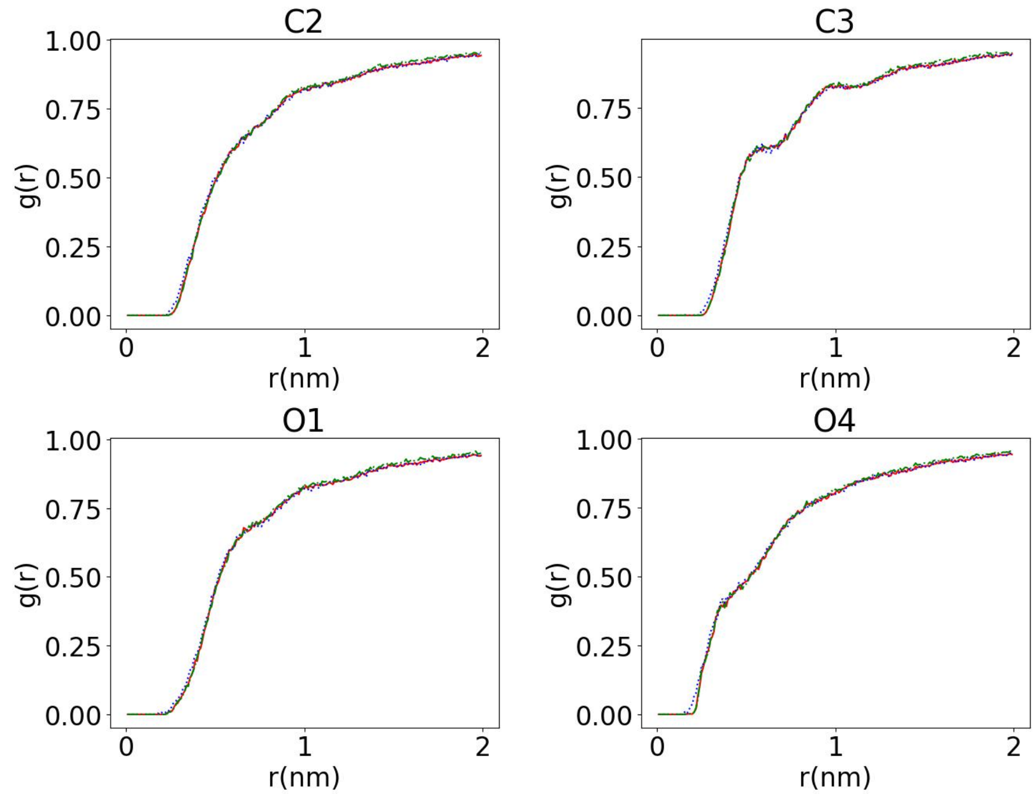

Figure S14: Comparison of intermolecular radial distributions around the given atoms among target (red solid line), initial prediction (blue dotted line), and the output of runEQ (green dash-dotted line) for a 100-mer PDLA configuration.

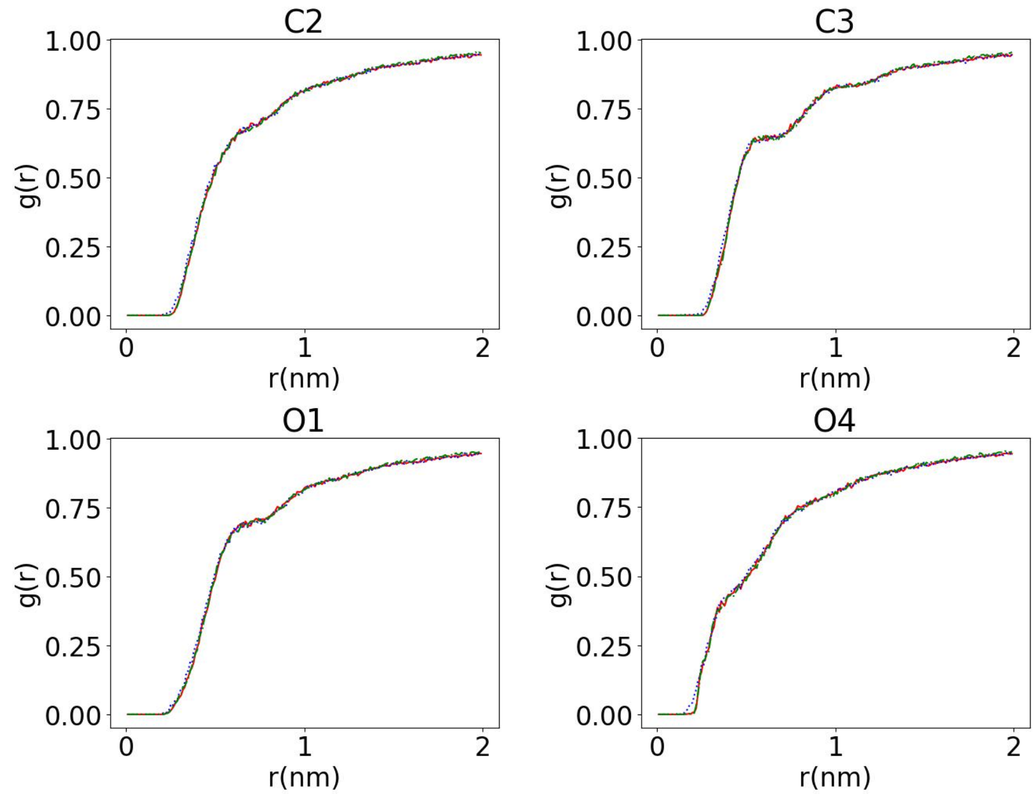

Figure S15: Comparison of intermolecular radial distributions around the given atoms among target (red solid line), initial prediction (blue dotted line), and the output of runEQ (green dash-dotted line) for a 100-mer PLA copolymer configuration.

3 Copo100RAND and Copo100HOM

In this Section we investigate the transferability of the trained Machine Learning models for PLA 100-mer copolymers prepared by a different backmapping strategy. Copo100HOM has the same sequence of stereoisomers per chain as the target system, but the neural network was trained solely on 100-mer homopolymer systems, while Copo100RAND was trained using all the available 100-mer systems, but has random stereochemistry with the same D content (55%) in every chain. A comparison among the target data and the initial predictions of Copo100RAND and Copo100HOM is provided for (a) intra- and intermonomeric dihedral angles; (b) intra- and intermolecular radial distribution functions specific for a number of different particles.

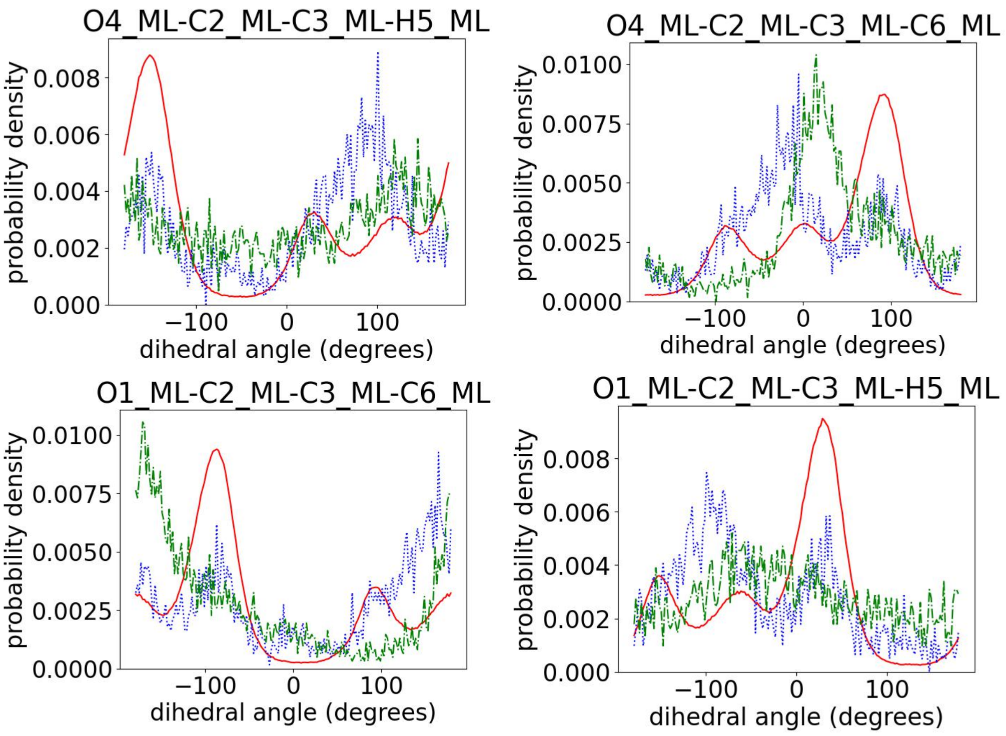

Figure S16: Comparison of intramonomeric dihedral angles among target (red solid line) and the initial predictions for Copo100RAND (blue dotted line) and Copo100HOM (green dash-dotted line) for a 100-mer PLA copolymer configuration, only for the L monomers.

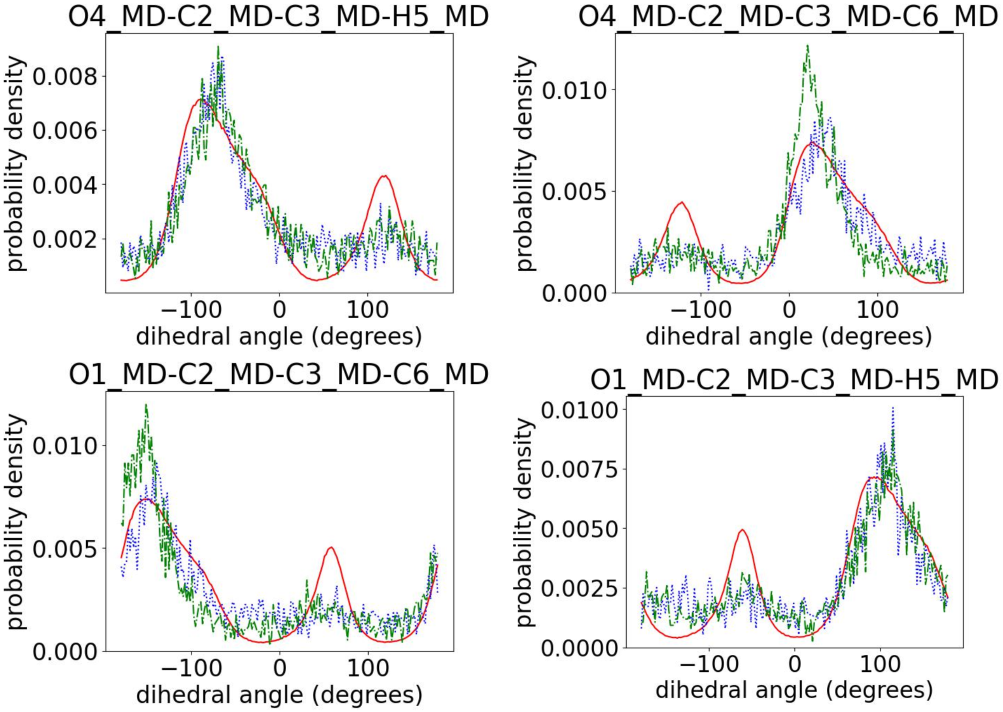

Figure S17: Comparison of intramonomeric dihedral angles among target (red solid line) and the initial predictions for Copo100RAND (blue dotted line) and Copo100HOM (green dash-dotted line) for a 100-mer PLA copolymer configuration, only for the D monomers.

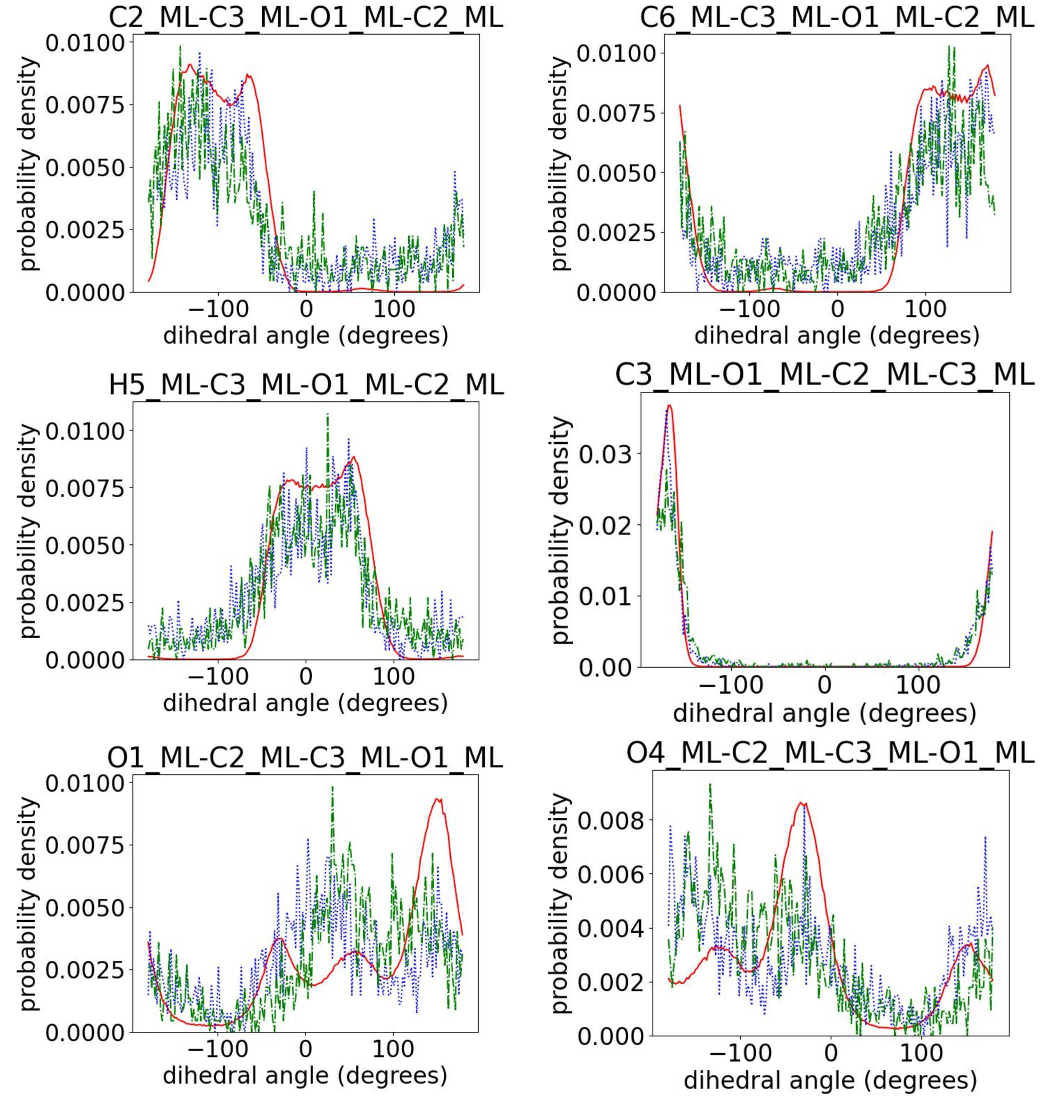

Figure S18: Comparison of intermonomeric dihedral angles among target (red solid line) and the initial predictions for Copo100RAND (blue dotted line) and Copo100HOM (green dash-dotted line) for a 100-mer PLA copolymer configuration, only for the L monomers.

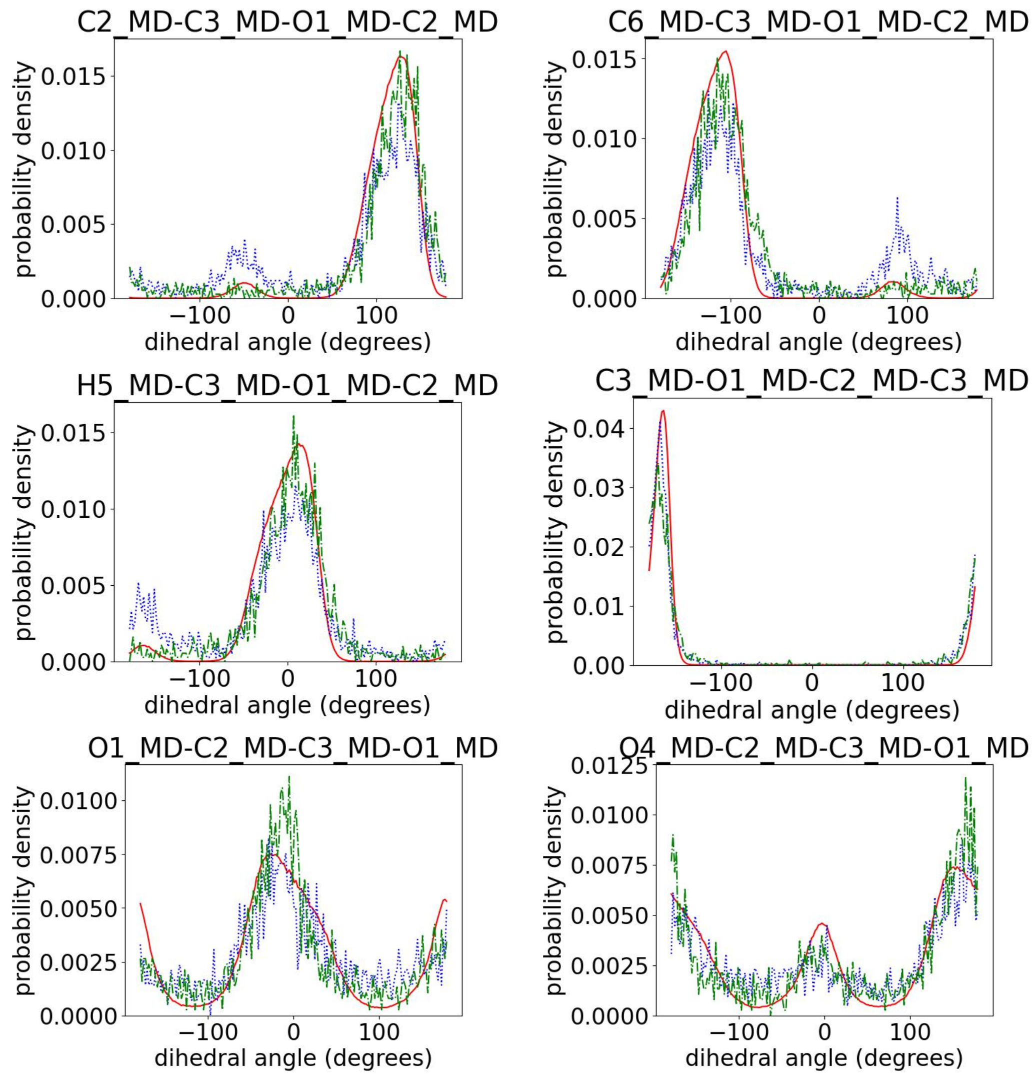

Figure S19: Comparison of intermonomeric dihedral angles among target (red solid line) and the initial predictions for Copo100RAND (blue dotted line) and Copo100HOM (green dash-dotted line) for a 100-mer PLA copolymer configuration, only for the D monomers.

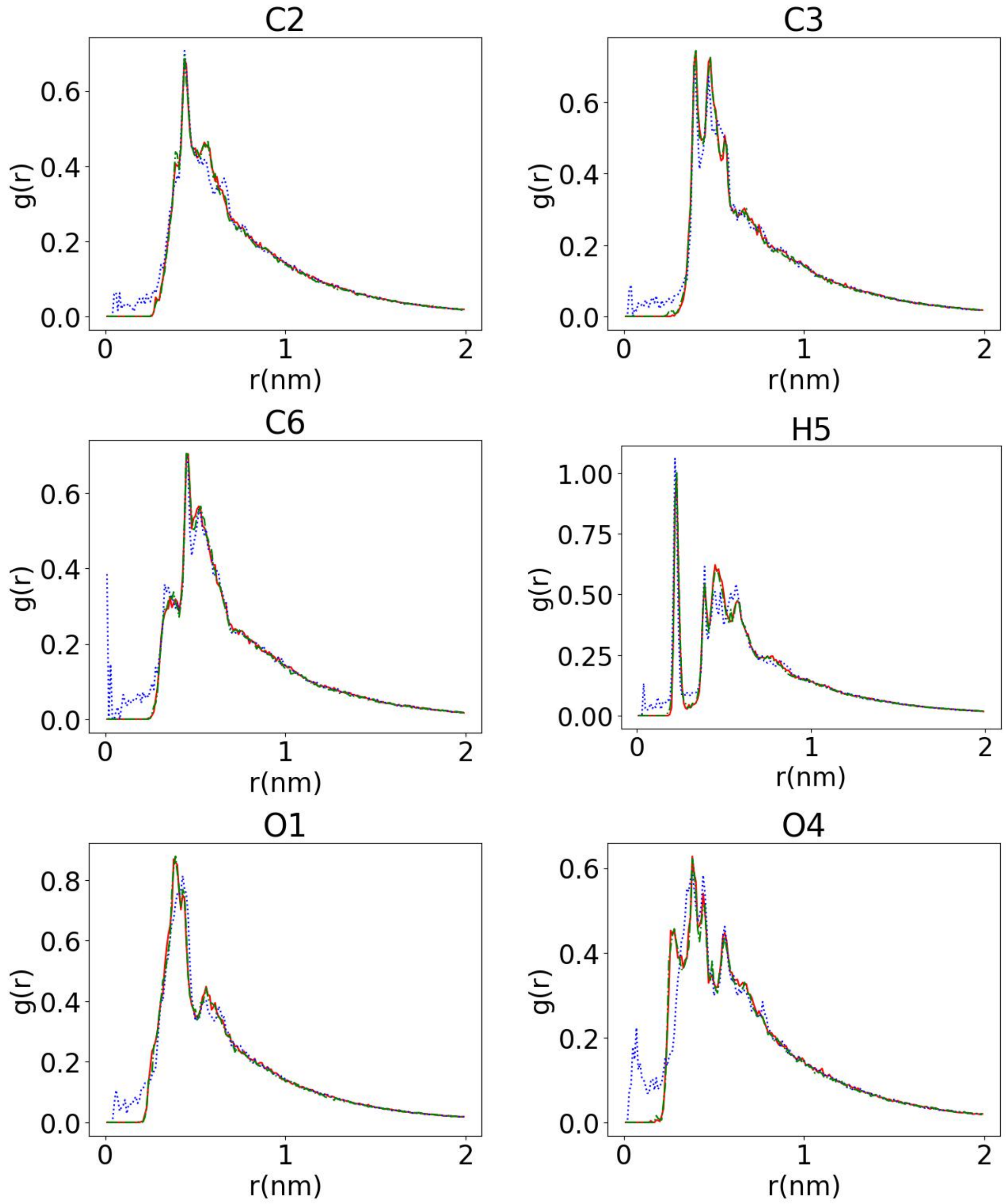

Figure S20: Comparison of intramolecular radial distributions around the given atoms among target (red solid line) and initial predictions of Copo100HOM (blue dotted line) and Copo100RAND (green dash-dotted line) models for a 100-mer PLA copolymer configuration.

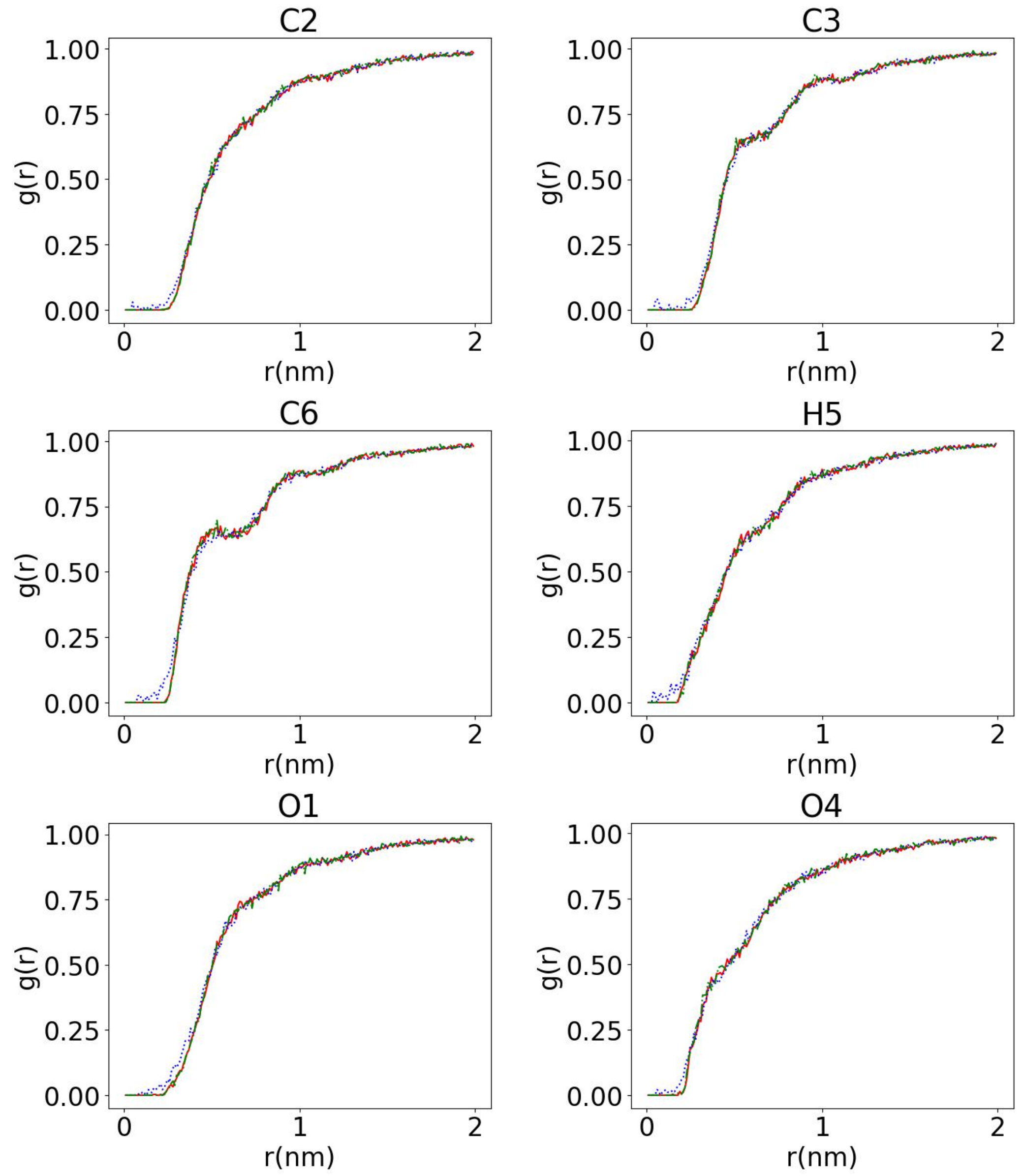

Figure S21: Comparison of intermolecular radial distributions around the given atoms among target (red solid line) and initial predictions of Copo100HOM (blue dotted line) and Copo100RAND (green dash-dotted line) models for a 100-mer PLA copolymer configuration.

4 30-mer

In this Section we examine the transferability of the derived models across different molecular weights (chain lenghts) by presenting additional results. Specifically, we apply the trained models to 30-mer PLA systems instead of the 100-mer used for the training. A comparison among the target, initial prediction, and the output of runEQ is provided for (a) intra- and intermonomeric dihedral angles; (b) intra- and intermolecular radial distribution functions specific for a number of different particles.

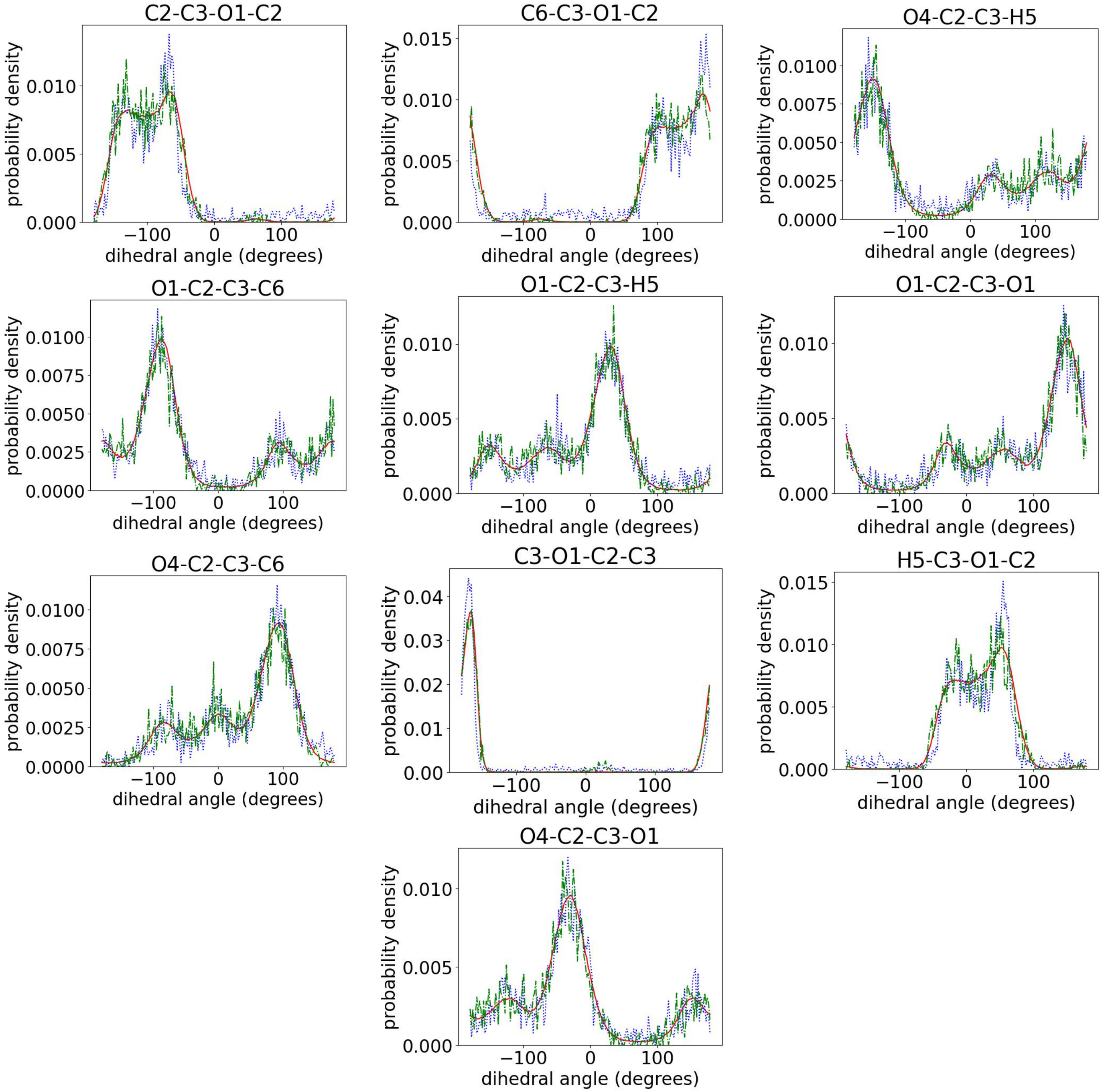

Figure S22: Comparison of dihedral angles among target (red solid line), initial prediction (blue dotted line), and the output of runEQ (green dash-dotted line) for a 30-mer PLLA configuration.

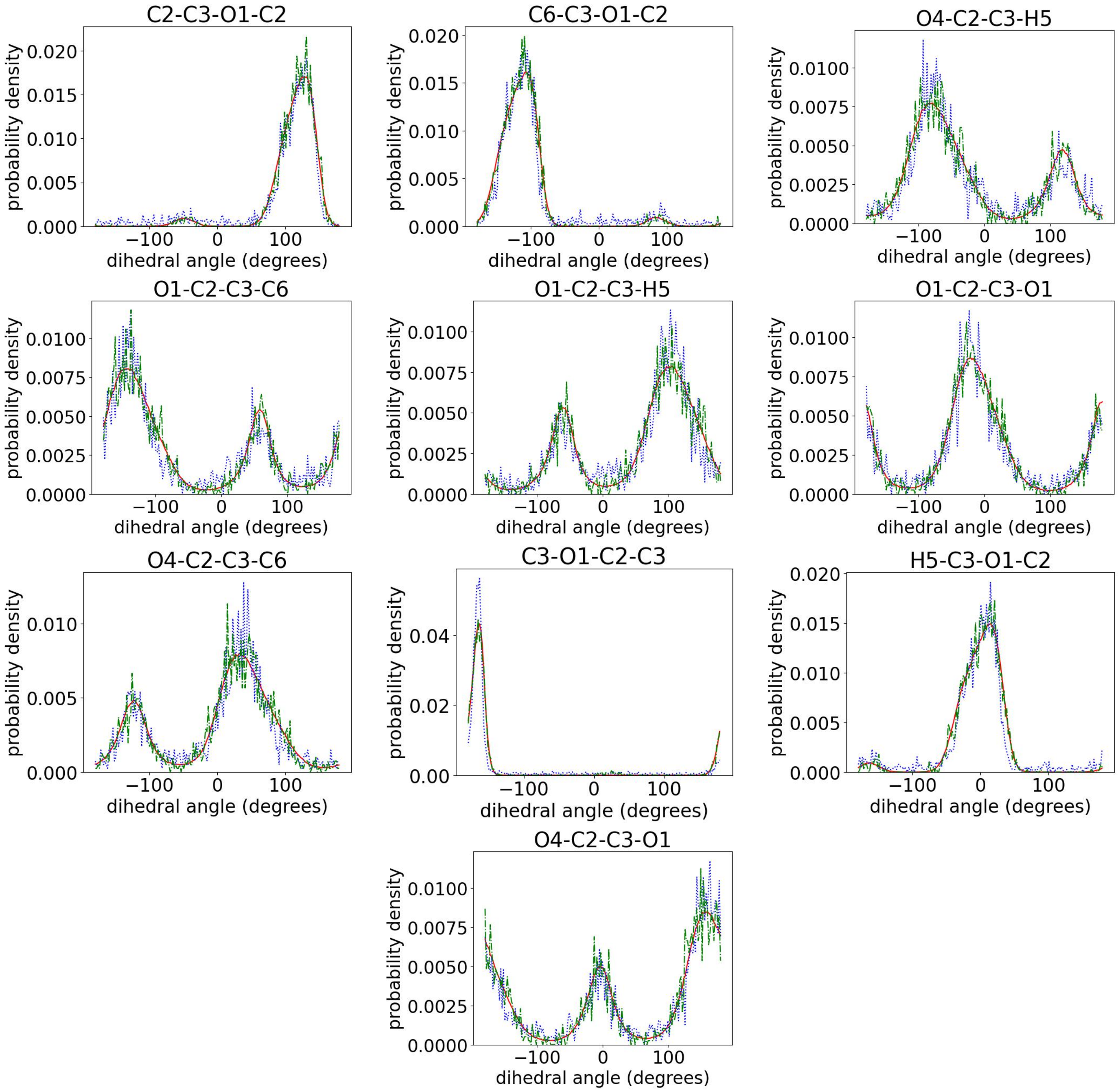

Figure S23: Comparison of dihedral angles among target (red solid line), initial prediction (blue dotted line), and the output of runEQ (green dash-dotted line) for a 30-mer PDLA configuration.

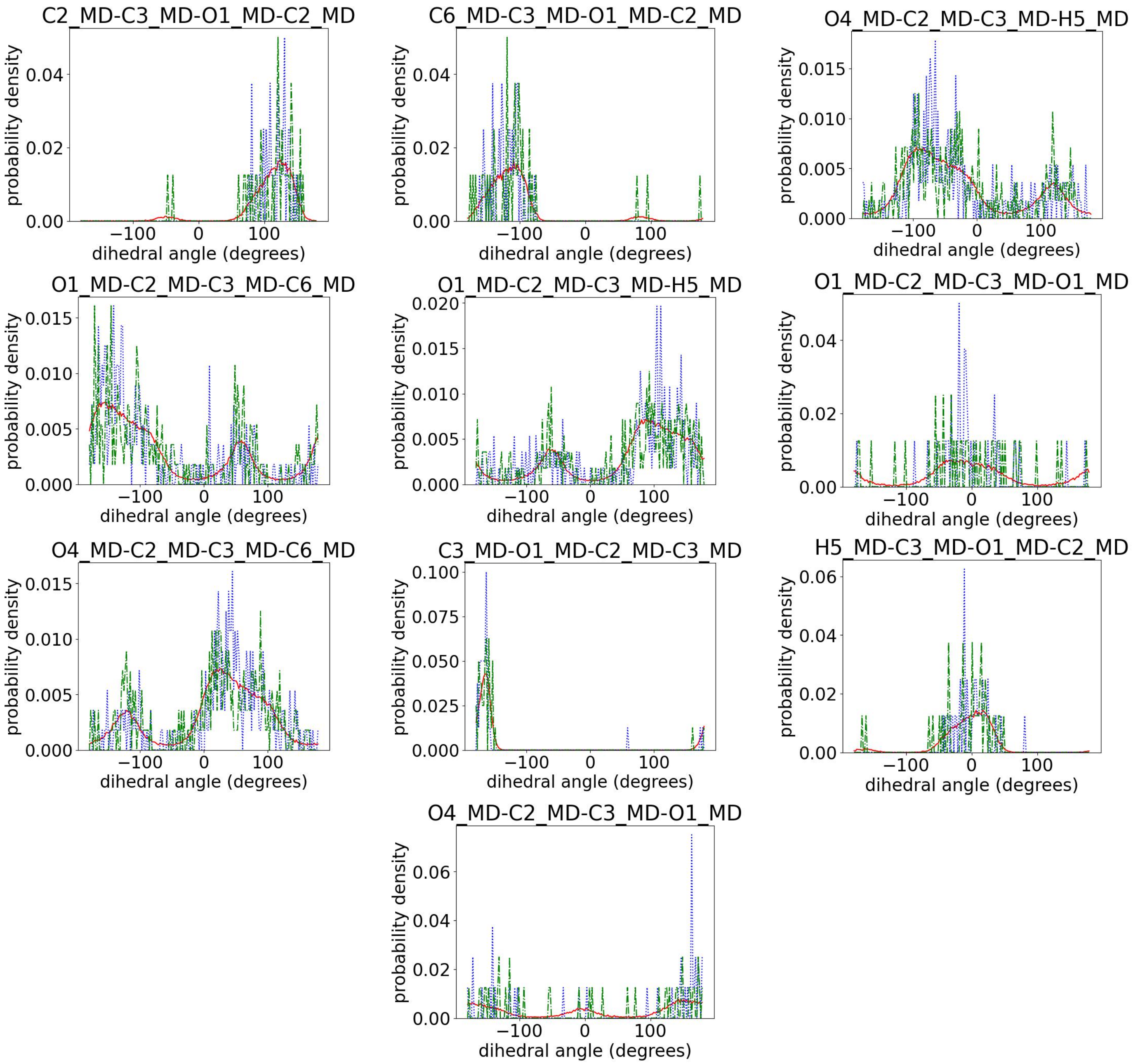

Figure S24: Comparison of dihedral angles among target (red solid line), initial prediction (blue dotted line), and the output of runEQ (green dash-dotted line) for a 30-mer PLA copolymer configuration, only for the D monomers.

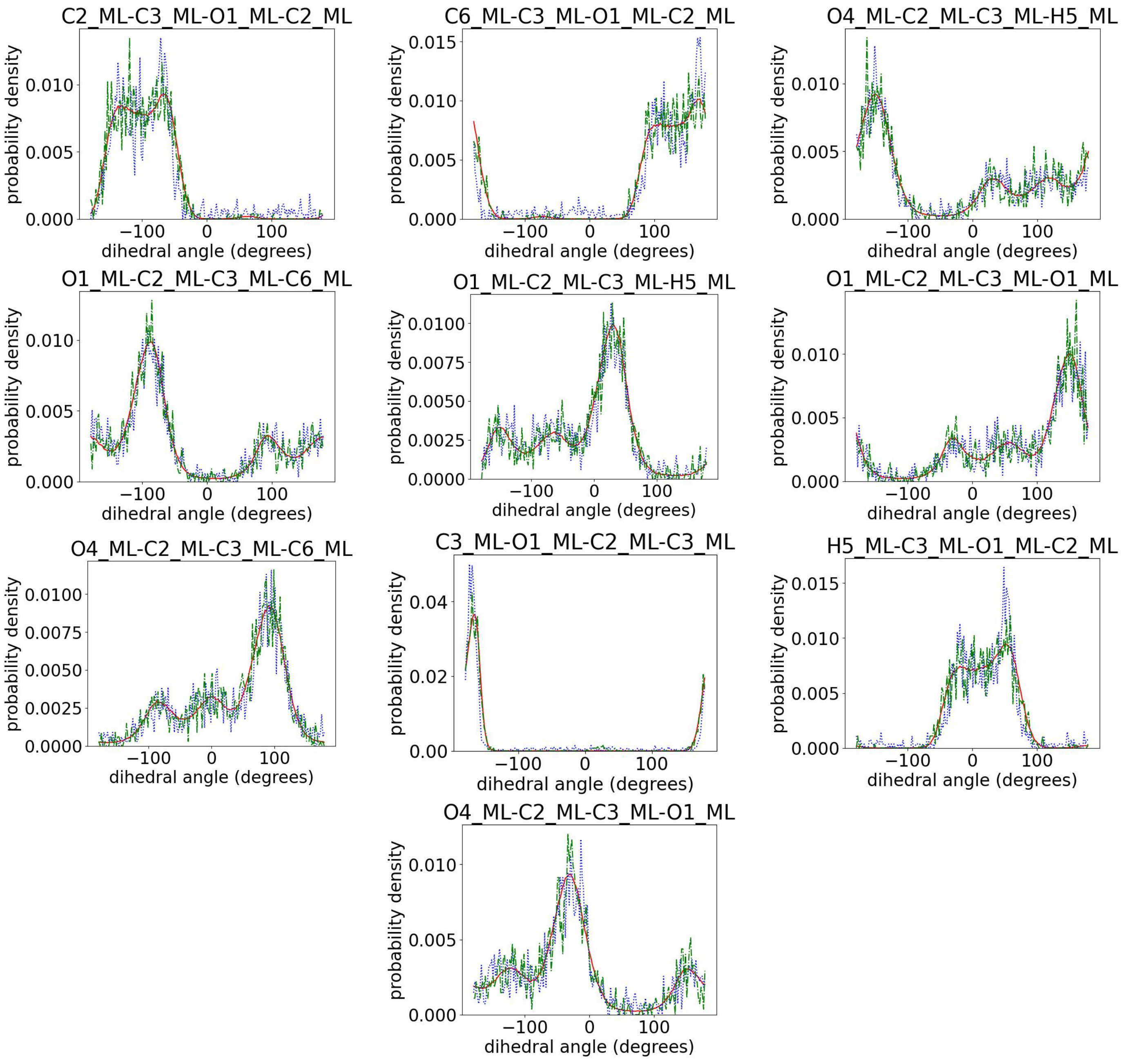

Figure S25: Comparison of dihedral angles among target (red solid line), initial prediction (blue dotted line), and the output of runEQ (green dash-dotted line) for a 30-mer PLA copolymer configuration, only for the L monomers.

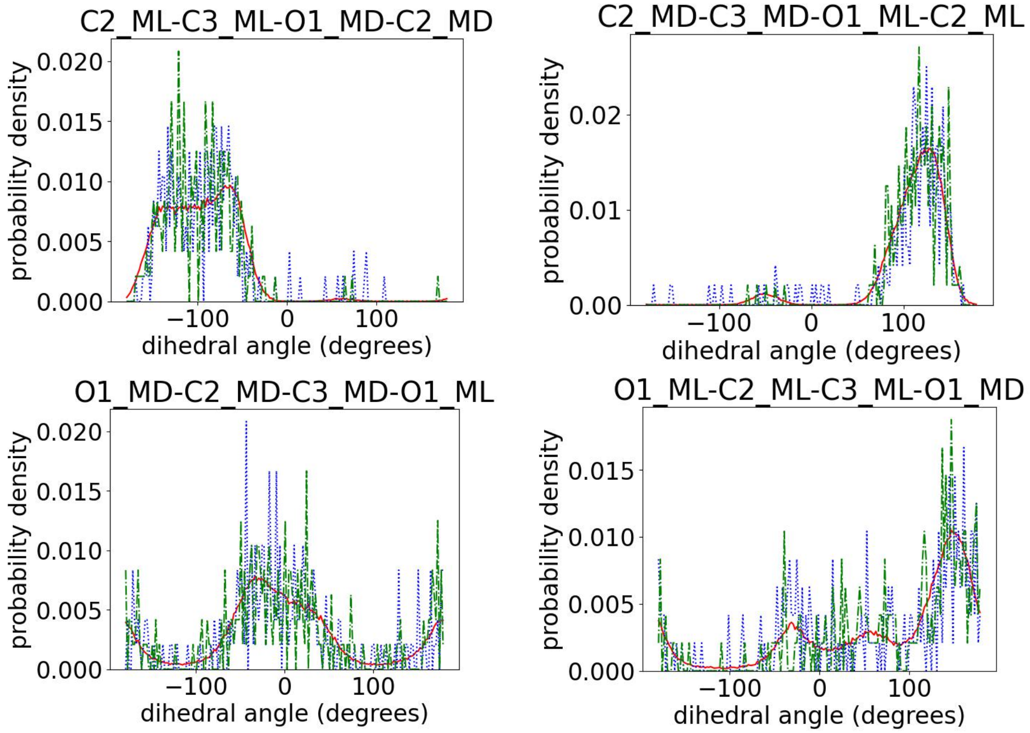

Figure S26: Comparison of dihedral angles among target (red solid line), initial prediction (blue dotted line), and the output of runEQ (green dash-dotted line) for a 30-mer PLA copolymer configuration, only for the distributions between D and L monomers.

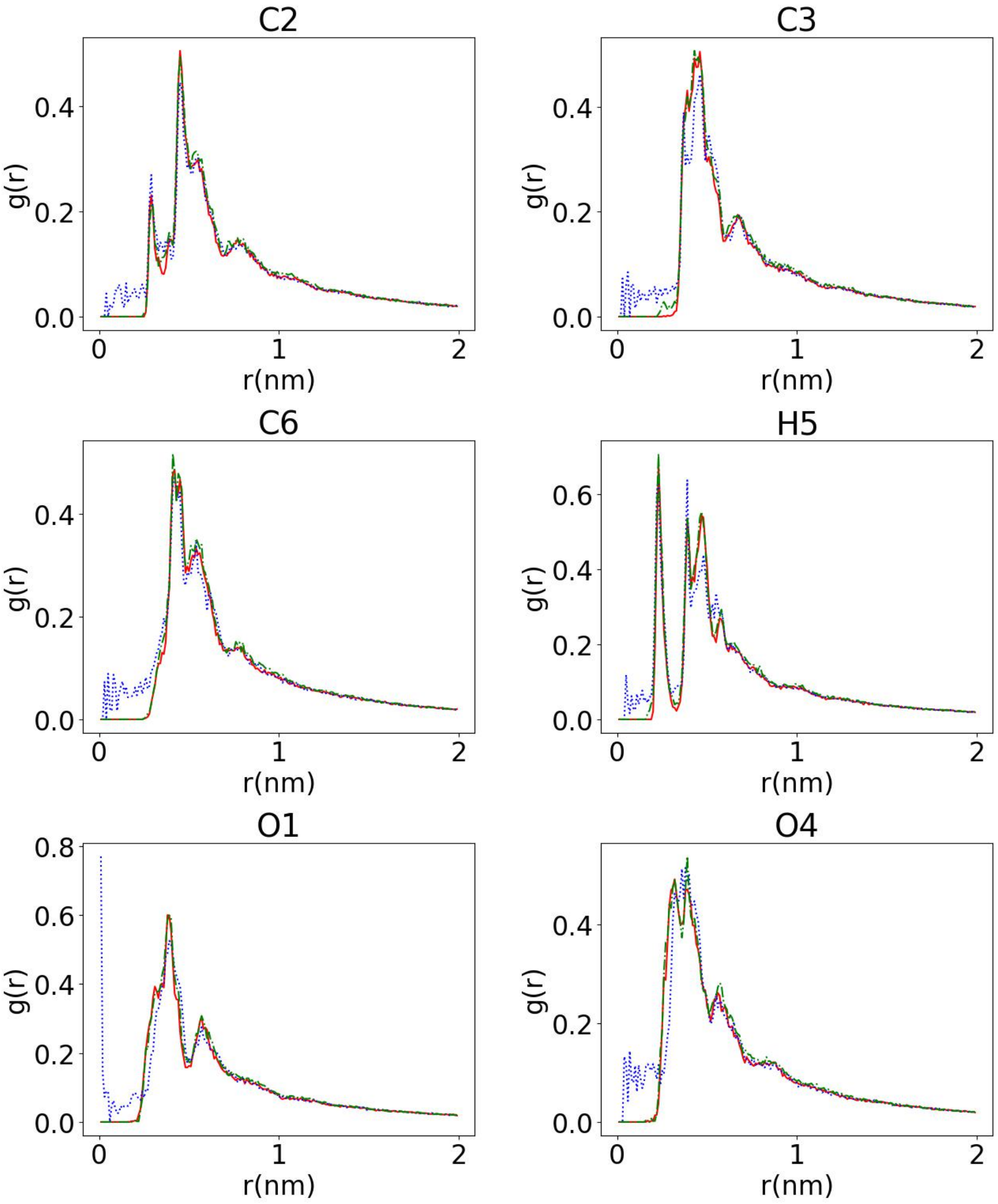

Figure S27: Comparison of intramolecular radial distributions around the given atoms among target (red solid line), initial prediction (blue dotted line), and the output of runEQ (green dash-dotted line) for a 30-mer PLLA configuration.

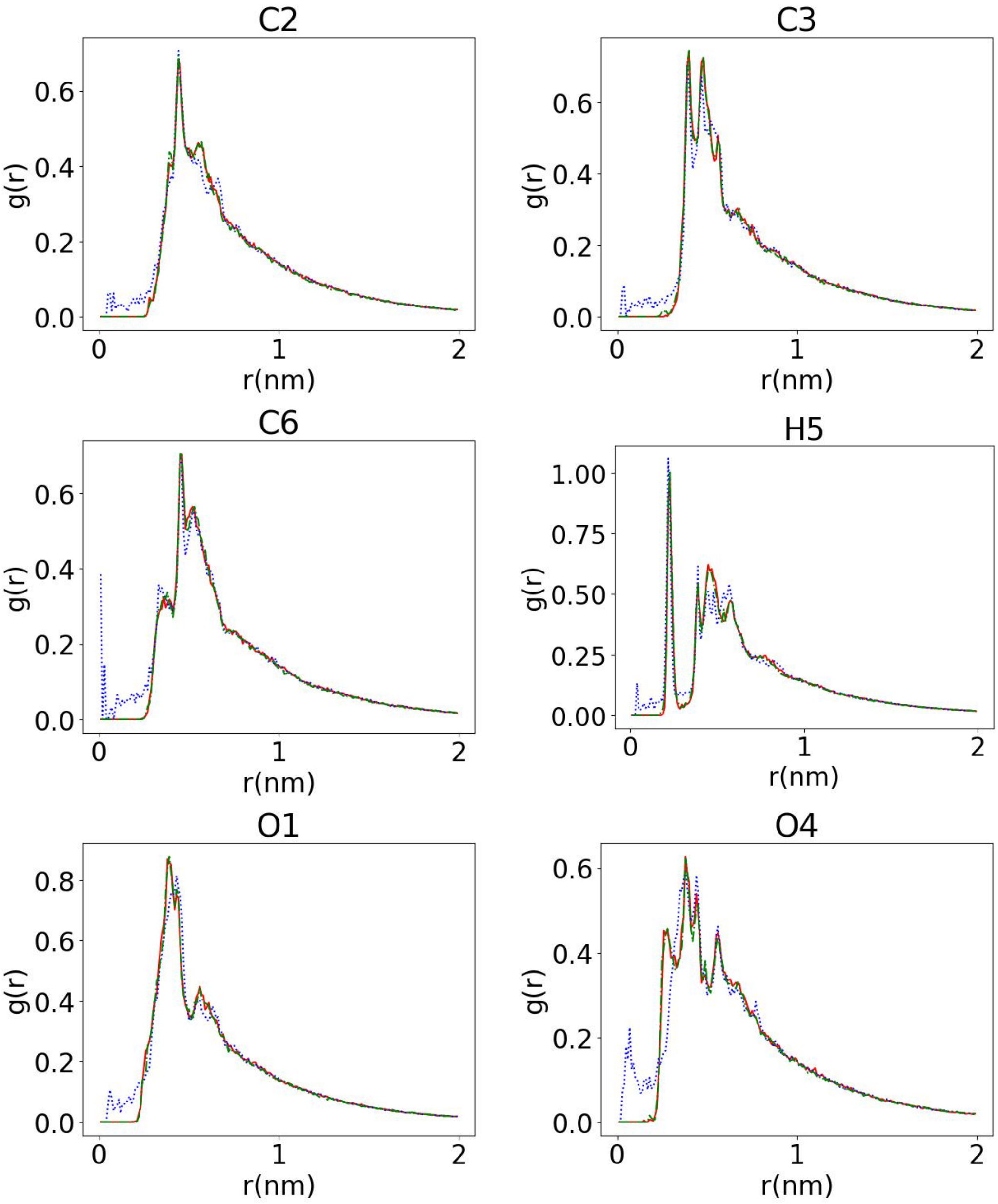

Figure S28: Comparison of intramolecular radial distributions around the given atoms among target (red solid line), initial prediction (blue dotted line), and the output of runEQ (green dash-dotted line) for a 30-mer PDLA configuration.

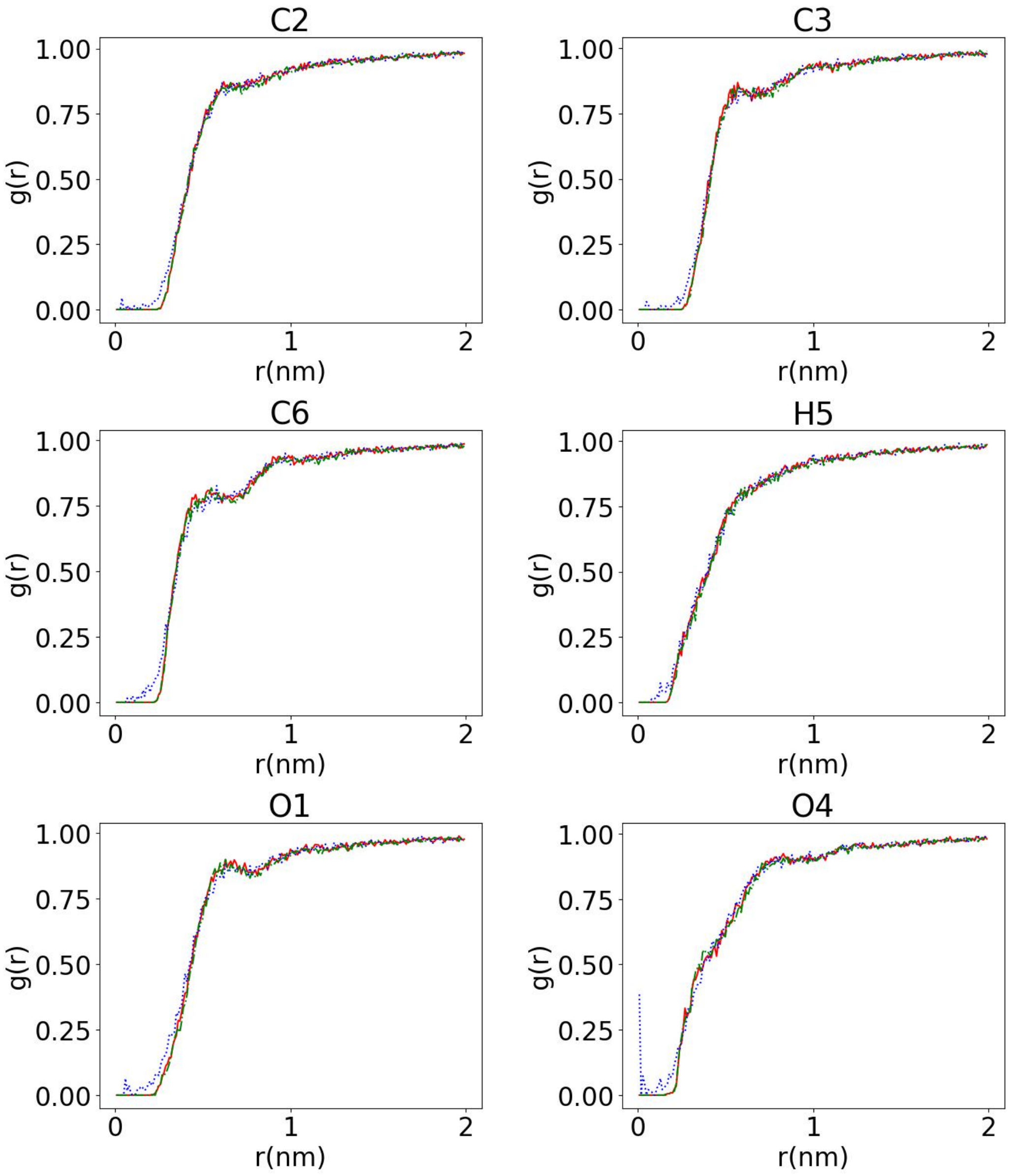

Figure S29: Comparison of intermolecular radial distributions around the given atoms among target (red solid line), initial prediction (blue dotted line), and the output of runEQ (green dash-dotted line) for a 30-mer PLLA configuration.

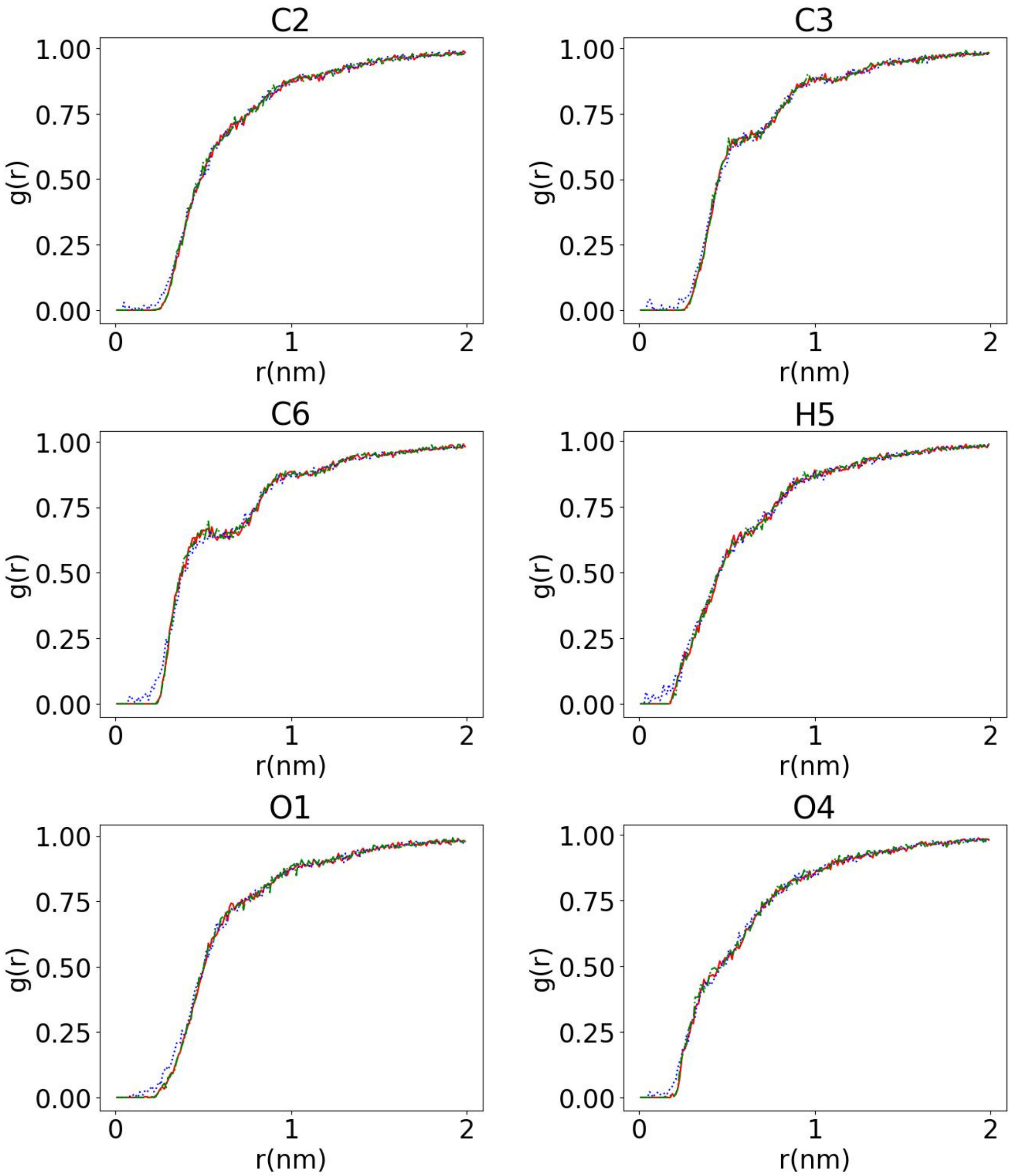

Figure S30: Comparison of intermolecular radial distributions around the given atoms among target (red solid line), initial prediction (blue dotted line), and the output of runEQ (green dash-dotted line) for a 30-mer PDLA configuration.

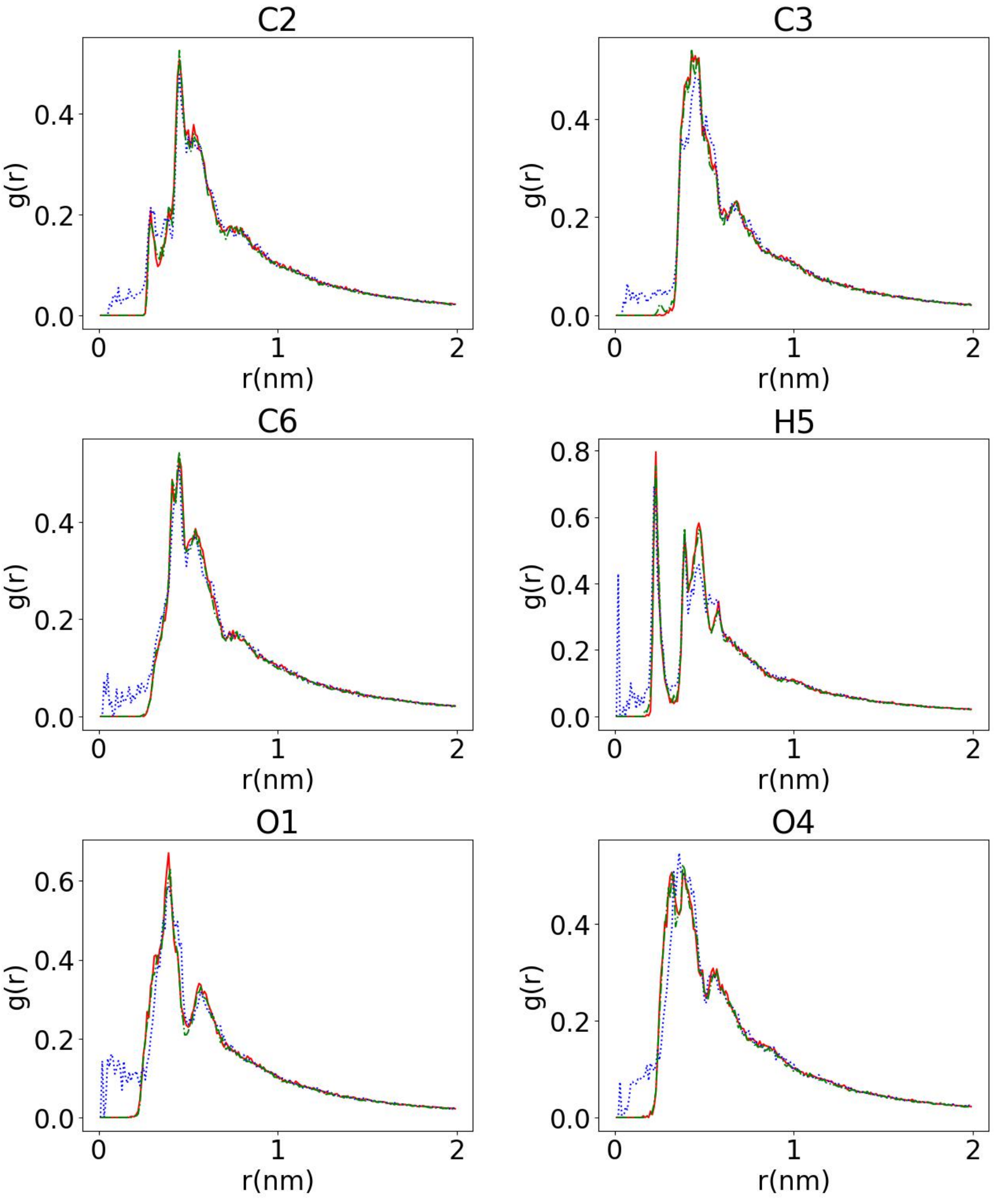

Figure S31: Comparison of intramolecular radial distributions around the given atoms among target (red solid line), initial prediction (blue dotted line), and the output of runEQ (green dash-dotted line) for a 30-mer PLA copolymer configuration.

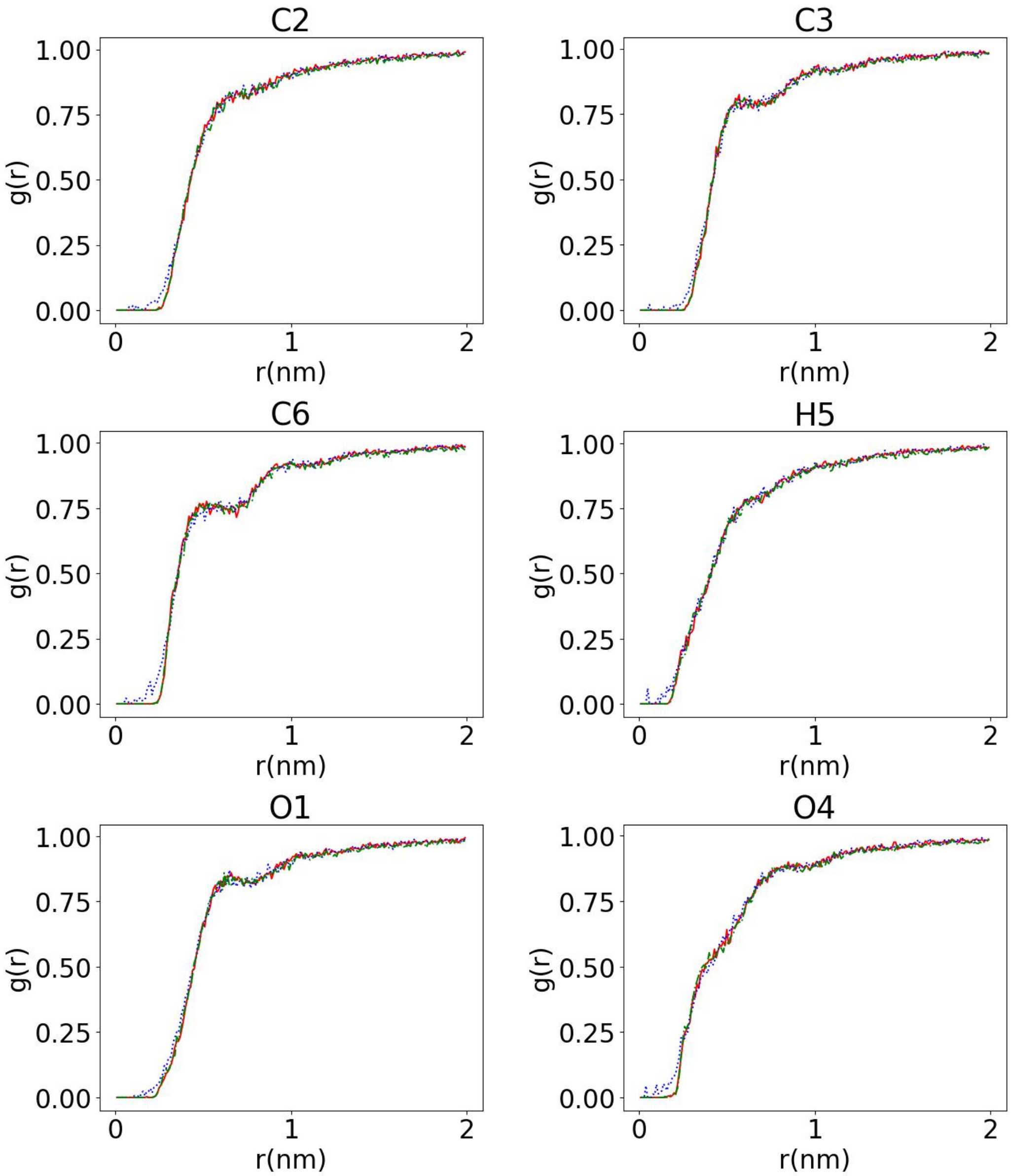

Figure S32: Comparison of intermolecular radial distributions around the given atoms among target (red solid line), initial prediction (blue dotted line), and the output of runEQ (green dash-dotted line) for a 30-mer PLA copolymer configuration.
